# Mock Modularity at Work, or Black Holes in a Forest

**DOI:** 10.3390/e27070719

**Published:** 2025-07-02

**Authors:** Sergei Alexandrov

**Affiliations:** Laboratoire Charles Coulomb (L2C), Université de Montpellier, CNRS, F-34095 Montpellier, France; sergey.alexandrov@umontpellier.fr

**Keywords:** mock modular form, black hole, Calabi–Yau, BPS index, Donaldson–Thomas invariant, compactification, Vafa–Witten theory, topological string

## Abstract

Mock modular forms, first invented by Ramanujan, provide a beautiful generalization of the usual modular forms. In recent years, it was found that they capture the generating functions of the number of microstates of BPS black holes appearing in compactifications of string theory with 8 and 16 supercharges. This review describes these results and their applications, which range from the actual computation of these generating functions for both compact and non-compact compactification manifolds (encoding, respectively, Donaldson–Thomas and Vafa–Witten topological invariants) to the construction of new non-commutative structures on moduli spaces of Calabi–Yau threefolds.

## 1. Introduction

One of the great achievements of string theory is the understanding of the nature of the microstates responsible for the black hole entropy. For various types of black holes appearing in string theory compactifications, this allowed to reproduce the celebrated Bekenstein–Hawking area law with the precise coefficient [1,2,3]. It is even more remarkable that, at least for some black holes preserving sufficient amount of supersymmetry, string theory is able to compute the number of black hole microstates *exactly* (see, e.g., [4,5,6,7,8,9])! The resulting integer numbers contain highly valuable information for quantum gravity because they should be obtainable by summing all quantum corrections, perturbative and non-perturbative, to the macroscopic Bekenstein–Hawking formula. Many quantum corrections have indeed been computed and matched against the exact microscopic counting (see [10] for a recent review) and, amazingly, for the simplest black holes, even the precise integer numbers have been recently reproduced [11], improving earlier results in [12,13].

In most cases where one has access to exact black hole degeneracies, this holds for a family of black holes labeled by a number of charges and what one really computes are their *generating functions*. For example, if one considers a family labeled by a single charge *n* bounded from below, it is natural to introduce a function

(1) $$h\left(\tau \right)=\sum_{n\geq n_{\min}}\Omega \left(n\right)q^{n},\;q=e^{2\pi i\tau },$$

where Ω(n) is the number of microstates for the black hole of charge *n*. A remarkable fact is that such generating functions typically turn out to be given by *modular forms* (see, e.g., [14]), i.e., they transform nicely (see Section 2.1 for the precise definition) under the following fractional linear transformation

(2) $$\tau \;\mapsto \;\frac{a\tau +b}{c\tau +d}\;,\;\left(\begin{array}{cc} a & b\\ c & d \end{array}\right)\in SL\left(2,Z\right).$$

Modular forms have been studied since the nineteenth century and are known to have very stringent properties. For example, the fact that for large charges Ω(n) behaves as an exponential of the black hole area can be seen as a simple consequence of the growth property satisfied by the Fourier coefficients of (weakly holomorphic) modular forms.

In string compactifications with many supercharges, it is actually quite natural to expect the appearance of modular forms. Indeed, such compactifications are typically constructed using a torus $T^{2}$ as a compact submanifold. The torus has a complex structure parametrized by a complex parameter τ living in the upper half-plane H, and SL(2,Z) is its modular group identifying tori with the complex structures related by (2). This means that all physical results depending on τ must be invariant under SL(2,Z). Of course, this does not imply yet the modularity of the generating function h(τ) because its argument is a formal expansion parameter and a priori has nothing to do with the complex structure of the torus. Nevertheless, in practice it does, and the observed modular behavior is not an accident.

If one reduces the number of supersymmetries, one encounters new interesting phenomena. First, in compactifications with 16 supercharges, such as type II string theory on $K3\times T^{2}$, the generating function of degeneracies of black holes preserving only four supercharges, known as $\frac{1}{4}$-BPS states, turns out to be the so-called Siegel modular form [4]. Such functions transform nicely under a large symmetry group Sp(2,Z). Despite the existence of some ideas in the literature [15], the origin of this extended symmetry remains rather mysterious.

In fact, this generating function hides another beautiful structure, which was revealed in [16]. To explain it, let us recall that the Fourier coefficients of our generating functions typically count *all* black holes of a given charge, including those which can be thought of as bound states. The latter are known as multi-centered black holes, in contrast to single-centered ones, and are full-fledged solutions of supergravity [17]. Of course, as any bound state, they are stable in some region of the parameter space, but can decay after crossing certain stability walls, which is known as the *wall-crossing* phenomenon. From this point of view, single-centered black holes are special since they never decay. In the context of N=4 supergravity in four dimensions, single-centered $\frac{1}{4}$-BPS black holes are called *immortal dyons* (because they must have both electric and magnetic charges, non-vanishing). Therefore, what was found in [16] is that the generating functions of degeneracies of the immortal dyons, which can be extracted from the Siegel modular form, are not modular, but *mock modular*! Since mock modularity will be the central topic of this review, let us briefly unveil what hides behind this notion. More details will be given in Section 2.2.

Mock modularity has its origin in the work of Srinivasa Ramanujan during the last year (1919–1920) of his life, which was found in his last letter to G.H. Hardy and in his famous lost notebook. There he put forward and analyzed several functions which he called *mock theta functions*. As the name suggests, he found that they are similar to ordinary theta functions, but not quite. Although it was clear that there was something special about these functions, one had to wait more than 80 years until their general theory was constructed by S. Zwegers [18] (see also [19]). According to this theory, mock theta functions are particular examples of *mock modular forms*. The latter are similar to modular forms, but different from them by failing to satisfy the modular transformation property. However, the anomaly, which measures how much they fail, has a special form being determined by another modular form called *shadow*. Equivalently, the form of the anomaly ensures that it can be canceled by adding a non-holomorphic term, also determined by the shadow, to the mock modular form, producing the so-called *modular completion*. Thus, a mock modular form is holomorphic but has a modular anomaly, while its completion is modular. but has a holomorphic anomaly.

Mock modular forms give rise to a natural and rich generalization of usual modular forms, which is still quite restrictive. In other words, if one knows that a function is mock modular with a given shadow, it is sufficient to find just a few data (for example, its first few Fourier coefficients) to fully determine the function. For the immortal dyons, this is not really a problem since their degeneracy can be calculated starting from the known Siegel form. However, in other cases, mock modularity provides an invaluable tool to find the objects of interest.

Keeping this in mind, let us further reduce supersymmetry and consider compactifications with eight supercharges, which are obtained by putting type II string theory on a Calabi–Yau (CY) threefold Y. In the type IIA formulation, supersymmetric black holes at the microscopic level are described as bound states of D6, D4, D2 and D0 branes wrapping non-contractible cycles of Y and characterized by an electro-magnetic charge $\gamma =(p^{0},p^{a},q_{a},q_{0})$ with $a=1,\ldots ,b_{2}\left(Y\right)$, where the components of the charge vector play the role of the respective brane charges. The BPS index, counting the number of states of these black holes, is known to coincide with the so-called generalized Donaldson–Thomas (DT) topological invariant of Y. In mathematical language, the bound state of D-branes corresponds to a complex of coherent sheaves on Y and the D6-brane charge $p^{0}$ is its rank.

Since there is no torus in the structure of a generic CY, one could think that the modular symmetry is not relevant for the above black holes. However, it turns out that it is, but only for a particular class corresponding to D4-D2-D0 bound states, i.e., with vanishing D6-brane charge. (As should be clear from above, for this class the black hole entropy is captured by *rank 0* DT invariants). The point is that in the dual M-theory picture, D4-D2-D0 BPS states, with D4-brane wrapped on a 4-cycle D, are realized by M5-brane wrapped on $D\times S^{1}$. In the limit of large $S^{1}$, the world-volume theory on the M5-brane reduces to a superconformal field theory (SCFT) in two dimensions, first considered in [3]. This theory allows us to define a modified elliptic genus [20], a torus partition function with certain insertions ensuring that only contributions of BPS states survive cancellations between bosons and fermions [21]. On one hand, it contains information about the BPS spectrum, i.e., the number of BPS states, and on the other hand, being defined on a torus, it is expected to be a modular form. This is why the generating functions of D4-D2-D0 BPS indices, or rank 0 DT invariants, are also expected to exhibit modular properties [7,22,23].

The precise modular properties of these generating functions have been derived only recently [24,25,26] using a different picture and turned out to be very intricate and beautiful. In fact, the main goal of this review is to explain these properties, their origin and the results produced on their basis. Before entering mathematical details, let us summarize here the main points for the ease of reading.

The main qualitative result is that the modular properties of the generating functions of D4-D2-D0 BPS indices with a fixed D4-brane charge $p^{a}$, which will be denoted by $h_{p,\mu }$ (the meaning of index μ will be explained in Section 4), crucially depend on this charge or, more precisely, on its *degree of reducibility*. It is equal to the maximal number of 4-cycles into which the wrapped cycle D can be decomposed, $D=\sum_{i=1}^{r}D_{i}$, where some $D_{i}$ may represent the same cycle. If the cycle D is irreducible, i.e., r=1, then the corresponding generating function is modular. If r=2, then it is mock modular. And if r>2, it is described by a generalization of mock modularity known as mock modular forms of higher depth.

Physically, the degree of reducibility can be thought of as the maximal number of constituents forming a bound state that can contribute to a given BPS index. This makes it clear why in compactifications with N=4 supersymmetry only usual mock modular forms appear, while in the N=2 story, one finds this intricate pattern: it is well known that in the former case there are only bound states with two constituents, whereas in the latter, any number of constituents is possible.

**Figure A1:**
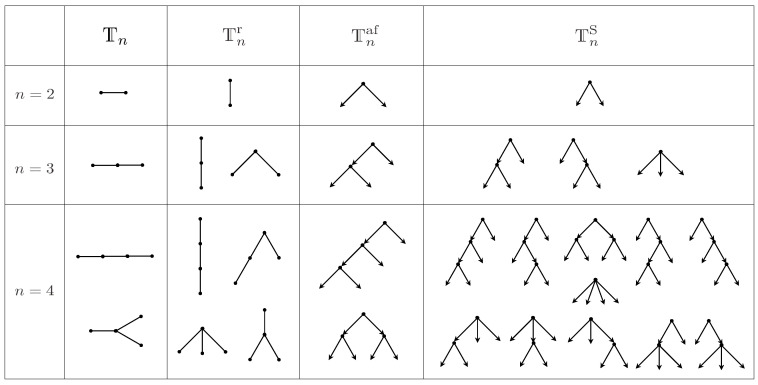
Various types of trees mentioned in the review. $T_{n}$ is the set of unrooted trees with *n* vertices. $T_{n}^{r}$ is the set of rooted trees with *n* vertices. $T_{n}^{\mathrm{af}}$ is the set of attractor flow trees with *n* leaves (of which we only draw the different topologies). $T_{n}^{S}$ is the set of Schröder trees with *n* leaves. In addition, an important role is played by the set $T_{n}^{\ell }$ of unrooted labeled trees, which are obtained from $T_{n}$ by assigning different labels to the vertices.

It is important that one knows not only the qualitative behavior of the generating functions under modular transformations, but also the precise form of their modular anomaly conveniently encoded in an explicit expression for their modular completion ${\hat{h}}_{p,\mu }$ [26,27]. It can be written as

(3) $${\hat{h}}_{p,\mu }\left(\tau ,\bar{\tau }\right)=h_{p,\mu }\left(\tau \right)+\sum_{n=2}^{r}\sum_{\sum_{i=1}^{n}p_{i}=p}\sum_{\left\{\mu_{i}\right\}}R_{\mu ,\{\mu_{i}\}}^{\left(\left\{p_{i}\right\}\right)}\left(\tau ,\bar{\tau }\right)\prod_{i=1}^{n}h_{p_{i},\mu_{i}}\left(\tau \right),$$

where the second sum goes over all ordered decompositions of the D4-brane charge into charges with non-negative components and can be thought of as a sum over possible bound states. The main non-trivial ingredient of this formula is the function $R_{\mu ,\{\mu_{i}\}}^{\left(\left\{p_{i}\right\}\right)}$ which will be defined in Section 5. Here we just mention the fact that it is given by a sum over various types of trees, which was used in the title to intrigue the reader (see Figure A1 for an illustration of relevant trees).

Since the r.h.s. of (3) depends on the generating functions for smaller charges corresponding to constituents, the set of equations for different D4-brane charges gives rise to an iterative system of anomaly equations on the functions $h_{p,\mu }$. This system has a very rich and universal structure because, although it was originally derived in a concrete setup (compactification on a compact CY with D4-brane wrapping an ample divisor), it turns out that it is applicable or has a simple extension to much more general situations. For instance, it is still valid for certain degenerations and, in particular, for non-compact CY manifolds [28]. Since non-compact CYs can be used to geometrically engineer gauge theories, in favorable circumstances, their BPS spectrum can also be constrained by our anomaly equations. Thus, in [29,30], they have been used to solve Vafa–Witten (VW) topological theory with gauge group U(r) on various rational surfaces, for arbitrary rank *r*. Furthermore, the system of the anomaly equations (3) has a natural generalization that includes the so-called *refinement* [28], a one-parameter deformation corresponding physically to switching on the Ω-background [31,32]. In turn, this generalization allows us to put the compactifications with higher supersymmetry with different numbers of preserved supercharges into the same single framework, so that most of the known modularity results on the generating functions of BPS indices, including the mock modularity of the immortal dyons [16], turn out to be consequences of this extended system [33].

Finally, and perhaps most importantly, the constraints of modularity can be used as a tool to find explicit expressions for the generating functions $h_{p,\mu }$ and thereby to determine the exact degeneracies of BPS black holes. In fact, this program was initiated long ago in [7], where it was applied to the generating function $h_{1,\mu }$ of D4-D2-D0 BPS indices with unit D4-brane charge on the quintic threefold, and then extended to a few other one-parameter CYs in [34,35,36]. In this case, there is no modular anomaly yet since the generating function must be a modular form, and the main difficulty consists in computing its polar terms, given by the Fourier coefficients in (1) with negative *n*, which together with modularity are enough to determine the full function. However, the two approaches used in that works are hardly generalizable and even produced mutually inconsistent results. A more systematic approach has been proposed recently in [37] and allowed to resolve the previous inconsistencies and to compute $h_{1,\mu }$ for a dozen one-parameter CY threefolds [37,38].

The modular anomaly starts playing a crucial role when one goes to higher D4-brane charges. Then the polar terms are not enough to uniquely fix the generating functions and one should follow a two-step procedure: (i) first, solve the modular anomaly, which gives a unique solution up to the addition of a pure modular form (modular ambiguity); (ii) fix the ambiguity by computing the polar terms. For one-parameter CY threefolds, the first step has been realized for r=2 in [39] and for arbitrary *r* in [40]. The second step has been done so far only for two CY threefolds and for a D4-brane charge equal to 2 [41]. As a result, for the first time, we got access to charge 2 states on CY threefolds without any additional structure that are organized in a mock modular form. Furthermore, the computation of Fourier coefficients beyond polar terms following the approach of [37] provided an impressive test of (mock) modularity, which still remains conjectural from the mathematical point of view (see [42] for an attempt to rigorously prove it in the simplest pure modular case on the quintic threefold).

Finally, one should mention another important implication of the above results (see Section 8.1 for more details). The method of [37] to compute polar terms relies on the knowledge of Gopakumar–Vafa (GV) invariants, which can be mapped to rank 0 DT invariants using the so-called MNOP formula [43,44] and wall-crossing relations. The GV invariants, in turn, are computed using the direct integration approach to topological string theory on compact threefolds, which is based on solving a holomorphic anomaly equation for its partition function [45,46,47]. The problem, however, is that the solution has a holomorphic ambiguity that needs to be fixed, precisely as our modular anomaly equations fix the generating functions only up to a modular ambiguity. Fortunately, there are several well-known conditions that can be used for this purpose. But their number grows more slowly with genus than the number of parameters to be fixed, so that at some maximal genus the method does not work anymore. However, once a generating function of rank 0 DT invariants is found, one can invert the relations mentioned above and find new GV invariants that can serve as new conditions for fixing the holomorphic ambiguity. Thus, the two systems of anomaly equations work together and help each other to overcome their own limitations.

The organization of the review is as follows. In the next two sections, we provide the mathematical background needed to understand the results presented below. First, in Section 2, we introduce modular and mock modular forms. Then, in Section 3, we describe an important class of functions known as indefinite theta series, which provide the simplest example of mock modular forms and play an important role in our construction. In Section 4, we define the main object of interest—generating functions of D4-D2-D0 BPS indices. In Section 5, we present the main result about their modular behavior, while in Section 6, we discuss its various extensions, including some degenerate cases, non-compact CYs, and the refinement. In Section 7, we explain a solution to the system of anomaly equations, and in Section 8, we present various applications, including the computation of topological invariants on compact CY threefolds, the solution of Vafa–Witten theory and an extension to higher supersymmetry. We conclude with a discussion of open issues in Section 9.

## 2. Modular and Mock Modular Forms

### 2.1. Modular Forms

Let us first review the definition of a standard modular form and then incorporate various (also standard) generalizations that are required in physical applications. For a more in-depth presentation, one can consult [48,49].

In this review we deal only with modular forms of SL(2,Z) represented by 2×2 matrices as in (2) with integer coefficients and a determinant equal to 1. We denote the modular parameter by $\tau =\tau_{1}+i\tau_{2}$ and take it to be a complex number belonging to the upper half-plane H defined by the condition $\tau_{2}>0$. Then one has

**Definition** **1.** *A function h(τ) is a modular form of weight w if it is holomorphic on H, bounded as $\tau_{2}\to \infty$ and transforms as*

(4) $$h\left(\frac{a\tau +b}{c\tau +d}\right)={\left(c\tau +d\right)}^{w}h\left(\tau \right)\;.$$

It immediately follows that h(τ) is periodic under τ↦τ+1 and hence has the following Fourier expansion

(5) $$h\left(\tau \right)=\sum_{n=0}^{\infty }h_{n}q^{n},\;q=e^{2\pi i\tau }.$$

**Example** **1.** *For integer k>1, the Eisenstein series*

(6) $$E_{2k}\left(\tau \right)=\frac{1}{2\zeta (2k)}\sum_{\left(m,n\right)\in Z^{2}\setminus \left\{\left(0,0\right)\right\}}\frac{1}{{\left(m\tau +n\right)}^{2k}}$$

*is a modular form of weight 2k. The overall coefficient given by the Riemann zeta function is introduced so that the constant term of the Fourier series is equal to 1.*

In fact, the Definition 1 is so restrictive that all such modular forms can be generated by just two Eisenstein series, $E_{4}$ and $E_{6}$. Namely, each modular form of weight *w* has a unique expansion as

(7) h(τ)=∑4k+6l=wk,l≥0ck,lE4k(τ)E6l(τ).

Therefore, it is natural to relax the definition in several ways.

First, we relax the behavior at infinity and instead require *h* to have at most polynomial growth in $q^{-1}$ as $\tau_{2}\to \infty$. Provided *h* still satisfies (4), it is called *weakly holomorphic* modular form and, if $h\left(\tau \right)=O\left(q^{-N}\right)$, its Fourier expansion (5) acquires additional terms with −N≤n<0.

**Example** **2.** *The inverse discriminant function $\Delta^{-1}\left(\tau \right)$, where $\Delta =(E_{4}^{3}-E_{6}^{2})/1728$, is a weakly holomorphic modular form of weight −12. Its Fourier expansion starts with $\Delta^{-1}\left(\tau \right)=q^{-1}+24+\cdots$.*

Second, one can relax the transformation property (4) by allowing a phase factor depending on the group element, which is called *multiplier system* of the modular form. The modified modular transformation is then given by

(8) $$h\left(\frac{a\tau +b}{c\tau +d}\right)={\left(c\tau +d\right)}^{w}M\left(\rho \right)h\left(\tau \right)\;,\;\rho =\left(\begin{array}{cc} a & b\\ c & d \end{array}\right)\in SL\left(2,Z\right).$$

It is clear that M(ρ) should satisfy a cocycle condition, which makes it similar to a character of SL(2,Z). Moreover, since SL(2,Z) is generated by two elements,

(9) $$T=\left(\begin{array}{cc} 1 & 1\\ 0 & 1 \end{array}\right)\;\mathrm{and}\;S=\left(\begin{array}{cc} 0 & -1\\ 1 & 0 \end{array}\right),$$

the multiplier system is completely characterized by specifying M(T) and M(S). The advantage of allowing for a multiplier system is that now the Fourier expansion of a modular form does not need to be in integer powers of q and the weight *w* can be half-integer.

**Example** **3.** *The Dedekind eta function*

(10) $$\eta \left(\tau \right)=\Delta^{\frac{1}{24}}\left(\tau \right)=q^{\frac{1}{24}}\prod_{n=1}^{\infty }\left(1-q^{n}\right)=q^{\frac{1}{24}}\sum_{n\in Z}{\left(-1\right)}^{n}q^{\frac{1}{2}\left(3n^{2}-n\right)}$$

*is a modular form of weight 1/2 and multiplier system*

(11) $$M^{\left(\eta \right)}\left(T\right)=e^{\frac{\pi i}{12}},\;M^{\left(\eta \right)}\left(S\right)=e^{-\frac{\pi i}{4}}.$$

Another important generalization is to *vector-valued modular forms*. In this case, instead of a single function h(τ), one considers a vector of functions $h_{\mu }\left(\tau \right)$ where the index μ takes a finite number of values. Then the only modification to be done in the above equations is that the multiplier system becomes matrix valued, $M_{\mu \nu }\left(\rho \right)$, so that the modular transformation reads

(12) $$h_{\mu }\left(\frac{a\tau +b}{c\tau +d}\right)={\left(c\tau +d\right)}^{w}\sum_{\nu }M_{\mu \nu }\left(\rho \right)h_{\nu }\left(\tau \right)\;.$$

Furthermore, the Fourier expansion now has the following form

(13) $$h_{\mu }\left(\tau \right)=\sum_{n\in N-\Delta_{\mu }}h_{\mu ,n}q^{n},$$

where $\Delta_{\mu }$ can be rational numbers.

**Example** **4.** *The two-component vector*

(14) $$\vartheta_{\mu }\left(\tau \right)=\left(\theta_{3}\left(2\tau \right),\theta_{2}\left(2\tau \right)\right)=\sum_{n\in Z+\mu /2}q^{n^{2}},\;\mu =0,1,$$

*where $\theta_{2}$ and $\theta_{3}$ are the standard Jacobi theta functions, is a vector-valued modular form of weight 1/2 and multiplier system*

(15) $$M_{\mu \nu }^{\left(\vartheta \right)}\left(T\right)=e^{\frac{\pi i}{2}\;\mu^{2}}\delta_{\mu \nu },\;M_{\mu \nu }^{\left(\vartheta \right)}\left(S\right)=\frac{e^{-\frac{\pi i}{4}}}{\sqrt{2}}\;{\left(-1\right)}^{\mu \nu }.$$

Finally, one can drop the holomorphicity condition and define modular forms of mixed weight $\left(w,\bar{w}\right)$. Thus, in the most general case, we have the following

**Definition** **2.** *$h(\tau ,\bar{\tau })$ is a vector valued modular form of weight $\left(w,\bar{w}\right)$ and multiplier system $M_{\mu \nu }$ if it satisfies*

(16) $$h_{\mu }\left(\frac{a\tau +b}{c\tau +d}\;,\;\frac{a\bar{\tau }+b}{c\bar{\tau }+d}\right)={\left(c\tau +d\right)}^{w}{\left(c\bar{\tau }+d\right)}^{\bar{w}}\sum_{\nu }M_{\mu \nu }\left(\rho \right)h_{\nu }\left(\tau ,\bar{\tau }\right)\;.$$

**Example** **5.** *A trivial example of a non-holomorphic modular form is given by $\tau_{2}$, which has weight (−1,−1).*

It is important that as long as one remains in the realm of (weakly) holomorphic modular forms, for a given weight and multiplier system, the transformation property (12) restricts them to form a finite-dimensional space. In particular, for weakly holomorphic modular forms of negative weight the dimension of this space is bounded from above by the number of *polar terms*, i.e., the terms in the Fourier expansion in (13) with negative power *n* [50,51,52]. In the vector-valued case, one should sum up the number of polar terms for all (independent) components.

### 2.2. Mock Modular Forms

The next level of generalization is provided by (vector valued weakly) holomorphic *mock modular forms*, which spoil the transformation property (12), but in a very specific way controlled by another modular form. More precisely, if $h_{\mu }$ is mock modular of weight *w*, it should satisfy

(17) $$h_{\mu }\left(\frac{a\tau +b}{c\tau +d}\right)={\left(c\tau +d\right)}^{w}\sum_{\nu }M_{\mu \nu }\left(\rho \right)\left(h_{\nu }\left(\tau \right)-\int_{-d/c}^{-i\infty }\frac{\bar{g_{\nu }\left(\bar{z}\right)}}{{\left(\tau -z\right)}^{w}}\;dz\right),$$

where $g_{\mu }$ is a modular form of weight 2−w, called the *shadow* of $h_{\mu }$. It is easy to see that the anomalous term can be canceled by adding to $h_{\mu }$ a *non-holomorphic* contribution, known as the Eichler or period integral of the shadow,

(18) $$g_{\mu }^{*}\left(\tau ,\bar{\tau }\right)=\int_{\bar{\tau }}^{-i\infty }\frac{\bar{g_{\mu }\left(\bar{z}\right)}}{{\left(\tau -z\right)}^{w}}\;dz.$$

The resulting non-holomorphic function

(19) $${\hat{h}}_{\mu }\left(\tau ,\bar{\tau }\right)=h_{\mu }\left(\tau \right)-g_{\mu }^{*}\left(\tau ,\bar{\tau }\right)$$

is called the *modular completion* of $h_{\mu }$ and transforms as a usual modular form of weight (w,0). It contains all interesting information: on one hand, the original mock modular form can be obtained from it by taking the limit $\bar{\tau }\to \infty$ keeping τ fixed, and on the other hand, the shadow can be extracted by taking the non-holomorphic derivative

(20) $$g_{\mu }\left(\tau \right)=\bar{{\left(2i\tau_{2}\right)}^{w}\partial_{\bar{\tau }}{\hat{h}}_{\mu }}.$$

In fact, the completion provides an alternative and somewhat more convenient way of defining mock modular forms.

**Definition** **3.** *A (vector valued weakly) holomorphic function $h_{\mu }$ is a mock modular form of weight w with shadow $h_{\mu }$, if its completion defined by (19) transforms as a usual modular form of the same weight.*

**Example** **6.** *The simplest example of a mock modular form is provided by the quasi-modular Eisenstein series $E_{2}\left(\tau \right)$. It can be defined by the same Formula (6) as other Eisenstein series, specified to k=1, but in contrast to them, the double sum is not absolutely convergent, which is the origin of a modular anomaly. Alternatively, it can be expressed as a logarithmic derivative of the discriminant function Δ(τ)*

(21) $$E_{2}\left(\tau \right)=\frac{1}{2\pi i}\;\partial_{\tau }\log\Delta \left(\tau \right)=1-24\sum_{n=1}^{\infty }\frac{nq^{n}}{1-q^{n}}\;.$$

*From the modular transformation of Δ(τ), it is immediate to derive the transformation of $E_{2}\left(\tau \right)$:*

(22) $$E_{2}\left(\frac{a\tau +b}{c\tau +d}\right)={\left(c\tau +d\right)}^{2}\left(E_{2}\left(\tau \right)+\frac{6}{\pi i}\;\frac{c}{c\tau +d}\right),$$

*which fits the transformation of a generic mock modular form with the shadow taken to be constant, g=6i/π. It is also easy to check that the following non-holomorphic function*

(23) $${\hat{E}}_{2}\left(\tau ,\bar{\tau }\right)=E_{2}\left(\tau \right)-\frac{3}{\pi \tau_{2}}$$

*transforms as a standard modular form and fits the definition of the completion (19) with the same shadow.*

**Example** **7.** *The n-th Hurwitz class number H(n) is defined as the number of PSL(2,Z)-equivalence classes of integral binary quadratic forms of discriminant n, divided by the number of their automorphisms. Setting also H(0)=−1/12, they can be organized into a generating series. However, it does not transform properly under the full SL(2,Z) group. Therefore, it is more convenient to split it into a two-component vector*

(24) $$\begin{array}{cc} H_{0}\left(\tau \right)= & \;\sum_{n\geq 0}H\left(4n\right)q^{n}=-\frac{1}{12}+\frac{1}{2}\;q+q^{2}+\frac{4}{3}\;q^{3}+\frac{3}{2}\;q^{4}+2q^{5}+\ldots \;,\\ H_{1}\left(\tau \right)= & \;\sum_{n>0}H\left(4n-1\right)q^{n}=q^{\frac{3}{4}}\left(\frac{1}{3}+q+q^{2}+2q^{3}+q^{4}+3q^{5}+\ldots \right), \end{array}$$

*where H(4n+1) and H(4n+2) do not appear because they all vanish. It has been discovered in [53] that $H_{\mu }$ is a vector valued mock modular form of weight 3/2 with the shadow proportional to the theta series $\vartheta_{\mu }$ (14). This example is highly important because this function, after multiplication by 3, turns out to coincide with the generating series of SU(2) Vafa–Witten invariants on $P^{2}$ [54], which is one of the first examples of the appearance of mock modularity in physics.*

Although mock modular forms are much more general than modular forms, they are still severely restricted by their transformation property. For example, a weakly holomorphic mock modular form of negative weight is completely determined by its polar terms, similarly to its pure modular cousin. The difference is that, in general, the standard modularity requires the polar coefficients to satisfy certain constraints, whereas they can be chosen freely to generate a mock modular form [51]. In this sense, mock modular forms are even “more natural” than usual ones.

Before we move on, we need to introduce one more generalization.

**Definition** **4.** *$h_{\mu }$ is a mixed mock modular form of weight w if its modular completion has the form*

(25) $${\hat{h}}_{\mu }=h_{\mu }-\sum_{j,\alpha }f_{j,\mu \alpha }\;g_{j,\alpha }^{*},$$

*where $f_{j,\mu \alpha }$ and $g_{j,\alpha }$ are holomorphic modular forms of weight $w+r_{j}$ and $2+r_{j}$, respectively.*

In other words, for mixed mock modular forms, the shadow is allowed to be a sum of products of holomorphic and anti-holomorphic functions. It is clear that one can generate infinitely many examples of such functions by simply taking products of mock modular forms with usual modular forms. In fact, most of the mock modular forms appearing in this review will be of the mixed type.

Note also that for this class of functions, the knowledge of only polar terms is not sufficient anymore to fix them uniquely. As we will see, in addition, one should know the precise modular anomaly encoded in the shadow.

### 2.3. Higher Depth Mock Modular Forms

Mixed mock modular forms are at the basis of another huge generalization, which is known as *higher depth mock modular forms* [55]. This class of functions is defined iteratively in depth. Namely, we take the usual modular and mock modular forms as objects of depth 0 and 1, respectively. Then we take

**Definition** **5.** *$h_{\mu }$ is a depth r mock modular form of weight w if the anti-holomorphic derivative of its modular completion has the form*

(26) $$\partial_{\bar{\tau }}{\hat{h}}_{\mu }^{\left(r\right)}=\sum_{j,\alpha }\tau_{2}^{r_{j}}\;{\hat{h}}_{j,\mu \alpha }^{\left(r-1\right)}\;\bar{g_{j,\alpha }},$$

*where $g_{j,\alpha }$ are modular forms of weight $2+r_{j}$, while ${\hat{h}}_{j,\mu \alpha }^{\left(r-1\right)}$ are completions of mock modular forms of depth r−1 and weight $w+r_{j}$.*

We will provide an important example of higher depth mock modular forms in Section 3. Furthermore, as already mentioned in the Introduction, the generating functions of D4-D2-D0 BPS indices in CY string compactifications, coinciding with rank 0 DT invariants, also turn out to belong to this class. Thus, the higher depth modularity is not an abstract generalization, but captures the modular properties of important physical and mathematical objects.

### 2.4. Jacobi Forms and Their Variations

We finish our presentation of modular functions by introducing the so-called *Jacobi forms*, which carry dependence on an additional complex variable *z* [56]. Besides the modular weight *w*, their transformation properties are characterized also by a number *m* known as *index*. Their precise definition is as follows:

**Definition** **6.** *A holomorphic function φ(τ,z) on H×C is a Jacobi form of weight w and index m if it satisfies*

(27a) $$\begin{array}{ccc} \varphi (\tau ,z+a\tau +b) & = & e^{-2\pi im\left(a^{2}\tau +2az\right)}\;\varphi \left(\tau ,z\right),\;a,b\in Z, \end{array}$$

(27b) $$\begin{array}{ccc} \varphi \left(\frac{a\tau +b}{c\tau +d},\frac{z}{c\tau +d}\right) & = & {\left(c\tau +d\right)}^{w}\;e^{\frac{2\pi imcz^{2}}{c\tau +d}}\varphi \left(\tau ,z\right). \end{array}$$

Of course, setting z=0, any Jacobi form gives rise to a modular form. However, even for non-vanishing *z*, Jacobi forms are, in a sense, constructed out of modular forms. Indeed, using the “elliptic property” (27a), it is easy to show that φ(τ,z) has the following theta expansion

(28) $$\varphi \left(\tau ,z\right)=\sum_{\mu =0}^{2m-1}h_{\mu }\left(\tau \right)\theta_{\mu }^{\left(m\right)}\left(\tau ,z\right),$$

where

(29) $$\theta_{\mu }^{\left(m\right)}\left(\tau ,z\right)=\sum_{k\in 2mZ+\mu }q^{\frac{k^{2}}{4m}}\;y^{k},\;y=e^{2\pi iz},$$

is a unary theta series, while $h_{\mu }\left(\tau \right)$ is a vector valued modular form of weight w−1/2. Thus, Jacobi forms carry essentially the same information as vector-valued modular forms, but allow to encode it in a more compact and nice way.

The Jacobi forms introduced above are an extension of the modular forms from Definition 1. Similarly to the discussion in Section 2.1, one can upgrade them to be vector valued, have a non-trivial multiplier system and carry a non-holomorphic dependence. All these generalizations are obvious, so we do not provide the corresponding transformation properties.

Furthermore, one can also allow for multiple elliptic parameters $z_{i}$ so that φ(τ,z) becomes a *multi-variable* Jacobi form with $z=(z_{1},\ldots z_{n})$. In this situation, $z^{2}$ appearing in the exponential in (27b) should be replaced by $z^{2}=\sum_{i,j=1}^{n}Q_{ij}z_{i}z_{j}$ where $Q_{ij}$ defines a scalar product in $C^{n}$. In addition, the index now becomes matrix valued and equal to $mQ_{ij}$. Such multi-variable Jacobi forms still have a theta expansion, but the unary theta series (29) is replaced by a theta series defined on a $Z^{n}$ lattice with quadratic form $2mQ_{ij}$.

**Example** **8.** *The Jacobi theta function*

(30) $$\theta_{1}\left(\tau ,z\right)=\sum_{k\in Z+\frac{1}{2}}q^{k^{2}/2}{\left(-y\right)}^{k}$$

*is a Jacobi form of weight 1/2, index 1/2 and the following multiplier system*

(31) $$M^{\left(\theta_{1}\right)}\left(T\right)=e^{\frac{\pi i}{4}},\;M^{\left(\theta_{1}\right)}\left(S\right)=e^{-\frac{3\pi i}{4}}.$$

Given the existence of the theta expansion, it is straightforward to introduce *mock Jacobi* and even *higher depth mock Jacobi forms*. They can be defined as functions having a theta expansion (28) where $h_{\mu }$ is a mock or higher depth mock modular form. This also allows us to talk about modular completions of mock Jacobi forms $\hat{\varphi }$ given by

(32) $$\hat{\varphi }\left(\tau ,\bar{\tau },z\right)=\sum_{\mu =0}^{2m-1}{\hat{h}}_{\mu }\left(\tau ,\bar{\tau }\right)\theta_{\mu }^{\left(m\right)}\left(\tau ,z\right).$$

Finally, if one drops the elliptic property (27a), but keeps the modular transformation (27b), one arrives at the definition of *Jacobi-like forms*. They are not periodic in *z*, so that they do not need to depend on it through the exponential *y* as in (29). As a result, they do not have a theta expansion and hence cannot be reduced to a single modular form. Instead, they give rise to an infinite set of modular forms through a Laurent expansion in *z* [57,58]. One way to construct them is provided by the following proposition [40]

**Proposition** **1.** *Let $\varphi_{\mu }\left(\tau ,z\right)$ be a Jacobi-like form of modular weight w and index m, and having a smooth limit at z→0. We define the following differential operator*

(33) $$D_{m}^{\left(n\right)}=\sum_{k=0}^{\left\lfloor n/2\right\rfloor }c_{n,k}E_{2}^{k}\left(\tau \right)\;\partial_{z}^{n-2k},\;c_{n,k}=\frac{n!{\left(\frac{2m}{3}\pi^{2}\right)}^{k}}{(2k)!!(n-2k)!}\;.$$

*Then*

(34) $$\phi_{\mu }^{\left(n\right)}\left(\tau \right)\equiv D_{m}^{\left(n\right)}\varphi_{\mu }{\left(\tau ,z\right)|}_{z=0}$$

*are vector-valued modular forms of weight w+n.*

The differential operators $D_{m}^{\left(n\right)}$ and the modular forms generated by them will appear in Section 7 in the construction of a solution of the modular anomaly equation that governs the generating functions of black hole degeneracies in CY compactifications.

## 3. Indefinite Theta Series

In this section, we recall a few facts about an important class of functions having interesting modular properties, which are known as (generalized) *theta series*. Usually, such a theta series is associated with a lattice 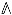 endowed with an integer valued quadratic form $k^{2}=k★k$ and can be schematically written as

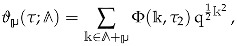
(35)
where 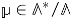 is the so called *residue class* valued in the discriminant group of the lattice and Φ is a function of at-most polynomial growth, which we will call a *kernel*.

In the simplest case, the quadratic form is positive definite, so that the sum in (35) is absolutely convergent for τ∈H. If in addition Φ=1, the theta series is known to be a modular form of weight d/2 where 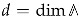. (Strictly speaking, this is true only for even lattices for which the quadratic form takes values in 2Z. For odd lattices, Φ should be chosen to be a sign factor to produce a modular form). Example 4 provides the simplest illustration of this situation. By inserting the factor $e^{2\pi ik★z}$ depending on a vector of elliptic parameters z, one can also convert the theta series into a (multi-variable) Jacobi form (see Example 8). This shows that, with a properly chosen kernel, the theta series satisfies standard modular transformation properties of type (12) or (27).

The situation drastically changes once one allows the quadratic form to have an indefinite signature, say (d−n,n) with n≥1. An immediate problem is that, for generic Φ, the sum in (35) becomes divergent. Thus, the kernel *must* be non-trivial just to have a well-defined theta series.

There are basically two ways to achieve the convergence. The simplest way is to choose the kernel to be exponentially decaying in the “dangerous” directions of the lattice where the quadratic form is negative definite. However, since the kernel is allowed to depend only on $\tau_{2}$, not on τ, this can be done only at the cost of introducing non-holomorphicity. For example, one can take $\Phi =e^{2\pi \tau_{2}k_{-}^{2}}$ where $k_{\pm }$ denote the projections of k, respectively, on the positive and negative definite sublattices. The resulting 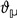 is the Siegel theta series

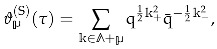
(36)
which is easily seen to be a non-holomorphic modular form of weight $\left(\frac{d-n}{2},\frac{n}{2}\right)$.

However, often one has to deal with a *holomorphic* indefinite theta series, which can be obtained by the second method of achieving convergence. In this case, one takes the kernel so that it simply vanishes in the dangerous directions. Such behavior is easy to get if Φ is a combination of sign functions. The simplest construction of this type is provided by the following theorem from [29] (generalizing results of [59,60,61]):

**Theorem** **1.** *Let the signature of the quadratic form be (d−n,n) and*

(37) $$\Phi \left(k\right)=\prod_{i=1}^{n}\left(sgn\left(v_{1,i}★k\right)-sgn\left(v_{2,i}★k\right)\right),$$

*where $\left\{v_{1,i}\right\}$, $\left\{v_{2,i}\right\}$ are two sets of d-dimensional vectors. Then, the theta series (35) is convergent provided:*

*1.*  *for all $i\in Z_{n}=\left\{1,\ldots ,n\right\}$, $v_{1,i}^{2},v_{2,i}^{2}<0$;*
*2.*  *for any subset $I\subseteq Z_{n}$ and any set of $s_{i}\in \left\{1,2\right\}$, i∈I,*
(38) $$\underset{i,j\in I}{\;\det\;}\left(-v_{s_{i},i}★v_{s_{j},j}\right)\geq 0;$$
*3.*  *for all $\ell \in Z_{n}$ and any set of $s_{i}\in \left\{1,2\right\}$, $i\in Z_{n}\setminus \left\{\ell \right\}$,*
(39) $$v_{1,\ell ⊥\{s_{i}\}}★v_{2,\ell ⊥\{s_{i}\}}<0,$$
  *where _*⊥{*s_i_*}*_ denotes the projection on the subspace orthogonal to the span of ${\left\{v_{s_{i},i}\right\}}_{i\in Z_{n}\setminus \left\{\ell \right\}}$.*

**Remark** **1.** *We warn the reader that in most of the above cited references, one uses a convention where there is a minus sign in the power of q in (35). This corresponds to flipping the overall sign of the quadratic form and hence all inequalities in Theorem 1 should be inverted.*

Thus, to get a convergent holomorphic theta series, it is sufficient to take the kernel to be a product of differences of the sign functions determined by a set of vectors of negative norm, subject to a few conditions on their scalar products. The number of factors should be equal to the number of negative eigenvalues of the quadratic form. Importantly, if one considers a theta series including an elliptic parameter (or a vector thereof), one gets convergence even if some of the vectors $v_{s,i}$ are null, i.e., satisfy $v_{s,i}^{2}=0$, provided they belong (possibly after a rescaling) to the lattice 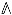.

Of course, the kernel (37) is not the only possibility to get a convergent indefinite theta series. For example, for n=2, in [60], an interesting cyclic combination of sign functions has been conjectured also to ensure the convergence. The conjecture was given a geometric interpretation and proven and extended in [62,63].

An important consequence of having a non-trivial kernel is that it spoils modularity. The theta series with a kernel constructed from sign functions transforms under SL(2,Z) with a modular anomaly. A remarkable fact is that any such theta series turns out to belong to the class of higher depth mock modular forms, with the depth equal to *n*. In particular, for n=1 corresponding to the case of indefinite theta series of Lorentzian signature, one obtains (mixed) mock modular forms, many of which are related to classic examples going back to Ramanujan.

As was explained in Section 2.2, each mock modular form has a non-holomorphic modular completion. Therefore, a natural and important question is: what are the completions of indefinite theta series? For the case of the Lorentzian signature, the answer has been found in [18] and is extremely simple: the completion is given by a theta series with the kernel obtained by replacing each sign function in (37) by the error function according to

(40) $$\mathrm{sgn}\left(v★k\right)\;\mapsto \mathrm{Erf}\left(\sqrt{2\pi \tau_{2}}\;\frac{v★k}{\left||v|\right|}\right),$$

where $\left||v|\right|=\sqrt{-v^{2}}$. It turns out that for n>1 the recipe is very similar and can be formulated in terms of the so-called generalized error functions described in Appendix B. They can be seen as functions $\Phi_{n}^{E}\left(\left\{v_{i}\right\};x\right)$ of a vector x and a set of *n* vectors $v_{i}$, which are given by a convolution of $\prod_{i=1}^{n}\mathrm{sgn}\left(v_{i}★\;x\right)$ with a Gaussian kernel. Then the recipe says [64]:

*To construct the completion of a theta series whose kernel is a combination of sign functions, it is sufficient to replace each product of the sign functions according to the rule*

(41) $$\prod_{i=1}^{n}sgn\left(v_{i}★\;k\right)\;\mapsto \Phi_{n}^{E}\left(\left\{v_{i}\right\};\sqrt{2\tau_{2}}k\right).$$

Although it is easy to show a posteriori that the recipe (41) does produce a non-holomorphic modular form, to guess the functions $\Phi_{n}^{E}\left(\left\{v_{i}\right\};x\right)$ could have been an outstanding problem. Fortunately, the guesswork was not required as string theory produced them for free! The point is that such holomorphic indefinite theta series often arise in the analysis of Calabi–Yau compactifications because, as will be explained in Section 4, the lattice $\Lambda =H_{4}\left(Y,Z\right)$ has signature $\left(1,b_{2}\left(Y\right)-1\right)$. However, typically in physics, modular symmetry is more fundamental than holomorphicity (see, e.g., [65]). Therefore, the final physical results should be expressible in terms of (possibly non-holomorphic) modular forms rather than holomorphic mock modular forms. In particular, this implies that string theory should “know” about the non-holomorphic modular completions of indefinite theta series and they can be found by a (not necessarily simple) calculation. This is precisely what was done in [60] and led to the introduction of the generalized error functions in [59,64] and to the above construction (see also [66]).

We finish this section by presenting a very general result on modularity of indefinite theta series, which is a straightforward generalization of a Theorem proven by Vignéras in [67]. As above, we take 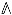 to be a *d*-dimensional lattice equipped with an integer-valued bilinear form. But from this moment, to agree with most of the relevant literature (see Remark 1), we change conventions and take the bilinear form to be *opposite* to the one considered before. Hence, the associated quadratic form is assumed to have signature (n,d−n) so that the case of convergent theta series with trivial kernel corresponds to a negative definite quadratic form. To avoid confusion with the previous conventions, we use the symbol ∗ instead of ★ for the bilinear form. In addition, we take 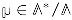 to be a residue class and p a characteristic vector satisfying k∗(k+p)=0mod2 for 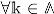, which allows to deal with odd lattices. Finally, 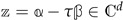 with 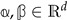 will be a vector of elliptic parameters. Using these notations, we define

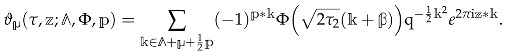
(42)
The Vignéras theorem [67] asserts that if the kernel Φ(x) satisfies suitable decay properties as well as the following differential equation

(43) $$\left[\partial_{x}^{2}+2\pi \left(x*\partial_{x}-\lambda \right)\right]\Phi \left(x\right)=0,$$

where λ is an integer parameter, then the theta series is a vector-valued (multi-variable) Jacobi form with the following weight, index and multiplier system

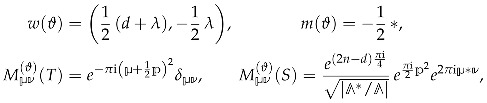
(44)
where by ∗ in the formula for the index, we mean the matrix representing the bilinear form. (More precisely, the elliptic transformation (27a) can generate an additional sign factor ${\left(-1\right)}^{p*(a+b)}$). If one takes 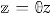 where 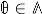, so that the multi-variable Jacobi form is reduced to a usual Jacobi form, the index is a scalar and is given by 
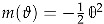
.

## 4. BPS Indices and Their Generating Functions

### 4.1. BPS Indices in Type IIA/CY

Let us now turn to physics and consider type IIA string theory compactified on a CY threefold Y. In four non-compact dimensions, one gets an effective theory given by N=2 supergravity coupled to $b_{2}=h^{1,1}\left(Y\right)$ vector multiplets and $h^{2,1}\left(Y\right)+1$ hypermultiplets. The theory has $b_{2}+1$ abelian gauge fields, which include the graviphoton belonging to the gravitational multiplet.

BPS states are labeled by an electro-magnetic charge, which can be represented as a vector with $2b_{2}+2$ components and is denoted by $\gamma =(p^{0},p^{a},q_{a},q_{0})$, where $a=1,\ldots ,b_{2}$ labels vector multiplets. At strong string coupling, these BPS states appear as black hole solutions of the effective theory, while at small string coupling they are realized as bound states of D6, D4, D2 and D0 branes wrapping 6, 4, 2 and 0-dimensional cycles of Y, respectively. The components of the charge vector correspond to the respective D-brane charges. Note that the magnetic charges $p^{0}$ and $p^{a}$ are always integers, whereas the electric charges are in general rational due to a non-trivial quantization condition [68].

The BPS states are counted (with sign) by a *BPS index* Ω(γ), which is stable under deformations of the string coupling and other hypermultiplet moduli. This is the reason why it takes the same values in the two extreme regimes corresponding to four-dimensional black holes and to D-branes wrapped on the internal manifold. One can regard the latter picture as a microscopic realization of the quantum states responsible for the black hole entropy and counted by the BPS index. Note that since the BPS index counts bosons and fermions with different signs, it could differ from the actual degeneracy of BPS black holes. Fortunately, it was shown that in most situations this is not the case as all relevant states are bosonic [11,69,70].

However, in general, Ω(γ) does not remain constant under deformations of the (complexified) Kähler moduli of Y, which parametrize the vector multiplet moduli space. This moduli space is divided by the so-called *walls of marginal stability* into chambers with different values of Ω(γ), and the jump of the index between different chambers is called *wall-crossing*. As explained in the Introduction, it happens due to the decay or formation of bound states contributing to the index. Its existence is responsible for many non-trivial phenomena and an extremely rich mathematical structure in theories with eight supercharges.

### 4.2. DT Invariants

From a mathematical viewpoint, BPS indices are a particular instance of *generalized Donaldson–Thomas invariants* [71], which compute the weighted Euler characteristic of the moduli space of coherent sheaves *E* on Y having a Chern vector determined by the charge γ and satisfying a certain stability condition. In particular, the D6-brane charge $p^{0}$ determines the rank of the corresponding sheaf. Although this relation is not crucial for understanding what follows (except Section 8.1), we explain a few useful facts about the generalized DT invariants.

First, we introduce a basis $\left(1,\omega_{a},\omega^{a},\omega_{Y}\right)$ of the even cohomology $H^{\mathrm{even}}\left(Y\right)$ satisfying

(45) $$\omega_{a}\wedge \omega_{b}=\kappa_{abc}\omega^{c},\;\omega_{a}\wedge \omega^{b}=\delta_{a}^{b}\omega_{Y},$$

where $\kappa_{abc}$ are the triple intersection numbers of Y, and combine the charge components into a differential form

(46) $$\gamma \left(E\right)=p^{0}+p^{a}\omega_{a}-q_{a}\omega^{a}+q_{0}\omega_{Y}.$$

Then the D-brane charge and the Chern vector of the corresponding coherent sheaf satisfy the following nice relation [72,73]

(47) $$\gamma \left(E\right)=\mathrm{ch}\left(E\right)\sqrt{\mathrm{Td}(TY)},$$

where Td(TY) is the Todd class of the tangent bundle. Expanding the r.h.s. of (47) in the same cohomology basis, one finds explicit relations between components

(48) $$p^{0}={\mathrm{ch}}_{0},\;p^{a}={\mathrm{ch}}_{1}^{a},\;q_{a}=-{\mathrm{ch}}_{2,a}-\frac{c_{2,a}}{24}\;{\mathrm{ch}}_{0},\;q_{0}={\mathrm{ch}}_{3}+\frac{c_{2,a}}{24}\;{\mathrm{ch}}_{1}^{a},$$

where $c_{2,a}$ are the components of the second Chern class of Y.

Second, to see why the physical BPS indices are only a particular instance of DT invariants, note that, as mentioned above, the definition of the latter involves a stability condition. This condition can be described by a *central charge* $Z_{\gamma }$ [74], which is a complex-valued linear function on the charge lattice and hence can be represented as

(49) $$Z_{\gamma }=q_{\Lambda }X^{\Lambda }-p^{\Lambda }F_{\Lambda },$$

where Λ=(0,a) runs over $b_{2}+1$ values. (The central charge must be supplemented by the so-called heart of a bounded *t*-structure on the derived category of coherent sheaves. We will ignore this in our discussion). The vector $\left(X^{\Lambda },F_{\Lambda }\right)$ parametrizes the space S of stability conditions modulo an action of some symmetry group. The point is that the stability conditions for which the DT invariants coincide with the BPS indices form only a Lagrangian subspace (with respect to the natural symplectic form $dX^{\Lambda }\wedge dF_{\Lambda }$) of the full space S. They are called Π-stability [75] and correspond to the slice $F_{\Lambda }=\partial_{X^{\Lambda }}F\left(X\right)$ where F(X) is the holomorphic prepotential of the special Káhler geometry of the Kähler moduli space of Y. As expected, they are parametrized by the complexified Káhler moduli $z^{a}=b^{a}+it^{a}$.

Next, on the physical slice DT invariants are known to be invariant under monodromy transformations around singularities in the moduli space:

(50) Ω(M·γ;M·z)=Ω(γ;z),

where M represents a monodromy, and we explicitly indicated the (piece-wise constant) dependence on the moduli. It is important to take into account that the monodromy acts not only on charges but also on the moduli, because its action can bring from one chamber to another. A particularly important class of monodromies is around the large volume point $t^{a}=\infty$. They have a simple mathematical interpretation as tensoring the sheaf with a line bundle, leading to the transformation $\mathrm{ch}\left(E\right)\mapsto e^{\epsilon^{a}\omega_{a}}\mathrm{ch}\left(E\right)$ with $\epsilon^{a}\in Z$. In physics, it is known as “spectral flow” transformation and it acts on the moduli by shifting their real part corresponding to the B-field, $b^{a}\mapsto b^{a}+\epsilon^{a}$.

In fact, the large volume region of the moduli space is where most computations are done, and which we are really interested in. In this limit, all quantum corrections to the prepotential become subleading, and it has a simple cubic form

(51) $$F^{\mathrm{cl}}\left(X\right)=-\frac{1}{6X^{0}}\;\kappa_{abc}X^{a}X^{b}X^{c}.$$

Furthermore, the generalized DT invariants of rank 1 (for sufficiently large negative $b^{a}$) coincide with the ordinary DT invariants defined in [76], while for rank −1 (and sufficiently large positive $b^{a}$), they reproduce the so-called Pandharipande–Thomas (PT) invariants [77], up to a torsion factor (see below (185)), if we relax the condition on Y to be simply connected. Note that in these cases some stability walls extend to the large volume region. This is why the limit $t^{a}\to \infty$ of Ω(γ;z) depends on the B-field.

Finally, an important fact is that the generalized DT invariants, and hence the physical BPS indices, satisfy universal wall-crossing formulas [71,78] which are valid for any stability condition. They allow to express Ω(γ;z) on one side of a wall from their values on the other side. We will not present the general formula and restrict ourselves to the simplest case corresponding to the so-called primitive wall-crossing [23], which describes the decay or formation of a bound state of two constituents with charges $\gamma_{1}$ and $\gamma_{2}$. In this case, the jump across the wall is given by

(52) $$\Delta \Omega \left(\gamma ;z\right)={\left(-1\right)}^{\langle \gamma_{1},\gamma_{2}\rangle -1}\left|\langle \gamma_{1},\gamma_{2}\rangle \right|\Omega \left(\gamma_{1},z\right)\Omega \left(\gamma_{2},z\right),$$

where the l.h.s is the difference between DT invariants for $\gamma =\gamma_{1}+\gamma_{2}$ on the two sides of the wall (in the chamber where the bound state exists, minus where it does not), the DT invariants on the r.h.s. are evaluated at the wall, and $\langle \gamma_{1},\gamma_{2}\rangle$ is the anti-symmetric Dirac product of charges, equal to the Euler–Poincaré pairing of the corresponding Chern vectors,

(53) $$\langle \gamma_{1},\gamma_{2}\rangle =p_{2}^{\Lambda }q_{1,\Lambda }-p_{1}^{\Lambda }q_{2,\Lambda }.$$

At least in principle, the knowledge of wall-crossing relations reduces the problem of finding the BPS spectrum on the whole moduli space to finding it in a particular chamber. Of course, in practice, even going from one chamber to another might be non-trivial, not to mention that the chamber structure can itself be very intricate.

### 4.3. Generating Functions of D4-D2-D0 BPS Indices

Let us now specialize to the case of D4-D2-D0 bound states. Since $p^{0}=0$, the BPS indices counting them are the same as rank 0 DT invariants. Our goal in this section will be to assemble these indices into generating functions that have a “nice” behavior under modular transformations.

The first problem that we should solve to this end is due to wall-crossing: given that the BPS indices take different values in different regions of the moduli space, where should they be evaluated to exhibit modular properties? To answer this question, we first note that each charge gives rise to a distinguished point in the moduli space—the *attractor point*. It is provided by the attractor mechanism of N=2 supergravity, which states that, independently of the values of moduli $z^{a}$ at infinity, on the horizon of a single-centered black hole, they take fixed values completely determined by the charge γ [79]. One can also show that in the vicinity of the attractor point, there are no multi-centered black holes except the so-called scaling solutions involving at least three constituents [23,80], so that the BPS index counts only the latter and single-centered ones [81].

However, different charges have different attractor points. Furthermore, on general grounds, one expects that modularity is closely related to the existence of a CFT description [3]. In our case, it can be seen as a holographic description of the ${\mathrm{AdS}}_{3}\times S^{2}$ near horizon geometry in the M-theory picture. In [82], it was shown that the BPS indices counting states in such theory decoupled from the bulk correspond to the DT invariants evaluated at the so-called *large volume attractor point*. The latter is defined as the attractor point for the classical prepotential (51) and a D4-D2-D0 charge rescaled by an infinite factor,

(54) $$z_{\infty }^{a}\left(\gamma \right)=\lim_{\lambda \to +\infty }\left(-\kappa^{ab}q_{b}+i\lambda p^{a}\right),$$

where $\kappa^{ab}$ is the inverse of the quadratic form $\kappa_{ab}=\kappa_{abc}p^{b}$, which is defined by the D4-brane charge and will play an important role from here to the end. Following [24], we denote the resulting BPS indices by

(55) $$\Omega^{\mathrm{MSW}}\left(\gamma \right)=\Omega \left(\gamma ,z_{\infty }\left(\gamma \right)\right)$$

and call them Maldacena–Strominger–Witten (MSW) invariants.

The definition (55) ensures that $\Omega^{\mathrm{MSW}}\left(\gamma \right)$ are invariant under monodromies around the large volume or spectral flow transformations. For vanishing $p^{0}$, they leave the D4-brane charge $p^{a}$ invariant and act on the D2-D0 charges by

(56) $$q_{a}\mapsto q_{a}-\kappa_{ab}\epsilon^{b},\;q_{0}\mapsto q_{0}-\epsilon^{a}q_{a}+\frac{1}{2}\;\kappa_{ab}\epsilon^{a}\epsilon^{b}.$$

The spectral flow parameter ϵ can be thought of as an element of the lattice $\Lambda_{p}=H_{4}\left(Y,Z\right)$ where we put the index *p* to indicate that the lattice is endowed with the quadratic form $\kappa_{ab}$ determined by $p^{a}$. This suggests decomposing the D2-brane charge as

(57) $$q_{a}=\kappa_{ab}\epsilon^{b}+\mu_{a}+\frac{1}{2}\;\kappa_{ab}p^{b},$$

where $\mu \in \Lambda_{p}^{*}/\Lambda_{p}$ with $\Lambda_{p}^{*}=H_{2}\left(Y,Z\right)$ is a residue class similar to those appearing in Section 3, and the last term appears due to a non-trivial quantization condition of $q_{a}$. Then the spectral flow invariance of $\Omega^{\mathrm{MSW}}\left(\gamma \right)$ implies that they depend only on the D4-brane charge $p^{a}$, residue class $\mu_{a}$ and invariant combination of D2 and D0 charges

(58) $${\hat{q}}_{0}=q_{0}-\frac{1}{2}\;\kappa^{ab}q_{a}q_{b}.$$

Thus, we can set $\Omega^{\mathrm{MSW}}\left(\gamma \right)=\Omega_{p,\mu }\left({\hat{q}}_{0}\right)$.

Another important observation made in [83] is that modularity is expected to be manifest not for the integer valued BPS indices Ω(γ), but rather for their rational counterparts defined for a generic charge γ as

(59) $$\bar{\Omega }\left(\gamma \right)=\sum_{d|\gamma }\frac{1}{d^{2}}\;\Omega \left(\gamma /d\right).$$

Another advantage of the rational BPS indices is that they possess simpler properties under wall-crossing [84,85].

The final ingredient necessary for our construction is the Bogomolov–Gieseker bound [86] which states that ${\bar{\Omega }}_{r,\mu }\left({\hat{q}}_{0}\right)$ vanishes unless the invariant charge ${\hat{q}}_{0}$ satisfies

(60) $${\hat{q}}_{0}\leq {\hat{q}}_{0}^{\max}=\frac{1}{24}\;\left(\kappa_{ab}p^{a}p^{b}+c_{2,a}p^{a}\right).$$

Combining it with the previous observations, we arrive at the following definition of generating a series of D4-D2-D0 BPS indices

(61) $$h_{p,\mu }\left(\tau \right)=\sum_{{\hat{q}}_{0}\leq {\hat{q}}_{0}^{\max}}{\bar{\Omega }}_{p,\mu }\left({\hat{q}}_{0}\right)\;q^{-{\hat{q}}_{0}}\;.$$

Since the index μ takes a finite number of values, it can be thought of as a vector index and $h_{p}\left(\tau \right)$ as vector-valued functions labeled by the D4-brane charge. In general, the order of the discriminant group $\Lambda_{p}^{*}/\Lambda_{p}$ where μ takes values equals $|\;\det\;\kappa_{ab}|$, but half of it is redundant due to the symmetry under μ↦−μ following from dualization of the coherent sheaf induced by the D4-brane.

The generating series $h_{p,\mu }\left(\tau \right)$ (61) will be the central object of our study in what follows. In particular, we will be interested in their properties under modular transformations acting on τ and how these properties can be used to find the generating functions explicitly.

## 5. Modular Anomaly

### 5.1. Origin of Modularity

Thus far, the expectation that the generating functions $h_{p,\mu }$ should possess some nice modular properties was based on a connection with CFT. However, it is not very precise, and at this point, it is not known how it can be used to get modular properties of $h_{p,\mu }$ for generic D4-brane charge. Instead, there is another approach based on a target space picture that allows us not only to explain why these functions should be modular, but also to derive their precise behavior under modular transformations.

The idea is to compactify the original setup on an additional circle. After that, we have type IIA string theory on $Y\times S^{1}$, which leads to a three-dimensional effective theory. Since in three dimensions all vector fields can be dualized to scalars, the low-energy effective theory can always be represented as some supersymmetric non-linear sigma model characterized by a moduli space M. The effective action is determined by the metric on M. At the classical level, it can be obtained by the standard Kaluza–Klein reduction of 4d N=2 supergravity coupled to vector multiplets. However, at the quantum level, it receives quantum corrections, both perturbative and non-perturbative. The latter appear as instanton effects due to BPS particles of the four-dimensional theory winding the circle. Therefore, they are characterized by the electro-magnetic charge γ and weighted by the corresponding BPS indices Ω(γ), which roughly count the number of instantons of this charge. Thus, we conclude that the metric on the moduli space M depends on the BPS indices we are interested in.

Furthermore, we actually know this dependence explicitly! First, one should note that from the ten-dimensional viewpoint, the instantons correcting the metric on M arise as D-branes wrapping even-dimensional cycles on Y times the circle $S^{1}$. In particular, we are interested in the instanton corrections generated by D4-branes on a divisor $D_{p}\subset Y$ and $S^{1}$. Second, applying T-duality along the compactification circle, one arrives at type IIB string theory compactified on the same manifold $Y\times S^{1}$. In this T-dual formulation, the moduli space M is nothing but the hypermultiplet moduli space of type IIB on Y since the additional circle compactification does not affect it. All D-instanton corrections to the metric on this moduli space have been computed in a series of works [87,88,89,90] using a twistorial formalism, which allows us to deal with the complicated quaternion-Kähler geometry of M (see [91] for a review). In particular, for D3-instantons, the T-dual of the ones generated by D4-branes in type IIA, it is possible to obtain their corrections to the metric order by order in the expansion in the instanton number [92], and the BPS indices weighting them are the same as we had in the type IIA formulation.

Another important consequence of the duality with type IIB string theory is that it is known to be invariant under the S-duality group, which coincides with SL(2,Z). This invariance manifests in the effective theory obtained by compactification as the existence of an SL(2,Z) isometric action on the hypermultiplet moduli space. Hence, the metric on M must be invariant under SL(2,Z)!

**Figure 1:**
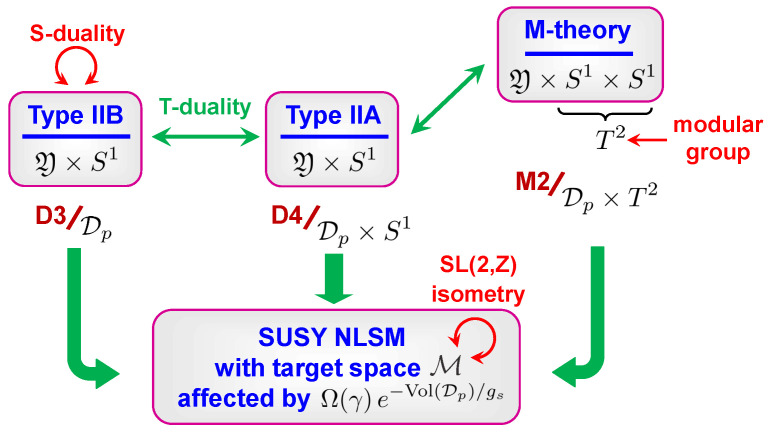
The scheme of dualities illustrating the origin of modularity of D4-D2-D0 BPS indices.

One arrives at the same conclusion if, instead of applying T-duality, one realizes type IIA as M-theory on a circle. Then our setup is equivalent to M-theory compactified on $Y\times T^{2}$, and therefore, it should be invariant under the modular group of the torus. This torus can be seen as the geometric origin of the SL(2,Z) isometry acting on M. We illustrated the different duality frames and the corresponding brane wrappings responsible for the relevant instanton effects in three dimensions in Figure 1.

To summarize, there is a moduli space that, on one hand, has a known dependence on BPS indices and, on the other hand, carries an isometric action of the modular group. Therefore, a natural question is whether this action is consistent with arbitrary values of the BPS indices or imposes some restrictions on them? To answer this question, note that the action of S-duality on quantum effects in the type IIB frame splits into four orbits which, for large Kähler parameters, can be organized in a hierarchical order:

1. D(-1)-instantons mixed with perturbative $g_{s}$ and $\alpha^{\prime }$-corrections;
2. D1-instantons mixed with worldsheet instantons (known also as (p,q)-strings);
3. D3-instantons;
4. D5-instantons mixed with NS5-instantons (known also as (p,q)-five-branes).

One observes that in three of the four orbits, SL(2,Z) mixes different types of quantum corrections. This fact has actually been used to find one type of corrections from a known other type just by applying the method of images [73,93,94,95]. This procedure is independent of the values of Ω(γ) and therefore does not constrain them. At the same time, D3-instantons are invariant under S-duality, which makes them very special. They have been determined *without* imposing S-duality and nevertheless they must respect it. Therefore, one can expect that the compatibility with S-duality imposes certain conditions on the BPS indices associated with these instantons, which are precisely the BPS indices counting D4-D2-D0 bound states in the type IIA formulation. A remarkable fact is that these conditions can be derived explicitly using the knowledge of the instanton corrected metric on M.

### 5.2. Sketch of the Derivation

A derivation of the modular constraints on the D4-D2-D0 BPS indices has been done, first, at one-instanton order in [24], then at two-instanton order in [25] and, finally, at all orders in the instanton expansion in [26]. As we will see, here the instanton order can be associated with the degree of reducibility of the divisor wrapped by the D4-brane, i.e., the number *r* of *irreducible* divisors appearing in the decomposition $D_{p}=\sum_{i=1}^{r}D_{p_{i}}$, or simply $p^{a}=\sum_{i=1}^{r}p_{i}^{a}$. The derivation was done assuming that $D_{p}$ is an *ample* divisor, i.e., the vector $p^{a}$ belongs to the Kähler cone. In the basis of the even homology constructed from the generators of the Kähler cone, the Kähler moduli are positive $t^{a}>0$, the intersections numbers are non-negative $\kappa_{abc}\geq 0$, and the Kähler cone condition ensures that D4-brane charges are also non-negative $p^{a}\geq 0$. In particular, this ensures that $(p^{3})>0$ where we introduced the notation $\left(xyz\right)=\kappa_{abc}x^{a}y^{b}z^{c}$. In this subsection, we present the main steps of the derivation, and if the reader is not interested in this, one can safely skip it.

In fact, for the purpose of obtaining the modular constraints, it is not necessary to work with the full metric on M, which is a highly complicated object. Instead, one can consider a certain function on M, known as *contact potential* $e^{\phi }$ [96], which is related to the four-dimensional string coupling and must be a (non-holomorphic) modular form of weight $\left(-\frac{1}{2},-\frac{1}{2}\right)$ for S-duality to be realized consistently with the quaternion-Kähler property of the metric [87]. Furthermore, in the large volume limit, the part of the contact potential affected by D3-instantons in the type IIB picture can be expressed through an even more fundamental function G, dubbed in [26] as *instanton generating potential*,

(62) (eϕ)D3=τ22ReD−32G+132π2κabctc∂c˜aG∂c˜bG¯,

where $\tau_{2}$ is the inverse of the ten-dimensional string coupling and the imaginary part of the axio-dilaton field $\tau =\tau_{1}+i\tau_{2}$, ${\tilde{c}}_{a}$ is the RR-axion coupled to D3-branes, and $D_{w}$ is the Maass raising operator mapping modular forms of weight $\left(w,\bar{w}\right)$ to modular forms of weight $\left(w+2,\bar{w}\right)$. Since S-duality transforms Kähler moduli as modular forms, $t^{a}\mapsto \left|c\tau +d\right|t^{a}$, and leaves $\partial_{{\tilde{c}}_{a}}$ invariant, the relation (62) shows that one gets the required transformation for the contact potential if and only if G transforms as a modular form of weight $\left(-\frac{3}{2}\;,\;\frac{1}{2}\right)$. Thus, one should just calculate this function in terms of the MSW invariants and find under which conditions it has these transformation properties.

The instanton generating potential has a simple expression in terms of the so-called Darboux coordinates on the twistor space denoted by $X_{\gamma }$. These coordinates are the main object of the twistorial construction of the metric on M because, once they are found, there is a straightforward, albeit non-trivial procedure to get the metric [96]. They are functions of all moduli and an additional coordinate z on the $CP^{1}$-fiber of the twistor bundle over M, which are determined as solutions of the following TBA-like equation

(63) $$X_{\gamma }\left(z\right)=X_{\gamma }^{\mathrm{cl}}\left(z\right)\;\exp\left[\sum_{\gamma^{\prime }\in \Gamma_{+}}\bar{\Omega }\left(\gamma^{\prime }\right)\int_{\ell_{\gamma^{\prime }}}dz^{\prime }\;K_{\gamma \gamma^{\prime }}\left(z,z^{\prime }\right)\;X_{\gamma^{\prime }}\left(z^{\prime }\right)\right],$$

where the dependence on the moduli, not shown explicitly, is hidden in the functions $X_{\gamma }^{\mathrm{cl}}$ (and $K_{\gamma \gamma^{\prime }}$). Here $\Gamma_{+}$ is the lattice of charges with $p^{0}=0$ and $p^{a}$ belonging to the Kähler cone, $\ell_{\gamma }$ is the so-called BPS ray, which in the large volume approximation in the z-plane can be seen as a line passing through the saddle point $z_{\gamma }=-i\left(q_{a}t^{a}+\left(pbt\right)\right)/\left(pt^{2}\right)$, $K_{\gamma \gamma^{\prime }}\left(z,z^{\prime }\right)$ is an integration kernel given by

(64) $$K_{\gamma_{1}\gamma_{2}}\left(z_{1},z_{2}\right)=\frac{1}{2\pi }\left(\left(tp_{1}p_{2}\right)+\frac{i\langle \gamma_{1},\gamma_{2}\rangle }{z_{1}-z_{2}}\right),$$

and $X_{\gamma }^{\mathrm{cl}}$ is the classical limit of the Darboux coordinates, which can be written as

(65) $$X_{\gamma }^{\mathrm{cl}}\left(z\right)=e^{-2\pi i{\hat{q}}_{0}\tau }\;e^{-\pi i\tau {\left(q+b\right)}^{2}+2\pi ic^{a}q_{a}-2\pi \tau_{2}\left(pt^{2}\right){\left(z-z_{\gamma }\right)}^{2}+\ldots },$$

where ${\hat{q}}_{0}$ is the invariant charge (58), $c^{a}$ is the RR-axion coupled to D1-branes, and we dropped some terms irrelevant for our discussion. For what follows, it is important to note that the dependence on ${\hat{q}}_{0}$ factorizes, while the dependence on the charge $q_{a}$ is Gaussian with the quadratic form $q^{2}=\kappa^{ab}q_{a}q_{b}$ determined by $p^{a}$ via $\kappa_{ab}=\kappa_{abc}p^{b}$ and having appeared already in Section 4.3.

**Remark** **2.** *The integral equation (63) is the large volume limit of a more general equation that holds for all charges γ and appears to be identical to the equation put forward in [97], which describes an instanton corrected hyperkähler target space of a three-dimensional sigma model obtained by a circle compactification of a four-dimensional N=2 gauge theory. The identification between D-instantons in string theory on a CY and instantons in N=2 gauge theory on a circle suggested by the coincidence of the equations encoding them has its origin in the QK/HK correspondence [98,99] establishing a relation between the two types of quaternionic manifolds.*

Returning to the function G, it is given by a double integral of the quantum corrected Darboux coordinates

(66) $$G=\frac{1}{4\pi^{2}}\sum_{\gamma \in \Gamma_{+}}\bar{\Omega }\left(\gamma \right)\int_{\ell_{\gamma }}dz\;X_{\gamma }\left(z\right)-\frac{1}{8\pi^{2}}\sum_{\gamma_{1},\gamma_{2}\in \Gamma_{+}}\bar{\Omega }\left(\gamma_{1}\right)\bar{\Omega }\left(\gamma_{2}\right)\int_{\ell_{\gamma_{1}}}dz_{1}\int_{\ell_{\gamma_{2}}}dz_{2}\;K_{\gamma_{1}\gamma_{2}}\left(z_{1},z_{2}\right)\;X_{\gamma_{1}}\left(z_{1}\right)X_{\gamma_{2}}\left(z_{2}\right).$$

To express it in terms of the MSW invariants in a form suitable for extracting their modular properties, one should follow several steps.

1. First, we need to compute the Darboux coordinates $X_{\gamma }\left(z\right)$ to be substituted into (66). Unfortunately, the TBA-like Equation (63) cannot be solved in a closed form. Nevertheless, it can always be solved by iterations: first plug in $X_{\gamma }^{\mathrm{cl}}$ into the r.h.s. to get $X_{\gamma }$ up to the first order, then plug in the result to get the second order, and so on. This procedure produces an asymptotic expansion, which can be seen as an expansion in the number of instantons or, equivalently, in powers of the BPS indices $\bar{\Omega }\left(\gamma \right)$. The resulting perturbative solution can be expressed as a sum over *rooted trees* with vertices labeled by charges. Below we will find many more different trees, so this is our first step into a “forest”.
2. The next step is to substitute the perturbative solution into the instanton generating potential and to re-expand it in powers of $\bar{\Omega }\left(\gamma \right)$. The result can again be written as a sum over trees, but this time these are *unrooted labeled trees*:
  (67) $$G=\frac{1}{4\pi^{2}}\sum_{n=1}^{\infty }\left[\prod_{i=1}^{n}\frac{1}{n!}\sum_{\gamma_{i}\in \Gamma_{+}}\bar{\Omega }\left(\gamma_{i}\right)\int_{\ell_{\gamma_{i}}}dz_{i}\;X_{\gamma_{i}}^{\mathrm{cl}}\left(z_{i}\right)\right]\sum_{T\in \;T_{n}^{\ell }}\prod_{e\in E_{T}}K_{\gamma_{s(e)},\gamma_{t(e)}}\left(z_{s(e)},z_{t(e)}\right),$$
  where *n* is the number of vertices, the last product goes over all edges of a tree T, and s(e), t(e) denote the source and target vertex of an edge *e*.
3. The expansion (67) is not yet exactly what we want because it is expressed through the rational DT invariants $\bar{\Omega }\left(\gamma \right)$, while we are looking for an expression in terms of the MSW invariants $\Omega^{\mathrm{MSW}}\left(\gamma \right)$. The difference between them is due to the fact explained in Section 4.2 that the DT invariants are not actually constant, but depend on the moduli. Fortunately, it is possible to express $\bar{\Omega }\left(\gamma ;z\right)$ through $\Omega^{\mathrm{MSW}}\left(\gamma \right)$ using the *split attractor flow conjecture*, which allows to count contributions of all bound states to an index in terms of indices evaluated at their attractor points [23,100]. The result is represented as a sum over all possible consecutive splits of bound states into their constituents and can be conveniently written as
  (68) $$\bar{\Omega }\left(\gamma ,z\right)=\sum_{\sum_{i=1}^{n}\gamma_{i}=\gamma }g_{\mathrm{tr},n}\left(\left\{\gamma_{i}\right\},z\right)\;\prod_{i=1}^{n}{\bar{\Omega }}^{\mathrm{MSW}}\left(\gamma_{i}\right),$$
  where the sum runs over ordered decompositions of charge γ into elements of $\Gamma_{+}$ and the weight $g_{\mathrm{tr},n}\left(\left\{\gamma_{i}\right\},z\right)$ is the so-called *tree index*. It is given by a sum over yet another type of trees known as *attractor flow trees*, which are binary rooted trees with *n* leaves labeled by $\gamma_{i}$ and other vertices labeled by charges equal to the sum of the charges of their children. The contribution of each tree is a simple combination of factors given by the Dirac product of charges (53) and certain sign functions responsible for the piece-wise constant moduli dependence of $\bar{\Omega }\left(\gamma ,z\right)$. In [85], an alternative representation for the tree index has been found that provided important hints for the construction explained below.
4. After one substitutes (68) into (67), one can make two observations. First, since for charges with vanishing $p^{0}$ the tree index is independent of their $q_{0}$ components, the only dependence on these components is in $X_{\gamma_{i}}^{\mathrm{cl}}$ through the factor $e^{-2\pi i{\hat{q}}_{i,0}\tau }$ (see (65)) and in the MSW invariants. Therefore, the sum over $q_{0,i}$ gives rise precisely to the generating functions $h_{p_{i},\mu_{i}}\left(\tau \right)$ (61) where $\mu_{i}$ are the residue classes appearing in the decomposition (57) of charges $q_{i,a}$. Second, due to the spectral flow invariance, the MSW invariants and hence their generating functions are independent of the parameters $\epsilon_{i}$ also appearing in the decomposition (57). As a result, the sum over these parameters produces some theta series with non-trivial kernels $\Phi_{n}^{\mathrm{tot}}$ constructed from the integrals appearing in (67) and the tree indices arising from DT invariants. Combining everything together, one arrives at the following representation
  (69) $$G=\sum_{n=1}^{\infty }\left[\prod_{i=1}^{n}\sum_{p_{i},\mu_{i}}h_{p_{i},\mu_{i}}\right]\vartheta_{p,\mu }\left(\Lambda_{p},\Phi_{n}^{\mathrm{tot}}\right),$$
  where we used boldface letters to denote tuples of *n* variables like $p=(p_{1},\ldots ,p_{n})$. The theta series $\vartheta_{p,\mu }$ is of type (42) defined by the lattice $\Lambda_{p}={⊕}_{i=1}^{n}\Lambda_{p_{i}}$ with the quadratic form
  (70) $$k^{2}=\sum_{i=1}^{n}\kappa_{i,ab}k_{i}^{a}k_{i}^{b},\;\kappa_{i,ab}=\kappa_{abc}p_{i}^{c}.$$
  As follows from the Hodge index theorem, for $p^{a}$ corresponding to an ample divisor, the associated quadratic form $\kappa_{ab}$ has signature $\left(1,b_{2}-1\right)$. Since $D_{p_{i}}$ are also ample, $\vartheta_{p,\mu }$ is an indefinite theta series of signature $\left(n,\left(b_{2}-1\right)n\right)$. Its convergence is ensured by the integrals in (67) entering the kernel, which can be shown to decay exponentially along the “dangerous” directions of the lattice.
5. Since G must be a modular form, the representation (69) implies that the modular properties of $h_{p,\mu }$ are determined by the modular properties of the theta series: if $\vartheta_{p,\mu }\left(\Lambda_{p},\Phi_{n}^{\mathrm{tot}}\right)$ are all modular, the generating functions are also modular; if not—$h_{p,\mu }$ must have a modular anomaly to cancel the anomaly of the theta series. The easiest way to check the modularity of $\vartheta_{p,\mu }\left(\Lambda_{p},\Phi_{n}^{\mathrm{tot}}\right)$ is to verify whether its kernel $\Phi_{n}^{\mathrm{tot}}$ satisfies the differential Equation (43). It turns out that all the non-trivial integrals pass through this equation, whereas the sign functions coming from the tree indices spoil it. Thus, it is the existence of bound states and the corresponding wall-crossing that are responsible for the appearance of a modular anomaly, exactly as in the story about immortal dyons in N=4 compactifications [16].
6. Once the origin of the anomaly in each term with fixed *n* has been identified, one can try to “improve” the expansion (69) by reshuffling it. Namely, we can look for the modular completion of the theta series and ask whether this completion can be achieved by “redefining” the generating functions $h_{p,\mu }$. This is an extremely non-trivial problem because the theta series depend on *all* scalar fields of the effective theory (playing the role of coordinates on the moduli space M), while $h_{p,\mu }$ are functions of only the axio-dilaton τ. Nevertheless, the above idea can be realized! It leads to a new representation
  (71) $$G=\sum_{n=1}^{\infty }\left[\prod_{i=1}^{n}\sum_{p_{i},\mu_{i}}{\hat{h}}_{p_{i},\mu_{i}}\right]\vartheta_{p,\mu }\left(\Lambda_{p},{\hat{\Phi }}_{n}^{\mathrm{tot}}\right),$$
  where the new kernels ${\hat{\Phi }}_{n}^{\mathrm{tot}}$ are such that the corresponding theta series are modular. This implies that the new functions ${\hat{h}}_{p,\mu }$, which are now non-holomorphic, should also be modular of weight $\left(-\frac{1}{2}b_{2}-1,0\right)$. Thus, they can be considered as modular completions of the original generating series and their form encodes the modular anomaly of $h_{p,\mu }$.

### 5.3. Equation for the Modular Completion

The explicit form of the modular completion ${\hat{h}}_{p,\mu }$, derived in [26] and later corrected and simplified in [27], is the main result containing all information about the modular behavior of the generating series $h_{p,\mu }$. Its schematic form has been already presented in the Introduction in (3). Here we will explain it in detail.

If *r* is the degree of reducibility of divisor $D_{p}$, then the completion is given by

(72) $${\hat{h}}_{p,\mu }\left(\tau ,\bar{\tau }\right)=h_{p,\mu }\left(\tau \right)+\sum_{n=2}^{r}\sum_{\sum_{i=1}^{n}p_{i}=p}\sum_{\mu }R_{\mu ,\mu }^{\left(p\right)}\left(\tau ,\bar{\tau }\right)\prod_{i=1}^{n}h_{p_{i},\mu_{i}}\left(\tau \right),$$

where the coefficients $R_{\mu ,\mu }^{\left(p\right)}$ can be written as a sum over D2-brane charges $q_{i,a}$ with fixed residue classes $\mu_{i,a}$ and a fixed total sum:

(73) Rμ,μ(p)(τ,τ¯)=∑∑i=1nqi=μ+12pqi∈Λpi+μi+12piSym(−1)∑i<jγijRn(γ^;τ2)eπiτQn(γ^).

Here $\hat{\gamma }$ is the *n*-tuple of reduced charge vectors ${\hat{\gamma }}_{i}=\left(p_{i}^{a},q_{i,a}\right)$, Sym denotes symmetrization (with weight 1/n!) with respect to charges ${\hat{\gamma }}_{i}$, $\gamma_{ij}=\langle {\hat{\gamma }}_{i},{\hat{\gamma }}_{j}\rangle$, and $Q_{n}\left(\hat{\gamma }\right)$ is a the quadratic form on $\Lambda_{p}/\Lambda_{p}$

(74) $$Q_{n}\left(\hat{\gamma }\right)=\kappa^{ab}q_{a}q_{b}-\sum_{i=1}^{n}\kappa_{i}^{ab}q_{i,a}q_{i,b}\;.$$

It is clear that $R_{\mu ,\mu }^{\left(p\right)}$ can be seen as indefinite theta series on $\Lambda_{p}/\Lambda_{p}$. All non-trivialities are hidden in the functions $R_{n}\left(\hat{\gamma };\tau_{2}\right)$, which play the role of kernels of these theta series. Their definition involves two types of trees and proceeds in two steps.

At the first step, we consider the set $T_{n}^{\ell }$ of *unrooted labeled trees*, as in (67), with *n* vertices decorated by charges from the set $\hat{\gamma }=\left({\hat{\gamma }}_{1},\ldots ,{\hat{\gamma }}_{n}\right)$. Given a tree $T\in T_{n}^{\ell }$, we denote the set of its edges by $E_{T}$, the set of vertices by $V_{T}$, the source and target vertex of an edge *e* by s(e) and t(e), respectively, and the two disconnected trees obtained from T by removing the edge *e* by $T_{e}^{s}$ and $T_{e}^{t}$. (The orientation of edges on a given tree can be chosen arbitrarily, and the final result does not depend on this choice). Furthermore, to each edge we assign the vector

(75) $$v_{e}=\sum_{i\in V_{T_{e}^{s}}}\sum_{j\in V_{T_{e}^{t}}}v_{ij},$$

where $v_{ij}$ are $nb_{2}$-dimensional vectors with the following components

(76) $${\left(v_{ij}\right)}_{k}^{a}=\delta_{ki}p_{j}^{a}-\delta_{kj}p_{i}^{a}.$$

Using these notations, we define functions of $\tau_{2}$ parametrized by *n* reduced charges ${\hat{\gamma }}_{i}=\left(p_{i}^{a},q_{i,a}\right)$ and constructed from the generalized error functions (A2). To this end, we introduce

(77) $$\Phi_{n}^{\;E}\left(x\right)=\frac{1}{n!}\sum_{T\in \;T_{n}^{\ell }}\left[\prod_{e\in E_{T}}D\left(v_{s(e)t(e)},y\right)\right]\Phi_{n-1}^{E}\left(\left\{v_{e}\right\};x\right){|}_{y=x},$$

where

(78) $$D\left(v,y\right)=v\cdot \left(y+\frac{1}{2\pi }\;\partial_{x}\right)$$

and the dot in (78) denotes the bilinear form

(79) $$x\cdot y=\sum_{i=1}^{n}\kappa_{i,ab}x_{i}^{a}y_{i}^{b},$$

which is also used to define the generalized error functions $\Phi_{n}^{E}$ (A2). It is useful to note that with respect to this bilinear form one has $v_{ij}\cdot q=\gamma_{ij}$ where $q=\left(\kappa_{1}^{ab}q_{1,b},\ldots ,\kappa_{n}^{ab}q_{n,b}\right)$. In terms of the vector q, our functions are given by

(80) $$E_{n}\left(\hat{\gamma };\tau_{2}\right)=\frac{\Phi_{n}^{\;E}\left(\sqrt{2\tau_{2}}\;q\right)}{{\left(\sqrt{2\tau_{2}}\right)}^{n-1}}\;.$$

An important fact is that these functions have a canonical decomposition

(81) $$E_{n}\left(\hat{\gamma };\tau_{2}\right)=E_{n}^{\left(0\right)}\left(\hat{\gamma }\right)+E_{n}^{\left(+\right)}\left(\hat{\gamma };\tau_{2}\right),$$

where the first term $E_{n}^{\left(0\right)}$ does not depend on $\tau_{2}$, whereas the second term $E_{n}^{\left(+\right)}$ is exponentially suppressed as $\tau_{2}\to \infty$ keeping the charges ${\hat{\gamma }}_{i}$ fixed. In [27], it was shown that

(82) $$E_{n}^{\left(0\right)}\left(\hat{\gamma }\right)=\frac{1}{n!}\sum_{T\in \;T_{n}^{\ell }}S_{T}\left(\hat{\gamma }\right)\prod_{e\in E_{T}}\gamma_{s(e)t(e)},$$

where $S_{T}\left(\hat{\gamma }\right)$ is a product of sign functions of the following combinations of Dirac products

(83) $$\Gamma_{e}=\sum_{i\in V_{T_{e}^{s}}}\sum_{j\in V_{T_{e}^{t}}}\gamma_{ij}=v_{e}\cdot q.$$

However, it cannot be written as a simple product of $\mathrm{sgn}(\Gamma_{e})$ because, when an even number of $\Gamma_{e}$’s vanish, it is not actually zero, but equals a rational number. This means that the correct formula can be written as

(84) $$S_{T}\left(\hat{\gamma }\right)=\sum_{J\subseteq E_{T}}e_{T_{J}}\;\prod_{e\in J}\delta_{\Gamma_{e}}\prod_{e\in E_{T}\setminus J}\mathrm{sgn}\left(\Gamma_{e}\right),$$

where $T_{J}$ denotes the tree obtained from T by contracting the edges $e\in E_{T}\setminus J$, and $e_{T}$ are the above-mentioned rational numbers. They depend only on the topology of T, vanish for trees with an even number of vertices, and can be computed with the help of a recursive formula. This formula involves yet another rational numbers $a_{T}$ for which there is their own recursive formula:

(85) $$a_{T}=\frac{1}{n_{T}}\sum_{v\in V_{T}}{\left(-1\right)}^{n_{v}^{+}}\prod_{s=1}^{n_{v}}a_{T_{s}\left(v\right)},$$

where $n_{T}$ is the number of vertices, $n_{v}$ is the valency of the vertex v, $n_{v}^{+}$ is the number of incoming edges at the vertex, and $T_{s}\left(v\right)$ are the trees obtained from T by removing the vertex. Having computed $a_{T}$, one can obtain $e_{T}$ from

(86) $$e_{T}=-\sum_{m=1}^{n_{T}-1}\sum_{\overset{m}{\underset{k=1}{\cup }}T_{k}≃T}\;e_{T/\{T_{k}\}}\prod_{k=1}^{m}a_{T_{k}}.$$

where the second sum runs over all decompositions of T into a set of non-intersecting subtrees and $T/\{T_{k}\}$ denotes the tree obtained from T by collapsing each subtree $T_{k}$ to a single vertex. Both recursions are initiated by the values $e_{•}=a_{•}=1$ for a single vertex tree. The values of both $e_{T}$ and $a_{T}$ for trees with $n_{T}\leq 7$ can be found in ([27], Ap.B).

**Figure 2:**
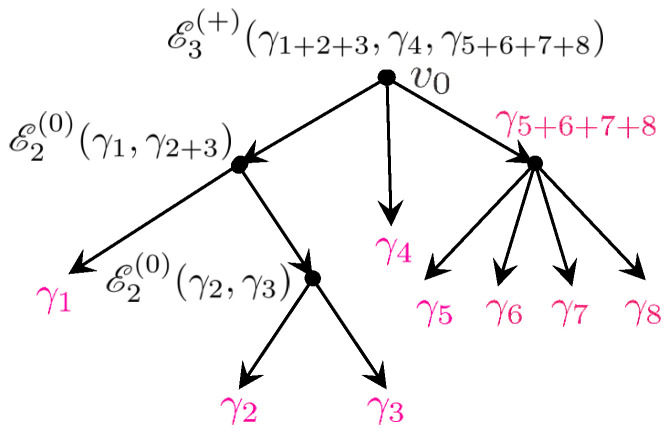
An example of Schröder tree contributing to $R_{8}$. Near each vertex we showed the corresponding factor using the shorthand notation $\gamma_{i+j}=\gamma_{i}+\gamma_{j}$.

At the second step, we introduce a new type of trees known as *Schröder trees*. They are defined as rooted planar trees such that all vertices $v\in V_{T}$ (the set of vertices of *T* excluding the leaves) have $k_{v}\geq 2$ children. The set of such trees with *n* leaves will be denoted by $T_{n}^{S}$. Furthermore, we take $n_{T}$ to be the number of elements in $V_{T}$ and $v_{0}$ to denote the root vertex. The vertices of *T* are labeled by charges in a way similar to attractor flow trees: the leaves carry charges ${\hat{\gamma }}_{i}$, whereas the charges assigned to other vertices are given recursively by the sum of charges of their children, ${\hat{\gamma }}_{v}\in \sum_{v^{\prime }\in \mathrm{Ch}\left(v\right)}{\hat{\gamma }}_{v^{\prime }}$. Then, given a Schröder tree *T*, we set $E_{v}\equiv E_{k_{v}}\left(\left\{{\hat{\gamma }}_{v^{\prime }}\right\}\right)$ (and similarly for $E_{v}^{\left(0\right)},E_{v}^{\left(+\right)}$) where $v^{\prime }\in \mathrm{Ch}\left(v\right)$ runs over the $k_{v}$ children of the vertex *v* (see Figure 2). Using these notations, we can finally write a formula for the coefficients $R_{n}$:

(87) $$R_{n}\left(\hat{\gamma };\tau_{2}\right)=\frac{1}{2^{n-1}}\sum_{T\in T_{n}^{S}}{\left(-1\right)}^{n_{T}-1}E_{v_{0}}^{\left(+\right)}\prod_{v\in V_{T}\setminus \left\{v_{0}\right\}}E_{v}^{\left(0\right)}.$$

**Figure 3:**
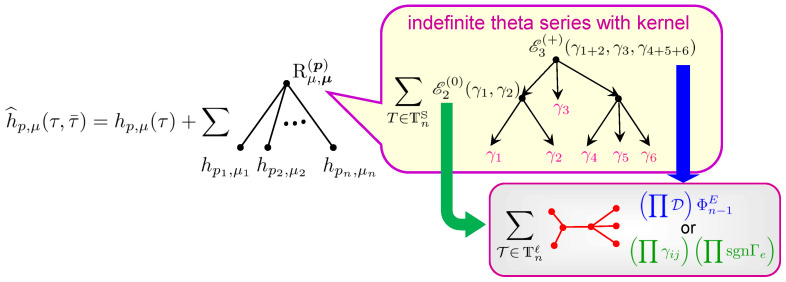
A schematic representation of the modular completion (72). The first sum over decompositions of the D4-brane charge $p^{a}$ can be seen as a sum over rooted trees of depth one with generating series $h_{p_{i},\mu_{i}}$ assigned to the leaves and the indefinite theta series $R_{\mu ,\mu }^{\left(p\right)}$ assigned to the root. The kernel of the theta series is a sum over Schröder trees and the functions assigned to their vertices are sums over unrooted labeled trees.

This completes the definition of the objects appearing in the expression (72) of the modular completion. Although the above construction appears to be complicated (a simpler version will be presented in Section 6.3 after incorporating an additional refinement parameter), its mathematical structure is transparent: Equation (72) expresses the completion as a sum over all possible bound states with a given D4-brane charge, Equation (73) represents the coefficients of this expansion as indefinite theta series on $\Lambda_{p}/\Lambda_{p}$, while Equation (87) builds up kernels of the theta series as a sum over Schröder trees, which can be seen to encode different decay channels of bound states, weighted by (derivatives of) the generalized error functions and their limit at large $\tau_{2}$ where they reduce to a combination of sign functions (see Figure 3). As explained in Section 3, both building blocks are very natural in the context of indefinite theta series.

Furthermore, since indefinite theta series are the classic examples of higher depth mock modular forms, it is natural to expect that the generating functions $h_{p,\mu }$ belong to the same class. In fact, one can explicitly compute the non-holomorphic derivative of the completion encoding the shadow. The result reads as

(88) $$\partial_{\bar{\tau }}{\hat{h}}_{p,\mu }\left(\tau ,\bar{\tau }\right)=\sum_{n=2}^{r}\sum_{\sum_{i=1}^{n}{\hat{\gamma }}_{i}=\hat{\gamma }}{\left(-1\right)}^{\sum_{i<j}\gamma_{ij}}J_{n}\left(\hat{\gamma },\tau_{2}\right)\;e^{\pi i\tau Q_{n}\left(\hat{\gamma }\right)}\prod_{i=1}^{n}{\hat{h}}_{p_{i},\mu_{i}}\left(\tau ,\bar{\tau }\right),$$

where $\hat{\gamma }=\left(p^{a},\mu_{a}+\frac{1}{2}\kappa_{ab}p^{b}\right)$ and

(89) $$J_{n}\left(\hat{\gamma },\tau_{2}\right)=\frac{i}{2^{n}}\sum_{T\in T_{n}^{S}}{\left(-1\right)}^{n_{T}-1}\partial_{\tau_{2}}E_{v_{0}}\prod_{v\in V_{T}\setminus \left\{v_{0}\right\}}E_{v}.$$

It is expressed through modular forms, the completions ${\hat{h}}_{p_{i},\mu_{i}}$ and an indefinite theta series with kernel $J_{n}$ satisfying the Vignéras Equation (43), consistently with the fact that $\partial_{\bar{\tau }}{\hat{h}}_{p,\mu }$ is a modular form of weight $\left(-\frac{1}{2}b_{2}-1,2\right)$. Since the shadow is expressed through the same types of objects as the completion, but the derivative decreases the rank of the generalized error functions by 1, the result (89) confirms that $h_{p,\mu }$ are mock modular forms of depth r−1. In particular, for r=1 the second term in (72) is absent and the generating series are ordinary modular forms, which is consistent with the previous results [7,22,23]. The first non-trivial case appears at r=2 and exhibits the mixed mock modularity.

Independently of *r*, the multiplier system of $h_{p,\mu }$ is obtained as the inverse of the multiplier system of $\vartheta_{p,\mu }\left(\Lambda_{p},{\hat{\Phi }}_{1}^{\mathrm{tot}}\right)$ (see (71)) and is given by

(90) $$\begin{array}{cc} M_{\mu \nu }\left(T\right)= & \;e^{\pi i{\left(\mu +\frac{p}{2}\right)}^{2}+\frac{\pi i}{12}\;c_{2,a}p^{a}}\;\delta_{\mu \nu },\\ M_{\mu \nu }\left(S\right)= & \;\frac{{\left(-1\right)}^{\chi (O_{D_{p}})}}{\sqrt{|\;\det\;\kappa_{ab}|}}\;e^{\left(b_{2}-2\right)\frac{\pi i}{4}}\;e^{-2\pi i\mu \cdot \nu }\;, \end{array}$$

where $\mu \cdot \nu =\kappa^{ab}\mu_{a}\nu_{b}$ and $\chi \left(O_{D_{p}}\right)=\frac{1}{2}\left(b_{2}^{+}\left(D_{p}\right)+1\right)$ is the arithmetic genus of the divisor expressed in terms of the D4-brane charge as

(91) $$\chi \left(O_{D_{p}}\right)=\frac{1}{6}\;\left(p^{3}\right)+\frac{1}{12}\;c_{2,a}p^{a}.$$

#### 5.3.1. Collinear Charges

Before we proceed further, it is worth considering a special case that has many interesting applications. Let $p_{0}^{a}$ corresponds to an irreducible divisor. Since in (72) one sums over decompositions of $D_{p}$ only in ample divisors, for $p^{a}=rp_{0}^{a}$ only collinear charges $p_{i}^{a}=r_{i}p_{0}^{a}$ can appear, and the sum is equivalent to the sum over decompositions $r=\sum_{i}r_{i}$. It turns out that when all charges are collinear, the holomorphic anomaly (88) of the completion is enormously simplified. In [28] it was proven that in this case $J_{n}=0$ for all n>2. Thus, the only term that survives is the one with n=2! It can be computed explicitly, and the resulting anomaly equation takes the following form

(92) ∂τ¯h^rp0,μ=(p03)8πi(2τ2)3/2∑r1+r2=rq1+q2=μ+12r2p0(−1)γ12rr1r2e−2πτ2γ122rr1r2(p03)eπiτQ2(γ^i,γ^2)h^r1p0,μ1h^r2p0,μ2,

where $\gamma_{12}=p_{0}^{a}\left(r_{2}q_{1,a}-r_{1}q_{2,a}\right)$. Note that this result does *not* imply that a similar cancellation happens in the expression for the completion (72), and $h_{rp_{0},\mu }$ are still mock modular forms of depth r−1.

The holomorphic anomaly (92) can be further simplified by introducing a partition function, which can be identified with the modified elliptic genus [20] of the SCFT mentioned in the Introduction. It is defined by

(93) $${\hat{Z}}_{r}=\sqrt{\frac{\left(p_{0}^{3}\right)}{r}}\;\sum_{\mu }{\hat{h}}_{rp_{0},\mu }\;\bar{\vartheta_{rp_{0},\mu }^{\left(S\right)}\left(\tau ,v\right)},$$

where $\vartheta_{p,\mu }^{\left(S\right)}$ is a Jacobi extension of the Siegel theta series (36) on the lattice $\Lambda_{p}$

(94) $$\vartheta_{p,\mu }^{\left(S\right)}\left(\tau ,v\right)=\sum_{q\in \Lambda_{p}+\mu +\frac{1}{2}p}\sigma_{\gamma }\;e^{-2\pi \tau_{2}q_{p}^{2}+\pi i\bar{\tau }q^{2}+2\pi iq_{a}v^{a}}$$

and we denoted $q^{2}=\kappa^{ab}q_{a}q_{b}$ and $q_{p}=q_{a}p^{a}/\sqrt{\left(p^{3}\right)}$ playing the role of the projection on the positive definite sublattice (denoted by + in (36)), which in this case is one-dimensional. The factor $\sigma_{\gamma }$ is just a sign factor satisfying $\sigma_{\gamma_{1}}\sigma_{\gamma_{2}}={\left(-1\right)}^{\gamma_{12}}\sigma_{\gamma_{1}+\gamma_{2}}$, which is known as quadratic refinement. Since the Siegel theta series is a modular form of weight $\left(\frac{1}{2},\frac{1}{2}\;\left(b_{2}-1\right)\right)$, the partition function ${\hat{Z}}_{r}$ is also modular with weight $\left(-\frac{3}{2}\;,\;\frac{1}{2}\right)$. Furthermore, the theta series almost cancels the multiplier system of ${\hat{h}}_{rp_{0},\mu }$, so that one remains with ${\left(M^{\left(\eta \right)}\right)}^{rp_{0}^{a}c_{2,a}}$ where $M^{\left(\eta \right)}$ is the multiplier system (11) of the Dedekind eta function. (It can also be canceled by multiplying the partition function by $e^{2\pi irp_{0}^{a}{\tilde{c}}_{a}}$ where ${\tilde{c}}_{a}$ is the RR-field appearing in (62)). Given (92), it is straightforward to check that [28]

(95) $$\bar{D}{\hat{Z}}_{r}=\frac{\sqrt{2\tau_{2}}}{32\pi i}\sum_{r_{1}+r_{2}=r}r_{1}r_{2}\;{\hat{Z}}_{r_{1}}{\hat{Z}}_{r_{2}},$$

where

(96) $$\bar{D}=\tau_{2}^{2}\left(\partial_{\bar{\tau }}-\frac{i}{4\pi }\;\partial_{v_{+}}^{2}\right)$$

is designed to commute with the Siegel theta series and, acting on the completion of a mock modular form, decreases its holomorphic weight by two. Such a holomorphic anomaly equation has been found, for example, for the completion of the elliptic genus of the $\frac{1}{2}K3$ surface [101]. The result (95) shows that this is a much more general and universal phenomenon taking place as soon as the relevant magnetic charges are all collinear.

## 6. Extensions

The expression for the modular completion presented in the previous section has been derived in a concrete setup: D4-brane wrapping an ample divisor of a compact CY threefold in compactified type IIA string theory. However, it turns out that some conditions, like the ampleness of the divisor, can be relaxed, and the whole construction can be extended either to more general settings or to include additional parameters. In this section, we describe three such extensions.

### 6.1. Degenerations

First, we drop the assumption that the divisor wrapped by D4-brane is ample and replace it by a weaker condition that it should be *effective*. (In the basis of the Kähler cone used above, the components of the magnetic charge corresponding to an effective divisor can be negative. However, since effective divisors form a cone, there is a basis where $p^{a}\geq 0$. The price to pay is that the intersection numbers can be negative in this basis). In particular, we are interested in the situation when the charge $p^{a}$ satisfies

(97) $$\left(p^{3}\right)=\kappa_{abc}p^{a}p^{b}p^{c}=0,$$

which was not allowed in the original construction. Nevertheless, as we will see now, one can still make sense of it. However, one should distinguish between two very different cases.

#### 6.1.1. Degenerate Quadratic Form

The first case we need to consider is when the quadratic form $\kappa_{ab}$ is degenerate. Namely, it has at least one zero eigenvalue. In [33] it was argued that in such situation the above construction of the modular completion still holds provided the lattice used to sum over D2-brane charges is restricted to the non-degenerate part of the full original lattice. More precisely, let $\lambda_{s}^{a}$ be a set of null eigenvectors, i.e., $\kappa_{ab}\lambda_{s}^{a}=0$. Then we take

(98) $$\Lambda_{p}^{*}=\left\{q_{a}\in Z+\frac{1}{2}\;\kappa_{ab}p^{b}\;:\;\lambda_{s}^{a}q_{a}=0\right\}.$$

Furthermore, one can introduce the inverse quadratic form $\kappa^{ab}$ known as Moore–Penrose or pseudoinverse of $\kappa_{ab}$. It is defined by the conditions (i) $\mathrm{rank}\left(\kappa^{ab}\right)=\mathrm{rank}\left(\kappa_{ab}\right)$, (ii) $\kappa^{ac}\kappa_{cb}=\delta_{b}^{a}-\sum_{s,t}e^{st}\lambda_{s}^{a}\lambda_{t,b}$ where $e^{st}$ is the inverse of $e_{st}=\lambda_{s}^{a}\lambda_{t,a}$. Using these definitions in the equations of Section 5.3 leads to a well-defined modular completion. The only change to be made is to replace $b_{2}$ by $\mathrm{rank}\left(\Lambda_{p}\right):=\mathrm{rank}\left(\kappa_{ab}\right)$. In particular, the weight of the generating functions is now given by

(99) $$w\left(p\right)=-\frac{1}{2}\;\mathrm{rank}\left(\Lambda_{p}\right)-1.$$

If $p^{a}$ is one of the null eigenvectors, one gets an even stronger condition than (97),

(100) $$\kappa_{abc}p^{b}p^{c}=0.$$

Let us assume for simplicity that $p^{a}$ is a multiple of $p_{0}^{a}$ representing an irreducible effective divisor. It turns out that in this case the modular anomaly completely disappears! Indeed, it is trivial to see that all reduced charges ${\hat{\gamma }}_{i}=\left(p_{i}^{a},q_{i,a}\right)$ corresponding to the bound state constituents have $p_{i}^{a}=r_{i}p_{0}^{a}$ and $q_{i,a}$ orthogonal to $p_{0}^{a}$ and therefore satisfy $\langle \gamma_{i},\gamma_{j}\rangle =0$. As a result, the modular anomaly simply does not arise in this case because all its sources such as multi-instanton contributions to the instanton generating potential or bound state contributions to DT invariants are weighted by the Dirac products $\langle \gamma_{i},\gamma_{j}\rangle$ and hence vanish. Thus, the generating functions $h_{p,\mu }$ with $p^{a}=rp_{0}^{a}$ satisfying (100) must be modular forms. This is the case relevant, for example, for vertical divisors of K3-fibered CYs [102,103,104,105]. It is likely that this conclusion continues to hold even for more general effective divisors satisfying (100). To prove this, one should show that $\langle \gamma_{i},\gamma_{j}\rangle =0$ for all possible decay products.

If, however, $p^{a}$ is not a null eigenvector of the quadratic form that it defines, the reasoning leading to the vanishing of all relevant Dirac products $\langle \gamma_{i},\gamma_{j}\rangle$ does not hold anymore and the generating functions $h_{p,\mu }$ can still have very non-trivial mock modular properties encoded by their completions (72). This case will be relevant below in Section 6.2.

#### 6.1.2. Non-Degenerate Quadratic Form

In the second case, $(p^{3})=0$, but the quadratic form is non-degenerate. This case has not been analyzed before in the literature; therefore, we consider it here in detail. Let us again assume that $p^{a}=rp_{0}^{a}$ where $p_{0}^{a}$ is an irreducible effective divisor satisfying $(p_{0}^{3})=0$, so that all charges are collinear as in Section 5.3.1.

Then the holomorphic anomaly of the completion is greatly simplified and should be given by (92). However, this formula is not directly applicable to our case. On one hand, due to $(p_{0}^{3})=0$, the first exponential factor vanishes so that one might think that the anomaly disappears and the generating functions become usual modular forms as in the previous case. On the other hand, for charges satisfying $\gamma_{12}=0$, this reasoning fails because the exponential does not vanish anymore. It is tempting to say that, even for these charges, the vanishing is still ensured by the overall factor $\sqrt{\left(p_{0}^{3}\right)}$. But the problem is that the remaining theta series is actually divergent: since $p_{0}^{a}$ is a null vector, the component of D2-brane charges along this vector does not contribute to the quadratic form $Q_{2}\left({\hat{\gamma }}_{i},{\hat{\gamma }}_{2}\right)$. As a result, we arrive at ambiguity 0×∞.

In Appendix D we show how this ambiguity can be resolved by taking the magnetic charge slightly off the null direction and then removing the regularization. The result of this analysis is the following holomorphic anomaly equation

(101) $$\partial_{\bar{\tau }}\;{\hat{h}}_{rp_{0},\mu }\left(\tau ,\bar{\tau }\right)=\frac{\tau_{2}^{-2}}{16\pi i}\sum_{r_{1}+r_{2}=r}h_{p_{0},\mu }^{\left(r_{1},r_{2}\right)}\left(\tau ,\bar{\tau }\right),$$

where

(102) $$h_{p_{0},\mu }^{\left(r_{1},r_{2}\right)}\left(\tau ,\bar{\tau }\right)=r_{0}\sum_{\mu_{1},\mu_{2}}\delta_{\Delta \mu \in r_{0}\Lambda_{0}}\;{\hat{h}}_{r_{1}p_{0},\mu_{1}}\;{\hat{h}}_{r_{2}p_{0},\mu_{2}}\sum_{A=0}^{n_{g}-1}\delta_{p_{0}\cdot \left(\mu_{12}^{\|}+r_{12}g_{A}^{\left|\right|}\right)}^{\left(\xi r_{12}\right)}\vartheta_{\mu_{12}^{⊥}+r_{12}g_{A}^{⊥}}^{⊥},$$

is a modular form of weight $\left(-\frac{1}{2}b_{2}-3,0\right)$, $\delta_{x}^{\left(n\right)}$ is the mod-*n* Kronecker delta defined by

(103) $$\delta_{x}^{\left(n\right)}=\left\{\begin{array}{cc} 1\; & \mathrm{if}x=0\;\;\;\;\mathrm{mod}\;n,\\ 0\; & \mathrm{otherwise}, \end{array}\right.$$

while other notations can be found in Appendix D. A remarkable feature of the anomaly Equation (101) is that the non-holomorphic dependence, besides the one due to the functions ${\hat{h}}_{r_{i}p_{0},\mu_{i}}$, is completely captured by the overall factor of $\tau_{2}^{-2}$. This implies that the generating series $h_{rp_{0},\mu }$ are actually *quasi-modular* forms, which can be constructed as polynomials in the Eisenstein series $E_{2}\left(\tau \right)$ (see Example 6) with coefficients given by usual modular forms. For example, for r=2, $h_{p_{0},\mu }^{\left(1,1\right)}$ is holomorphic because ${\hat{h}}_{p_{0},\mu }=h_{p_{0},\mu }$ and the non-holomorphic dependence of the completion can be captured by ${\hat{E}}_{2}=E_{2}-\frac{3}{\pi \tau_{2}}$. This is equivalent to the statement that

(104) $$h_{2p_{0},\mu }\left(\tau \right)=h_{2p_{0},\mu }^{\left(0\right)}\left(\tau \right)-\frac{E_{2}\left(\tau \right)}{24}\;h_{p_{0},\mu }^{\left(1,1\right)}\left(\tau \right),$$

where $h_{2p_{0},\mu }^{\left(0\right)}$ is a holomorphic modular form. A description in terms of quasi-modular forms is known to hold, for example, for elliptically fibered CYs [106,107,108,109] and their geometric data turn out to perfectly fit the framework presented here and implied by the condition (97) [110].

### 6.2. Non-Compact Calabi–Yau

Next, we show what happens if one takes a non-compact CY threefold. This extension is important because, on the one hand, it can serve as a test-ground for the results presented in Section 5 since, in contrast to the compact case, there are various powerful techniques to compute BPS indices and other topological invariants for non-compact CYs (see, e.g., [111,112,113,114,115,116]), while, on the other hand, it can still tell us something new. This is particularly important from the physical viewpoint because the non-compact case typically provides a geometric realization of supersymmetric gauge theories, which are of great interest [117,118].

A non-compact CY can be obtained as the so-called *local limit* of a compact CY where one zooms in on the region near some singularity in the moduli space corresponding to shrinking of one or several cycles. Let us recall a description of this limit from [92], which puts it in the same framework that was used above. As we will see, the local limit fits the degenerate case considered in Section 6.1.1.

Instead of specifying either a set of shrinking 4-cycles or 2-cycles, let us start from a set of $n_{\infty }$ linearly independent vectors $v_{A}^{a}$ belonging to the closure of the Kähler cone of Y, where the index *A* labels different vectors and takes $n_{\infty }$ values. Given these vectors, we define a set of matrices

(105) $$\kappa_{A,ab}=\kappa_{abc}v_{A}^{c}.$$

We assume that the vectors $v_{A}^{a}$ are chosen so that the matrices $\kappa_{A}$ have a non-trivial common kernel of dimension $n_{0}$, which in particular implies that all $v_{A}^{a}$ must belong to the boundary of the Kähler cone. We denote a basis of this kernel by $v_{I}^{a}$. Obviously, these vectors satisfy

(106) $$\kappa_{A,ab}\;v_{I}^{b}=0$$

for any *A* and *I*. We also assume that the two sets, $v_{A}^{a}$ and $v_{I}^{a}$, are linearly independent and complete them to a basis in $H_{2}\left(Y,R\right)$ by providing an additional set of $b_{2}-n_{\infty }-n_{0}\equiv n_{\mathrm{fr}}$ vectors $v_{X}^{a}$. This allows to expand the Kähler moduli in the new basis

(107) $$t^{a}=v_{A}^{a}\;{\hat{t}}^{A}+v_{X}^{a}\;{\hat{t}}^{X}+v_{I}^{a}\;{\hat{t}}^{I}\equiv v_{b}^{a}\;{\hat{t}}^{b},$$

where we combined three indices *A*, *X* and *I* into one index *b*. Then the local limit is defined by taking the moduli ${\hat{t}}^{A}$ to scale to infinity but keeping ${\hat{t}}^{X}$ and ${\hat{t}}^{I}$ finite. It is important that this definition does not depend on the choice of $v_{X}^{a}$ because changing $v_{X}^{a}$ in (107) can at most shift ${\hat{t}}^{A}$ and ${\hat{t}}^{I}$ by a combination of ${\hat{t}}^{X}$, which does not affect the split between growing and finite variables. In the dual gauge theory, ${\hat{t}}^{I}$ become dynamical Coulomb branch moduli, ${\hat{t}}^{X}$ turn into physical parameters such as masses and the gauge coupling, and ${\hat{t}}^{A}$ drop out from the theory.

By computing the volumes of divisors and curves in the rotated basis, ${\hat{D}}_{a}=v_{a}^{b}D_{b}$ and ${\hat{C}}^{a}={\left(v^{-1}\right)}_{b}^{a}C^{b}$, it is easy to see that in the local limit defined above, the volumes of ${\hat{D}}_{A}$, ${\hat{D}}_{X}$ and ${\hat{C}}^{A}$ grow, while the volumes of ${\hat{D}}_{I}$, ${\hat{C}}^{X}$ and ${\hat{C}}^{I}$ remain finite. (Or, if one divides the Kähler moduli by some physical scale that grows as ${\hat{t}}^{A}$ in the local limit, ${\hat{D}}_{A}$, ${\hat{D}}_{X}$ and ${\hat{C}}^{A}$ stay finite, while ${\hat{D}}_{I}$, ${\hat{C}}^{X}$ and ${\hat{C}}^{I}$ shrink. Thus, the definition of the local limit is equivalent to specifying either the set of shrinking divisors ${\hat{D}}_{I}$ or the set of shrinking curves ${\hat{C}}^{X},{\hat{C}}^{I}$). Therefore, only the D4-branes wrapping ${\hat{D}}_{I}$ and the D2-branes wrapping ${\hat{C}}^{X},{\hat{C}}^{I}$ survive in the limit. In other words, we have access only to the magnetic charges $p^{a}$ that are linear combinations of $v_{I}^{a}$. The associated quadratic forms $\kappa_{I,ab}=\kappa_{abc}v_{I}^{c}$ are degenerate because $v_{A}^{a}$ are their null eigenvectors due to (106). Hence, we fall into the case described in the previous subsection where $rank\left(\kappa_{ab}\right)<b_{2}$ but $p^{a}$ is not a null eigenvector. In our case $rank\left(\kappa_{ab}\right)\leq b_{2}-n_{\infty }$. Note that the curves that could be wrapped by D2-branes satisfy the orthogonality relation

(108) $${\hat{C}}^{X},{\hat{C}}^{I}\cap {\hat{D}}_{A}=0.$$

It provides a physical interpretation of the reduction of the charge lattice (98) given in this case by the condition $v_{I}^{a}q_{a}=0$, which is equivalent to (108).

Since $p^{a}$ is not a null eigenvector of the quadratic form that it defines, in general, the expression (72) for modular completions is not simplified. The only change induced by the local limit is the reduction in charge lattices: magnetic charges should be linear combinations of $v_{I}^{a}$ and electric charges should be orthogonal to all null eigenvectors of the associate quadratic forms, which also affects the modular weight determined now by the rank of the electric charge lattice as in (99).

**Example** **9.** *Elliptically fibered CY [28].*

Let us consider a smooth elliptic fibration π:Y→S with a single section σ over a compact, smooth almost-Fano base *S*. For all smooth elliptic fibrations, a basis of $H^{1,1}\left(Y\right)$ generating the Kähler cone is given by $\left\{\omega_{e},\pi^{*}\omega_{\alpha }\right\}$, $\alpha =1,\ldots ,h^{1,1}\left(S\right)$, where

(109) $$\omega_{e}=\sigma +\pi^{*}c_{1}\left(S\right)$$

and $\omega_{\alpha }$ are the generators of the Kähler cone on the base. We denote the corresponding basis of dual divisors by $\left\{D_{e},D_{\alpha }\right\}$. The divisor $D_{e}$ is dual to the elliptic fiber curve E in the sense that it does not intersect any curve in *S* and obeys $D_{e}\cap E=1$. In this basis, the triple intersection numbers of Y can be shown to be

(110) $$\kappa_{\alpha \beta \gamma }=0,\;\kappa_{e\;\alpha \beta }=C_{\alpha \beta },\;\kappa_{ee\;\alpha }=C_{\alpha \beta }c_{1}^{\beta },\;\kappa_{eee}=C_{\alpha \beta }c_{1}^{\alpha }c_{1}^{\beta },$$

where

(111) $$C_{\alpha \beta }=\int_{S}\omega_{\alpha }\wedge \omega_{\beta },\;c_{1}\left(S\right)=c_{1}^{\alpha }\;\omega_{\alpha },$$

while the components of the second Chern class $c_{2}\left(TY\right)$ are

(112) $$c_{2,e}=11C_{\alpha \beta }c_{1}^{\alpha }c_{1}^{\beta }+\chi \left(S\right),\;c_{2,\alpha }=12\;C_{\alpha \beta }c_{1}^{\beta }.$$

A crucial property of the intersection numbers (110) is that the matrix $\kappa_{eab}$ is degenerate, i.e., its determinant vanishes. This suggests that the vector $v_{1}^{a}=\delta_{e}^{a}$, playing the role of $v_{A}^{a}$ in (105), defines a non-trivial local limit. The kernel of $\kappa_{eab}$ is one-dimensional and described by the vector

(113) $$p_{0}^{a}=\left(1,-c_{1}^{\alpha }\right),$$

playing the role of $v_{I}^{a}$ in (106). The corresponding shrinking divisor ${\hat{D}}_{0}=p_{0}^{a}D_{a}$ is nothing but the base of the elliptic fibration

(114) $${\hat{D}}_{0}=D_{e}-c_{1}^{\alpha }D_{\alpha }=S.$$

It is not an ample divisor, as some of the coefficients of the charge vector (113) are negative, but it is effective. We observe that in the local limit defined by the vector $v_{1}^{a}$, one obtains a non-compact CY given by the total space $\mathrm{Tot}(K_{S})$ of the canonical bundle over the surface *S*. In this limit, all magnetic charges should be multiples of (113), $p^{a}=rp_{0}^{a}$. The associated quadratic forms are given by

(115) $$\kappa_{ab}=\kappa_{abc}p^{c}=r\left(\begin{array}{cc} 0 & 0\\ 0 & C_{\alpha \beta } \end{array}\right).$$

They are all degenerate along the fiber direction described by $v_{1}^{a}$, in agreement with the fact that the divisor *S* is not ample. Since $\mathrm{rank}\left(\kappa_{ab}\right)=b_{2}\left(S\right)$, one finds that the generating functions $h_{p,\mu }$ are higher depth mock modular forms of weight $-\frac{1}{2}\;b_{2}\left(S\right)-1$. Note that it is different from the weight in the compact case because $b_{2}\left(S\right)\neq b_{2}\left(Y\right)$. Since all magnetic charges are collinear, the results presented in Section 5.3.1, including the holomorphic anomaly Equation (95) for the partition function, apply to this example.

As mentioned above, string theory on non-compact CYs can often be reinterpreted as a supersymmetric gauge theory. As a result, BPS indices can also acquire a new interpretation. In particular, in the above example of the non-compact CY given by $\mathrm{Tot}(K_{S})$, the DT invariants counting D4-branes wrapped *r* times around the surface *S* are expected to coincide [101,119,120] with the Vafa–Witten invariants with gauge group U(r) on *S* [54]. In Section 8.2 we show how the formula for the completion ${\hat{h}}_{p,\mu }$ allows to find the generating series of these VW invariants for arbitrary rank *r* for various rational surfaces.

### 6.3. Refinement

Our third extension is the inclusion of a refinement parameter. At a formal level, it is done by simply replacing the sign factor ${\left(-1\right)}^{2J_{3}}$ in the definition of the BPS index, where $J_{3}$ generates rotations around a fixed axis in $R^{3}$, by the factor ${\left(-y\right)}^{2J_{3}}$, where $y=e^{2\pi iz}$ is a new (in general complex) parameter. Physically, the refinement corresponds to switching on the Ω-background [31,32], while mathematically, it gives access to Betti numbers of moduli spaces of stable objects, in contrast to the unrefined DT invariants computing only their Euler characteristic. More precisely, the *refined BPS indices* can be written as Poincaré polynomials

(116) $$\Omega \left(\gamma ,y\right)=\sum_{p=0}^{2d}{\left(-y\right)}^{p-d}\;b_{p}\left(M_{\gamma }\right),$$

where $M_{\gamma }$ is the moduli space of coherent sheaves of charge γ and *d* is its complex dimension. As in (59), we also introduce their rational counterparts [84]

(117) $$\bar{\Omega }\left(\gamma ,y\right)=\sum_{m|\gamma }\frac{y-1/y}{m(y^{m}-1/y^{m})}\;\Omega \left(\gamma /m,y^{m}\right),$$

which simplify the well-known wall-crossing relations satisfied by the refined indices [78,84,85]. We use them to define the generating functions

(118) $$h_{p,\mu }^{\mathrm{ref}}\left(\tau ,z\right)=\sum_{{\hat{q}}_{0}\leq {\hat{q}}_{0}^{\max}}\frac{{\bar{\Omega }}_{p,\mu }\left({\hat{q}}_{0},y\right)}{y-y^{-1}}\;e^{-2\pi i{\hat{q}}_{0}\tau },$$

where, as in Section 4.3, we restricted our attention to D4-D2-D0 bound states, specified the moduli to be at the large volume attractor point, and used the spectral flow invariance to reduce the dependence on charges. An important new feature of (118) is the presence of the denominator, which generates a singularity in the unrefined limit y→1. While its inclusion looks artificial, it turns out to be indispensable for $h_{p,\mu }^{\mathrm{ref}}$ to have nice modular properties.

In [28] it has been shown that the construction of the modular completion ${\hat{h}}_{p,\mu }$ presented in Section 5.3 and encoding the modular anomaly of the generating series $h_{p,\mu }\left(\tau \right)$ of the unrefined BPS indices, has a natural generalization to the refined case. According to this more general construction, the generating series $h_{p,\mu }^{\mathrm{ref}}\left(\tau ,z\right)$ (118) of the refined BPS indices are *higher depth mock Jacobi forms* where the role of the elliptic argument is played by the refinement parameter *z*. Thus, the refinement parameter must transform under SL(2,Z) as z↦z/(cτ+d) together with the modular parameter τ.

A formula for the refined completion takes exactly the same form as (72),

(119) $${\hat{h}}_{p,\mu }^{\mathrm{ref}}\left(\tau ,\bar{\tau },z\right)=h_{p,\mu }^{\mathrm{ref}}\left(\tau ,z\right)+\sum_{n=2}^{r}\sum_{\sum_{i=1}^{n}p_{i}=p}\sum_{\mu }R_{\mu ,\mu }^{(p)\mathrm{ref}}\left(\tau ,\bar{\tau },z\right)\prod_{i=1}^{n}h_{p_{i},\mu_{i}}^{\mathrm{ref}}\left(\tau ,z\right),$$

but with the coefficients given now by

(120) Rμ,μ(p)ref(τ,τ¯,z)=∑∑i=1nqi=μ+12pqi∈Λpi+μi+12piSym(−y)∑i<jγijRnref(γ^;τ2,β)eπiτQn(γ^),

where we set z=α−τβ with α,β∈R. The main difference here, besides the appearance of a power of *y*, lies in the form of the functions $R_{n}^{\mathrm{ref}}$. They turn out to be much simpler than their unrefined version $R_{n}$. In particular, while $R_{n}$ involve a sum over two types of trees weighted by generalized error functions and their derivatives, $R_{n}^{\mathrm{ref}}$ are defined using only one type of trees (or even without them at all!) and no derivatives. More precisely, the difference is hidden in the functions $E_{n}^{\left(\mathrm{ref}\right)}\left(\hat{\gamma };\tau_{2},\beta \right)$, a refined analogue of $E_{n}\left(\hat{\gamma };\tau_{2}\right)$ (80). Although they depend on the additional parameter β, they are actually much simpler than $E_{n}$ because in their definition there is not any sum over trees. They simply coincide with the generalized error functions evaluated at appropriate variables (cf. (42)):

(121) $$E_{n}^{\left(\mathrm{ref}\right)}\left(\hat{\gamma };\tau_{2},\beta \right)=\Phi_{n-1}^{E}\left(\left\{v_{\ell }\right\};\sqrt{2\tau_{2}}\;\left(q+\beta \theta \right)\right),$$

where

(122) $$v_{\ell }=\sum_{i=1}^{\ell }\sum_{j=\ell +1}^{n}v_{ij},\;\theta =\sum_{i<j}v_{ij},$$

while other notations are the same as in Section 5.3. Note that $v_{\ell }$ can be thought of as the vectors $v_{e}$ (75) assigned to edges of the simplest unrooted linear tree $T_{\mathrm{lin}}=•\;\text{—}\;•\;\text{–}\cdots \text{–}\;•\;\text{—}\;•\;$. The functions $E_{n}^{\left(\mathrm{ref}\right)}$ have a canonical decomposition similar to (81), $E_{n}^{\left(\mathrm{ref}\right)}=E_{n}^{(0)\mathrm{ref}}+E_{n}^{(+)\mathrm{ref}}$, where $E_{n}^{(0)\mathrm{ref}}$ is the large $\tau_{2}$ limit of $E_{n}^{\left(\mathrm{ref}\right)}$ evaluated at β=0:

(123) $$E_{n}^{(0)\mathrm{ref}}\left(\hat{\gamma }\right)\equiv \lim_{\tau_{2}\to \infty }E_{n}^{\left(\mathrm{ref}\right)}\left(\left\{{\hat{\gamma }}_{i}\right\};\tau_{2},0\right)=S_{T_{\mathrm{lin}}}\left(\hat{\gamma }\right).$$

Here $S_{T}$ is defined in (84) and for the linear tree the coefficients $e_{T}$ can be computed to be $e_{T_{\mathrm{lin}}}\equiv e_{n-1}=\frac{1}{n}\;\delta_{n-1}^{\left(2\right)}$ with *n* being the number of vertices. Finally, the formula for $R_{n}^{\mathrm{ref}}$ looks exactly as (87):

(124) $$R_{n}^{\mathrm{ref}}\left(\hat{\gamma };\tau_{2},\beta \right)=\frac{1}{2^{n-1}}\sum_{T\in T_{n}^{S}}{\left(-1\right)}^{n_{T}-1}E_{v_{0}}^{(+)\mathrm{ref}}\prod_{v\in V_{T}\setminus \left\{v_{0}\right\}}E_{v}^{(0)\mathrm{ref}}.$$

The claim is that the functions (119) transform as vector-valued Jacobi forms of weight and index given by

(125) $$w=-\frac{1}{2}\;b_{2},\;m\left(p\right)=-\chi \left(O_{D_{p}}\right),$$

where $\chi (O_{D_{p}})$ is the arithmetic genus (91), and with the same multiplier system (90) as in the unrefined case. Furthermore, in the unrefined limit, after multiplication by $y-y^{-1}$ to cancel this factor in (118), the refined completion reduces to (72). Namely,

(126) $${\hat{h}}_{p,\mu }\left(\tau ,\bar{\tau }\right)=\lim_{y\to 1}\left[\left(y-y^{-1}\right)\;{\hat{h}}_{p,\mu }^{\mathrm{ref}}\left(\tau ,\bar{\tau },z\right)\right].$$

This fact follows from a similar very non-trivial property of the coefficients $R_{\mu ,\mu }^{(p)\mathrm{ref}}$:

(127) $$R_{\mu ,\mu }^{\left(p\right)}\left(\tau ,\bar{\tau }\right)=\lim_{y\to 1}\left[{\left(y-y^{-1}\right)}^{1-n}\;R_{\mu ,\mu }^{(p)\mathrm{ref}}\left(\tau ,\bar{\tau },z\right)\right],$$

which ensures the cancellation of the poles of the refined generating functions appearing on the r.h.s. of (119). It also explains the appearance of the derivatives of the generalized error functions in the unrefined construction (see (77)) as a consequence of applying the L’Hôpital’s rule to evaluate the limit in (127). Furthermore, it is worth mentioning that the results presented in Section 5.3.1 have originally been proven in the refined case and then followed by taking the limit (126) [28].

In fact, it was recently noticed [121] that in the one-modulus case, i.e., $b_{2}=1$, for n=3 and 4, the sum over Schröder trees in (124), upon substitution of the expression (A7) of the generalized error functions $\Phi_{n}^{E}$ in terms of their complementary counterparts ${\hat{\Phi }}_{n}^{M}$ (A6), results in a huge cancellation, leaving a single term supplemented only by contributions involving Kronecker deltas of the type appearing in (84). This observation suggests the following

**Conjecture** **1.** *Let us introduce the functions*

(128) $$G_{n}\left(\hat{\gamma };\tau_{2},\beta \right)={\hat{\Phi }}_{n-1}^{M}\left(\left\{v_{\ell }\right\};\sqrt{2\tau_{2}}\;\left(q+\beta \theta \right),\sqrt{2\tau_{2}}\;\beta \theta \right).$$

*Then in the one-modulus case, the functions $R_{n}^{\mathrm{ref}}$ determining the coefficients (120) are given by*

(129) $$R_{n}^{\mathrm{ref}}\left(\hat{\gamma };\tau_{2},\beta \right)=\frac{1}{2^{n-1}}\;\sum_{J\subseteq Z_{n-1}}b_{\left|J\right|}\;\delta_{J}\;G_{n-|J|}\left({\hat{\gamma }}_{J};\tau_{2},\beta \right),$$

*where*

(130) $$b_{n-2}=\frac{2^{n}\left(2^{n}-1\right)}{n!}\;B_{n},\;\delta_{J}=\prod_{k\in J}\delta_{\Gamma_{k}},\;\Gamma_{k}=\sum_{i=1}^{k}\sum_{j=k+1}^{n}\gamma_{ij},$$

*with $B_{n}$ being the Bernoulli number, and ${\hat{\gamma }}_{J}$ is obtained from $\hat{\gamma }$ by combining each ${\hat{\gamma }}_{i}$, i∈J, with the next charge. For example, ${\left({\hat{\gamma }}_{1},{\hat{\gamma }}_{2},{\hat{\gamma }}_{3},{\hat{\gamma }}_{4},{\hat{\gamma }}_{5}\right)}_{\left\{1,2,4\right\}}=\left({\hat{\gamma }}_{1}+{\hat{\gamma }}_{2}+{\hat{\gamma }}_{3},{\hat{\gamma }}_{4}+{\hat{\gamma }}_{5}\right)$.*

**Remark** **3.** *The coefficients $b_{n}$ have been introduced in [26] and are the Taylor series coefficients of tanh(x)/x. Here they arise as*

$$b_{n}=\sum_{T\in T_{n+1}^{\mathrm{odd}}}{\left(-1\right)}^{n_{T}-1}\prod_{v\in V_{T}}\frac{1}{k_{v}}\;,$$

*where $T_{n}^{\mathrm{odd}}$ denotes planar rooted trees with n vertices for which the number of children $k_{v}$ at vertex v is odd and ≥3, and the factors $1/k_{v}$ can be recognized as the coefficients $e_{k_{v}-1}$ introduced below (123).*

This conjecture has not been proven yet in full generality. If correct, it further simplifies the anomaly by expressing each term in (119) as an iterated integral of depth n−1. Since the anomaly in the unrefined case can be obtained by taking the limit (127) of the refined coefficients, for $b_{2}=1$, the construction presented in Section 5.3 is also expected to have a simpler version in terms of the complementary generalized error functions ${\hat{\Phi }}_{n}^{M}$.

Finally, it was argued in [28] that, if the quadratic form is degenerate, as it happens in the case of an elliptically fibered CY or its local limit (Example 9), this affects not only the weight of the refined generating functions but also their index. The previous expressions (125) are then replaced by

(131) $$w\left(p\right)=-\frac{1}{2}\;\mathrm{rank}\left(\Lambda_{p}\right),\;m\left(p\right)=-\chi \left(O_{D_{p}}\right)-\lambda_{a}p^{a},$$

where the last term should be an integer. Its precise value in the generic case and its origin remain unclear. In Section 8.2, this term will be needed to get the correct value of the index of the generating functions of refined VW invariants.

We observe that the refined BPS indices appear to possess very similar modular properties to the unrefined ones and even simplify the description of the corresponding modular anomaly. However, in the case of a compact CY threefold, there is a problem: it is not clear whether the refined BPS indices can actually be well-defined. More precisely, there seems to be no natural deformation invariant way of defining them. In physics terms, this means that their naive definition is not protected by supersymmetry and they may change under the variation of hypermultiplet moduli. In a drastic contrast, in the case of non-compact CYs, it is possible to define protected refined indices due to the existence of a certain $C^{\times }$ action carried by the moduli space of semi-stable sheaves, corresponding to an additional SU(2) R-symmetry in the dual supersymmetric gauge theories. This is achieved by changing the factor ${\left(-y\right)}^{2J_{3}}$ by ${\left(-1\right)}^{2J_{3}}y^{2(J_{3}+I)}$ where *I* is the generator of the additional symmetry [122]. (Recently, in [123] this fact was used to define the refined BPS indices for elliptically fibered CY threefolds through the gauge theories emerging in various local limits).

Thus, even if the compact case remains problematic, there are two possible ways to use the construction presented in this subsection:

- As a description of modular properties of the generating functions of refined BPS indices on non-compact CYs (or in any other case, like in [123], where these indices can be well-defined);
- As a useful trick to compute modular completions in the unrefined case.

Below we explain an additional structure associated with the refined construction, which suggests that it is not just a mere trick but reveals something fundamental even in the compact case.

#### 6.3.1. Non-Commutative Structure

An important difference between the unrefined and refined constructions is that the former was derived (following the steps sketched in Section 5.2), whereas the latter was simply guessed. One of the reasons for this was explained above: this is the lack of a satisfactory definition of refined BPS indices for compact CYs and, as a result, the lack of understanding of the implications on them of string dualities. Even the fate of S-duality under the refinement is not clear.

Nevertheless, in [28] it was shown that it is possible to revert the logic explained in Section 5.2 and, starting from (119), to reconstruct a function on the moduli space M whose modularity is equivalent to the expression for ${\hat{h}}_{p,\mu }^{\mathrm{ref}}$. This function $G^{\left(\mathrm{ref}\right)}$ is supposed to be a refined analogue of the instanton generating potential G (66). Although its geometric meaning remains unclear, its existence hints that S-duality is preserved by the refinement.

Moreover, it strongly suggests that the refinement makes the moduli space M *non-commutative*! On the one hand, this is somewhat expected from previous studies of refined indices [122,124]. On the other hand, it is remarkable that the non-commutativity appears as an indispensable ingredient in the definition of $G^{\left(\mathrm{ref}\right)}$. It manifests through the following *modular invariant* star product defined on functions on the moduli space:

(132) $$f★g=f\exp\left[\frac{1}{2\pi i}\left({\overset{\leftarrow }{D}}_{\;\;a}{\vec{\partial }}_{\;{\tilde{c}}_{a}}-{\overset{\leftarrow }{\partial }}_{\;{\tilde{c}}_{a}}{\vec{D}}_{\;\;a}\right)\right]g,$$

where

(133) $$D_{a}=\alpha \partial_{c^{a}}+\beta \partial_{b^{a}}=z\partial_{v^{a}}+\bar{z}\partial_{{\bar{v}}^{a}}$$

with $v^{a}=c^{a}-\tau b^{a}$. The modular invariance is due to the fact that *z* and $v^{a}$ both transform as elliptic variables, whereas $\partial_{{\tilde{c}}_{a}}$ is modular invariant. One can show that with respect to this star product the classical Darboux coordinates (65) satisfy

(134) $$X_{\gamma_{1}}^{\mathrm{cl}}\left(z_{1}\right)★X_{\gamma_{2}}^{\mathrm{cl}}\left(z_{2}\right)=y^{\gamma_{12}+\frac{\beta }{2}\;\left(p_{1}p_{2}\left(p_{1}+p_{2}\right)\right)}{\left(y\bar{y}\right)}^{-i\left(p_{1}p_{2}t\right)\left(z_{1}-z_{2}\right)}X_{\gamma_{1}}^{\mathrm{cl}}\left(z_{1}\right)X_{\gamma_{2}}^{\mathrm{cl}}\left(z_{2}\right).$$

This non-commutativity relation might look unusual, but this is because we allowed the refinement parameter *z* to have a non-vanishing imaginary part. If it vanishes, i.e., β=0 so that *y* is a pure phase, the only remaining factor in (134) is $y^{\gamma_{12}}$, as in [122,124].

Using the star product (132), we define refined Darboux coordinates as solutions of the following integral equation (cf. (63))

(135) $$X_{\gamma }^{\left(\mathrm{ref}\right)}=X_{\gamma }^{\mathrm{cl}}★\left(1+J_{\gamma }^{\left(\mathrm{ref}\right)}\right),\;J_{\gamma }^{\left(\mathrm{ref}\right)}\left(z\right)=\sum_{\gamma^{\prime }\in \Gamma_{+}}\bar{\Omega }\left(\gamma ,y\right)\;\int_{\ell_{\gamma^{\prime }}}dz^{\prime }\;K_{\gamma \gamma^{\prime }}^{\left(\mathrm{ref}\right)}\left(z,z^{\prime }\right)\;X_{\gamma^{\prime }}^{\left(\mathrm{ref}\right)}\left(z^{\prime }\right),$$

where the integration kernel is given by

(136) $$K_{\gamma_{1}\gamma_{2}}^{\left(\mathrm{ref}\right)}\left(z_{1},z_{2}\right)=\frac{i}{2\pi }\;\frac{y^{-\beta m(p)}}{y-y^{-1}}\;\frac{1}{z_{1}-z_{2}}$$

and m(p) is the index (125). Then the refined instanton generating potential can be shown to have the following integral representation

(137) $$G^{\left(\mathrm{ref}\right)}=\frac{1}{4\pi^{2}}\;\frac{y^{-\beta m(p)}}{y-y^{-1}}\sum_{\gamma \in \Gamma_{+}}\bar{\Omega }\left(\gamma ,y\right)\int_{\ell_{\gamma }}dz\;X_{\gamma }^{\left(\mathrm{ref}\right)}\left(z\right).$$

It is a Jacobi form of weight $\left(-\frac{1}{2}\;,\;\frac{1}{2}\right)$ and index 0 provided ${\hat{h}}_{p,\mu }^{\mathrm{ref}}$ (119) have the modular properties specified in (125). Comparing to (66), one again observes that the refined version is simpler as it is given by a single integral in contrast to the unrefined version, which involves a double integral.

One can wonder why the integral Equation (135) has such an unusual form, which has nothing to do with TBA-like equations. In fact, it does not have the unrefined limit due to the pole in the kernel (136), just as there is no such limit for the refined Darboux coordinates $X_{\gamma }^{\left(\mathrm{ref}\right)}$. Nevertheless, the limit exists for $\left(y-y^{-1}\right)G^{\left(\mathrm{ref}\right)}$ where it reproduces G. The situation was clarified recently in [125]. It turns out that one can introduce another version of refined Darboux coordinates defined through $X_{\gamma }^{\left(\mathrm{ref}\right)}$ by

(138) $${\hat{X}}_{\gamma }^{\left(\mathrm{ref}\right)}={\left(1+J_{\gamma }^{\left(\mathrm{ref}\right)}\right)}_{★}^{-1}★X_{\gamma }^{\left(\mathrm{ref}\right)}={\left(1+J_{\gamma }^{\left(\mathrm{ref}\right)}\right)}_{★}^{-1}★X_{\gamma }^{\mathrm{cl}}★\left(1+J_{\gamma }^{\left(\mathrm{ref}\right)}\right),$$

where the star index means that ${\left(1+x\right)}_{★}^{-1}=\sum_{n=1}^{\infty }{\left(-1\right)}^{n}x★\cdots ★x$. One can show that the new coordinates do have the unrefined limit where they reduce to $X_{\gamma }$. Furthermore, they satisfy the standard refined wall-crossing relations [78] and, for $z_{1}=z_{2}$, the commutation relations (134). Thus, these are ${\hat{X}}_{\gamma }^{\left(\mathrm{ref}\right)}$, rather than $X_{\gamma }^{\left(\mathrm{ref}\right)}$, which should be considered as a refined version of $X_{\gamma }$ and as a solution of the quantum Riemann–Hilbert problem introduced in [126] and studied in [127,128,129].

Since $X_{\gamma }$ are the Darboux coordinates on the twistor space over M, one can view ${\hat{X}}_{\gamma }^{\left(\mathrm{ref}\right)}$ as Darboux coordinates on a quantization of this twistor space. Although a theory of such non-commutative spaces has not been developed yet, a small step towards it has been performed in [130] where it was shown how one can define a *quantized contact structure*. Thus, one can hope that the above construction opens a door into a potentially rich and still poorly explored topic of quantum twistor spaces.

## 7. Solution of the Modular Anomaly

The expression for the modular completion (72) (or its refined version (119)) can be seen as an iterative system of modular anomaly equations on the generating functions $h_{p,\mu }$. Of course, these equations cannot fix $h_{p,\mu }$ uniquely but only up to addition of a holomorphic modular form, which is not “seen” by the modular anomaly. To fix the modular ambiguity, one should provide additional information. For example, as explained in Section 2, this can be information about the coefficients of the polar terms of $h_{p,\mu }$.

Thus, the equations (72) can be used to find the generating functions, but one should follow a two step strategy:

1. Find *any* mock modular form $h_{p,\mu }^{\left(\mathrm{an}\right)}$ having the modular anomaly described by (72);
2. Represent
  (139) $$h_{p,\mu }=h_{p,\mu }^{\left(\mathrm{an}\right)}+h_{p,\mu }^{\left(0\right)},$$
  where $h_{r,\mu }^{\left(0\right)}$ is a modular form, and find the second term by computing the polar terms.

Of course, for $p^{a}$ corresponding to irreducible divisors, the first step is not necessary as the generating function is not anomalous.

The computation of polar terms is, in general, a highly non-trivial problem. We show how it can be systematically approached in Section 8.1 in the case of one-parameter CY threefolds, i.e., when $b_{2}=1$. However, it seems impossible to give any general formulas for the polar coefficients, so that they have to be computed example by example. And even in the one-parameter case, the results remain quite limited. In contrast, as we will see, it is possible to solve the modular anomaly equations (72), at least in the same one-parameter case, in full generality. Given this situation, we postpone the second step of the above procedure and concentrate in this section on the first one.

### 7.1. Anomalous Coefficients

An immediate problem arising when one tries to solve (72) to get $h_{p,\mu }^{\left(\mathrm{an}\right)}$ is that the r.h.s. of this equation depends on the *full* generating functions $h_{p_{i},\mu_{i}}$ of the constituents and hence on all functions $h_{p_{i},\mu_{i}}^{\left(0\right)}$ that remain unknown because we decided to fix them at a later stage. In such situation, the best we can do is to find $h_{p,\mu }^{\left(\mathrm{an}\right)}$ in a form parametrized by $h_{p_{i},\mu_{i}}^{\left(0\right)}$. It is clear that the dependence on these functions must be polynomial and in each monomial the charges $p_{i}^{a}$ must sum up to $p^{a}$. This brings us to the following ansatz

(140) $$h_{p,\mu }^{\left(\mathrm{an}\right)}\left(\tau \right)=\sum_{n=2}^{r}\sum_{\sum_{i=1}^{n}p_{i}=p}\sum_{\mu }g_{\mu ,\mu }^{\left(p\right)}\left(\tau \right)\prod_{i=1}^{n}h_{p_{i},\mu_{i}}^{\left(0\right)}\left(\tau \right).$$

Note that one can write a similar formula for the full generating function $h_{p,\mu }$ if one starts the sum from n=1 and sets by definition $g_{\mu ,\mu^{\prime }}^{\left(p\right)}=\delta_{\mu ,\mu^{\prime }}$. The other coefficients $g_{\mu ,\mu }^{\left(p\right)}$ with n≥2, where *n* is the length of the tuples p and μ, are the functions to be found. To do this, we need to know the constraints that they satisfy and that can be derived by plugging the ansatz (140) into (72). In [40], the following result has been proven (in fact, in [40] Theorem 2 has been proven only in the one-modulus case, but it is easy to see that exactly the same proof applies to the generic case):

**Theorem** **2.** *Let $h_{p,\mu }^{\left(0\right)}$ be a set of holomorphic modular forms. Then $h_{p,\mu }$ is a depth r−1 modular form whose completion has the form (72) provided $g_{\mu ,\mu }^{\left(p\right)}$ are depth n−1 mock modular forms whose completions satisfy*

(141) $${\hat{g}}_{\mu ,\mu }^{\left(p\right)}=\;\mathrm{Sym}\;\left\{\sum_{m=1}^{n}\sum_{\sum_{k=1}^{m}n_{k}=n}\sum_{\nu }R_{\mu ,\nu }^{\left(s\right)}\prod_{k=1}^{m}g_{\nu_{k},m_{k}}^{\left(p_{k}\right)}\right\},$$

*where*

(142) jk=∑l=1k−1nl,ska=∑i=1nkpjk+ia,pk=(pjk+1a,…,pjk+1a),mk=(μjk+1,…,μjk+1).∑a

**Figure 4:**
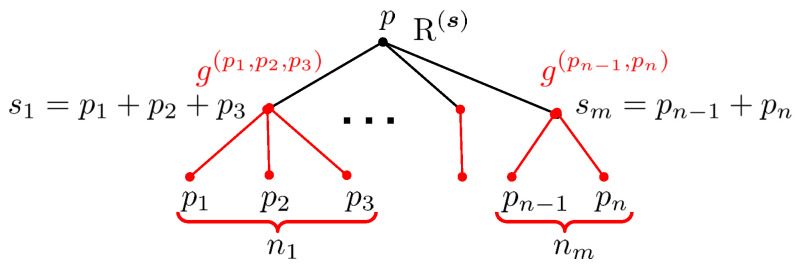
A representation of contributions to the r.h.s. of (141) in terms of rooted trees of depth 2.

Note that while the sets p and μ have *n* elements, the sets s and ν have only m≤n elements. To comprehend the structure of the Equation (141), it might be useful to notice the fact that the sum on its r.h.s. is equivalent to the sum over rooted trees of depth 2 with leaves labeled by charges $p_{i}^{a}$ and other vertices labeled by the sum of the charges of their children. Using this labeling, we assign the function $R_{\mu ,\nu }^{\left(s\right)}$ to the root vertex and the coefficients $g_{\nu_{k},m_{k}}^{\left(p_{k}\right)}$ to the vertices of depth 1 with arguments determined by the charges of their children (see Figure 4 and cf. Figure 3). Then the contribution of a tree is given by the product of the weights of its vertices.

Theorem 2 reformulates the problem of finding $h_{p,\mu }^{\left(\mathrm{an}\right)}$ as the problem of finding the functions $g_{\mu ,\mu }^{\left(p\right)}$, which were called *anomalous coefficients*. It states that they are also higher depth mock modular forms satisfying an iterative system of anomaly equations. Then why is it better than the previous one? The difference between (72) and (141), is that, solving the latter, there is no need to fix the modular ambiguity in $g_{\mu ,\mu }^{\left(p\right)}$. As was emphasized above, *any* solution will suit our purposes because a difference between two solutions can always be absorbed into a redefinition of the modular ambiguities $h_{p,\mu }^{\left(0\right)}$ in the formula for the generating functions ((139) combined with (140)). It is important, however, to take into account that a choice of solution for *n* charges affects all anomaly equations for $g_{\mu ,\mu }^{\left(p\right)}$ with $n^{\prime }>n$ charges. One should remember this when one deals with different systems of solutions to avoid potential inconsistencies.

### 7.2. One-Modulus Case

The problem of finding the anomalous coefficients $g_{\mu ,\mu }^{\left(p\right)}$ has been addressed in the case of compact CY threefolds with one Kähler modulus [40]. Since in this case the D4-brane charge $p^{1}$ is equal to the degree of reducibility of the corresponding divisor *r*, we will use the latter variable to denote magnetic charges. In addition, it is convenient to make a redefinition, which allows us to absorb some annoying sign factors. Namely, let us introduce redefined generating functions

(143) $${\tilde{h}}_{r,\mu }\left(\tau \right)={\left(-1\right)}^{(r-1)\mu }h_{r,\mu -\frac{\kappa r(r-1)}{2}}\left(\tau \right).$$

This leads to two simplifications: (i) the shift of μ replaces the last term in the spectral flow decomposition (57), which in the one-parameter case reads as $\frac{1}{2}\kappa r^{2}$, by a term linear in *r*,

(144) $$q=\kappa r\epsilon +\mu +\frac{1}{2}\;\kappa r;$$

(ii) the sign factor in (143) cancels the sign factor in (73). In particular, due to the first simplification, the condition on the sum over $q_{i}$ in (73) takes the following simple form

(145) $$\kappa \sum_{i=1}^{n}r_{i}\epsilon_{i}=\Delta \mu ,\;\Delta \mu =\mu -\sum_{i=1}^{n}\mu_{i}.$$

Then we introduce redefined anomalous coefficients, for which, to avoid cluttering, we will use the same notation $g_{\mu ,\mu }^{\left(r\right)}$ as before the redefinition. We define them by the same ansatz (140), but with the functions $h_{p,\mu }^{\left(\mathrm{an}\right)}$ and $h_{p,\mu }^{\left(0\right)}$ replaced by their redefined versions. Equivalently, they should satisfy (141) with $R_{\mu ,\nu }^{\left(r\right)}$ replaced by

(146) R˜μ,μ(r)(τ,τ¯)=∑∑i=1nqi=μ+κr/2qi∈κriZ+μi+κri/2SymRn(γ^;τ2)eπiτQn(γ^).

### 7.3. Partial Solutions

If one restricts to n=2, there is a very simple way to solve for $g_{\mu ,\mu_{1},\mu_{2}}^{\left(r_{1},r_{2}\right)}$. From the holomorphic anomaly Equation (A18) specified to $p_{0}=1$, it follows that one can take

(147) $$g_{\mu ,\mu_{1},\mu_{2}}^{\left(r_{1},r_{2}\right)}\left(\tau \right)=r_{0}\delta_{\Delta \mu }^{\left(\kappa r_{0}\right)}G_{\mu_{12}}^{\left(\kappa_{12}\right)}\left(\tau \right),$$

where $r_{0}=\gcd\left(r_{1},r_{2}\right)$, $\kappa_{12}=\kappa r_{12}/2$, $r_{12}$ and $\mu_{12}$ are parameters introduced in Appendix D (in the definition (A17) of $\mu_{12}$, one should drop the term proportional to $p_{0}$, which disappears due to the redefinition (143)), $\mu_{12}$ runs over $2\kappa_{12}$ values, and $G_{\mu }^{\left(\kappa \right)}$ is a vector-valued mock modular form of weight 3/2 with the shadow proportional to the following unary holomorphic theta series

(148) $$\theta_{\mu }^{\left(\kappa \right)}\left(\tau \right)=\sum_{k\in 2\kappa Z+\mu }q^{\frac{k^{2}}{4\kappa }}.$$

Thus, $G_{\mu }^{\left(\kappa \right)}$ is not a mixed but ordinary mock modular form, and the problem reduces to its reconstruction given its shadow. Remarkably, exactly this problem was solved in [16] in the context of N=4 string compactifications, with an additional condition that the mock modular form should have the slowest possible asymptotic growth of its Fourier coefficients. Such functions have been called mock modular forms of optimal growth. Although we do not impose any restrictions on the asymptotic growth, we can take $G_{\mu }^{\left(\kappa \right)}$ to be the solution found in [16] because, as was discussed above, any solution is equally suitable for us. All other solutions would differ just by a pure modular form.

The mock modular forms of optimal growth are determined by a single parameter κ and constructed by acting by certain Hecke-like operators on a set of “seed” mock modular forms $G_{\mu }^{\left(d\right)}$, which have to be introduced for each square-free integer *d* with an even number of prime factors, such as 1, 6, 10, 14, 15, etc. In particular, for d=1, the seed function is given by the generating series of Hurwitz class numbers (see Example 7):

(149) $$G_{\mu }^{\left(1\right)}\left(\tau \right)=H_{\mu }\left(\tau \right).$$

This implies that for all $\kappa_{12}$ given by a power of a prime number, the mock modular form $G_{\mu_{12}}^{\left(\kappa_{12}\right)}$ is generated by the Hurwitz class numbers. For a detailed description of the solution for $G_{\mu_{12}}^{\left(\kappa_{12}\right)}$ in terms of the mock modular forms of optimal growth, we refer to [40].

Another class of anomalous coefficients that can be found almost for free appears for CYs with the intersection number κ=1. It comprises the anomalous coefficients with all charges $r_{i}$ equal to one. A crucial simplification in this case is that one can drop all indices $\mu_{i}$ because they take only $\kappa r_{i}=1$ value. Due to this, the corresponding anomalous coefficients can be denoted simply as $g_{n,\mu }\equiv g_{\mu }^{\left(1,\ldots ,1\right)}$. It turns out that they can be identified with the normalized generating series of U(n) VW invariants on $P^{2}$ (see Section 8.2)

(150) $$g_{n,\mu }\left(\tau \right)=\eta^{3n}\left(\tau \right)\;h_{n,\mu }^{P^{2}}\left(\tau \right),$$

where we used the fact that $h_{1}^{P^{2}}=\eta^{-3}$. More precisely, one can take [40]

(151) $$g_{n,\mu }=3^{1-n}g_{n,\mu }\left(\tau \right).\;$$

Note that for n=2, this choice is consistent with the solution given by the mock modular form of optimal growth, which coincides with (149).

Unfortunately, neither of the above solutions seems to be generalizable to other cases. Therefore, below we present a different construction, which is more complicated, but works for generic charges and parameters. It produces different anomalous coefficients from the ones introduced in this subsection. Therefore, as explained at the end of Section 7.1, if one wants to go beyond n=2 or the very special case $\kappa =r_{i}=1$, even for n=2, one should use the solution constructed in the next subsection and not here.

### 7.4. General Solution

A solution for the anomalous coefficients that works for any *n* and any charges $r_{i}$ can be constructed in terms of indefinite theta series. This is a very natural approach given that the functions $R_{\mu ,\nu }^{\left(r\right)}$, as well as their redefined version ${\tilde{R}}_{\mu ,\mu }^{\left(r\right)}$, determining the modular anomaly of $g_{\mu ,\mu }^{\left(r\right)}$ are themselves such indefinite theta series. (More precisely, they are defined by the quadratic form (74), which is *negative definite* in the one-modulus case. As we will see, this fact leads to additional complications). Although we could just present the final result found in [40] together with its necessary ingredients, we prefer first to explain where it comes from. Otherwise, its rather non-trivial form would look completely mysterious to the reader.

#### 7.4.1. Strategy

The anomalous coefficients must be holomorphic functions. Therefore, if we express them in terms of indefinite theta series, as was explained in Section 3, the kernels of these theta series must be combinations of sign functions. A typical example of such kernel is provided in Theorem 1 and is determined by two sets of vectors of dimension of the lattice. On the other hand, after substitution into (141), the theta series must recombine into a modular form. According to the recipe (41), this means that each product of sign functions should be effectively replaced by the corresponding generalized error function. However, already for n=2 it is easy to see that this is impossible. Indeed, in this case the anomaly equation to be solved takes the simple form

(152) $${\hat{g}}_{\mu ,\mu_{1},\mu_{2}}^{\left(r_{1},r_{2}\right)}\left(\tau ,\bar{\tau }\right)=g_{\mu ,\mu_{1},\mu_{2}}^{\left(r_{1},r_{2}\right)}\left(\tau \right)+{\tilde{R}}_{\mu ,\mu_{1},\mu_{2}}^{\left(r_{1},r_{2}\right)}\left(\tau ,\bar{\tau }\right).$$

The function ${\tilde{R}}_{\mu ,\mu_{1},\mu_{2}}^{\left(r_{1},r_{2}\right)}$ is built of a single complementary error function, whereas the kernel of an indefinite theta series, to make it convergent, should be a linear combination of at least two sign functions. Thus, one sign function can be recombined with ${\tilde{R}}_{\mu ,\mu_{1},\mu_{2}}^{\left(r_{1},r_{2}\right)}$ to produce an error function, while the second sign remains “uncompleted”. Fortunately, this problem can be solved by choosing the vector defining the second sign function to be *null* with respect to the relevant quadratic form. Then, due to the property (A4), the completion is not required. This solution generalizes to any *n*: choosing the kernel to be of the type (37), one set of vectors will be determined by the vectors appearing in the definition of ${\tilde{R}}_{\mu ,\mu_{1},\mu_{2}}^{\left(r_{1},r_{2}\right)}$, while the second set should consist of null vectors.

However, null vectors give rise to other problems. The first one is related to convergence because null vectors spoil the conditions of Theorem 1. On the other hand, as discussed below Theorem 1, they can still be included provided (i) they belong to the relevant lattice, (ii) the theta series includes a non-vanishing elliptic parameter. In our story, the latter can be associated with the refinement parameter *z* (see Section 6.3). Thus, we must switch on the refinement if we want to use indefinite theta series! Of course, in the end, one should take the unrefined limit, which can be non-singular only if the indefinite theta series are combined with some other types of Jacobi forms. In fact, since we are interested only in the behavior near z=0, the elliptic property (27a) of Jacobi forms is not essential and can be abandoned, so that it is sufficient to require that all relevant functions are Jacobi-like forms.

The second problem with null vectors is that they simply do not exist in our lattice just because it is negative definite (see the remark in the beginning of Section 7.4). This problem can be solved by a well-known trick in the theory of mock modular forms (see, e.g., [18]), which is to effectively extend the lattice by multiplying, for example, with Jacobi theta functions (30). Though this trick works, it gives rise to a serious technical complication. It turns out that for a solution on the extended lattice to be reducible to a solution on the original lattice, it should have zero at z=0 of order given by the difference of the dimensions of the two lattices. This property is very difficult to achieve. Fortunately, this issue can be avoided by introducing multiple refinement parameters combined into a vector $z=(z\theta ,\vec{z})$ of dimension of the extended lattice, such that $\left(0,\vec{z}\right)$ is orthogonal to all null vectors used in the construction. The latter condition ensures the decoupling of the auxiliary part of the lattice. As a result, the indefinite theta series we have to deal with are multi-variable (mock) Jacobi forms as in (42). Once they are constructed and combined with proper Jacobi-like forms to ensure the existence of the unrefined limit, they have to be reduced to a solution of the original problem.

Thus, the solution presented below is constructed by performing the following steps:

1. First, one introduces the refinement and looks for vector-valued mock Jacobi-like forms $g_{\mu ,\mu }^{(r)\mathrm{ref}}\left(\tau ,z\right)$ of depth n−1, weight $\frac{1}{2}\left(n-1\right)$, index
  (153) $$m_{r}=-\frac{\kappa }{6}\;\left(r^{3}-\sum_{i=1}^{n}r_{i}^{3}\right),$$
  and the multiplier system specified in [40], Eq.(B.4), satisfying the analogue of the equations (141) with $R_{\mu ,\nu }^{\left(r\right)}$ replaced by ${\tilde{R}}_{\mu ,\mu }^{(r)\mathrm{ref}}$, the redefined version of (120), and having a zero of order n−1 at z=0 to ensure the unrefined limit (see (164) below).
2. Next, one extends the charge lattice so that it possesses a set of null vectors suitable for solving the anomaly equation and associates with the lattice extension a vector of additional refinement parameters satisfying certain orthogonality properties with the null vectors.
  To this end, let us define $\epsilon =\delta_{\kappa -1}$, $d_{r}=4^{\epsilon }\kappa r$, $d_{r}=\sum_{i=1}^{n}d_{r_{i}}$, and introduce $d_{r}$-dimensional vectors $t^{\left(r\right)}$ such that their components are all non-vanishing integers and sum to zero, $\sum_{\alpha =1}^{d_{r}}t_{\alpha }^{\left(r\right)}=0$. Of course, there are plenty of possible choices of such vectors and the following construction does not depend on their concrete form. Given these data, one looks for vector valued multi-variable mock Jacobi-like forms ${\overset{ˇ}{g}}_{\mu ,\mu }^{(r)\mathrm{ref}}\left(\tau ,z,z\right)$ depending on n+1 refinement parameters (z,z) where $z=(z_{1},\ldots ,z_{n})$ and satisfying a new anomaly equation:
  (154) gˇ^gˇμ,μ(r)ref(τ,z,z)=Sym∑m=1n∑∑k=1mnk=n∑νR˜μ,ν(s)ref(τ,z)∏k=1mgˇνk,mk(rk)ref(τ,z,zk),,
  where $z_{k}=\left(z_{j_{k}+1},\ldots ,z_{j_{k+1}}\right)$. Although it looks identical to the previous equation on $g_{\mu ,\mu }^{(r)\mathrm{ref}}\left(\tau ,z\right)$, it is supplemented by a new normalization condition for n=1:
  (155) $${\overset{ˇ}{g}}_{\mu ,\mu^{\prime }}^{(r)\mathrm{ref}}\left(\tau ,z,z^{\prime }\right)=\delta_{\mu ,\mu^{\prime }}\;\prod_{\alpha =1}^{d_{r}}\theta_{1}\left(\tau ,t_{\alpha }^{\left(r\right)}z^{\prime }\right).$$
  The crucial property of (154) is that its solutions that are regular at z=0 give rise to the functions $g_{\mu ,\mu }^{(r)\mathrm{ref}}\left(\tau ,z\right)$ introduced at the previous step. The relation between the two sets of functions is given by
  (156) $$g_{\mu ,\mu }^{(r)\mathrm{ref}}\left(\tau ,z\right)=\frac{1}{{\left(-2\pi \eta^{3}\left(\tau \right)\right)}^{d_{r}}}\left(\prod_{i=1}^{n}\frac{D_{\frac{1}{2}{\left(t^{\left(r_{i}\right)}\right)}^{2}}^{\left(d_{r_{i}}\right)}\left(z_{i}\right)}{d_{r_{i}}!\prod_{\alpha =1}^{d_{r_{i}}}t_{\alpha }^{\left(r_{i}\right)}}\right){\overset{ˇ}{g}}_{\mu ,\mu }^{(r)\mathrm{ref}}{|}_{z=0},$$
  where the differential operators $D_{m}^{\left(n\right)}$ are defined in (33).
  It is the presence of the additional factors of the Jacobi theta function in (155) that leads to an effective extension of the lattice defining the theta series that capture the coefficients on the r.h.s. of (154). While the original lattice, which can be read off, e.g., from (146), is given by
  (157) $$\Lambda^{\left(r\right)}=\left\{k\in Z^{n}\;:\;\sum_{i=1}^{n}r_{i}k_{i}=0\right\}$$
  with the bilinear form
  (158) $$x\cdot y=\kappa \sum_{i=1}^{n}r_{i}x_{i}y_{i},$$
  its extended version turns out to be

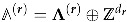
(159)
and carries the bilinear form
  (160) $$x*y=\sum_{i=1}^{n}\left(\kappa r_{i}x_{i}y_{i}-\sum_{\alpha =1}^{d_{r_{i}}}x_{i,\alpha }y_{i,\alpha }\right),$$
  where $x=\{x_{i},x_{i,\alpha }\}$ with i=1,…,n and $\alpha =1,\ldots ,d_{r_{i}}$. Since the signature of (160) is $\left(n-1,d_{r}\right)$, 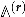 has many null vectors. In the following we will use two sets of vectors belonging to 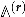 with the second set consisting of null vectors. Both sets are extensions of the vectors $v_{ij}\in \Lambda^{\left(r\right)}$, defined as in (76)
  (161) $${\left(v_{ij}\right)}_{k}=\delta_{ki}r_{j}-\delta_{kj}r_{i},$$
  and given by
  (162) $$\begin{array}{cc} {\left(v_{ij}\right)}_{k}= & \;{\left(v_{ij}\right)}_{k},\;\;\;{\left(v_{ij}\right)}_{k,\alpha }=0,\\ {\left(w_{ij}\right)}_{k}= & \;2^{\epsilon }{\left(v_{ij}\right)}_{k},\;{\left(w_{ij}\right)}_{k,\alpha }={\left(v_{ij}\right)}_{k}, \end{array}$$
  where the factor of $2^{\epsilon }$ compensates the factor of $4^{\epsilon }$ appearing in $d_{r}$ and ensures that $w_{ij}^{2}=0$. We will also use their normalized versions ${\hat{v}}_{ij}=v_{ij}/r_{ij}$ and ${\hat{w}}_{ij}=w_{ij}/r_{ij}$ where $r_{ij}=\gcd\left(r_{i},r_{j}\right)$.
  Note also that the theta series appearing on the r.h.s. of (154) depend on the following vector of refinement parameters
  (163) $$z=\left(\theta^{\left(r\right)}z;-t^{\left(r_{1}\right)}z_{1};\ldots ;-t^{\left(r_{n}\right)}z_{n}\right),\;\theta^{\left(r\right)}=\sum_{i<j}v_{ij}.$$
  For all null vectors, $z*w_{ij}$ is proportional to *z* and independent of z.
3. Then one solves the refined system of anomaly equations on the extended lattice (154) using the null vectors introduced in (162).
4. After that, one reduces the solution to the original lattice using the relation (156).
5. Finally, one evaluates the unrefined limit by means of
  (164) $$g_{\mu ,\mu }^{\left(r\right)}\left(\tau \right)=\lim_{y\to 1}{\left(y-y^{-1}\right)}^{1-n}g_{\mu ,\mu }^{(r)\mathrm{ref}}\left(\tau ,z\right).$$

**Figure 5:**
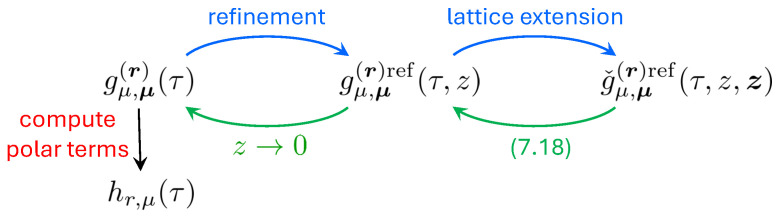
Construction of the anomalous coefficients through the refinement and lattice extension and their relation to the generating functions of BPS indices.

This procedure is schematically presented in Figure 5.

#### 7.4.2. Lattices, Glue Vectors and Discriminant Groups

Before we present the result of the procedure outlined above, let us spell out a few properties of the relevant lattices.

First, let us consider a sublattice generated by the vectors (162). More precisely, we denote by 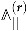 a sublattice of 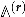 given by the span with integer coefficients of the normalized vectors ${\hat{v}}_{ij},{\hat{w}}_{ij}$, 1≤i<j≤n. It is easy to see that

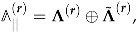
(165)
where $\Lambda^{\left(r\right)}=\;\mathrm{Span}\;\left\{{\hat{v}}_{ij}\right\}$, while ${\tilde{\Lambda }}^{\left(r\right)}=\;\mathrm{Span}\;\left\{{\hat{u}}_{ij}\right\}$ with ${\hat{u}}_{ij}={\hat{w}}_{ij}-2^{\epsilon }{\hat{v}}_{ij}$ is isomorphic to $\Lambda^{\left(r\right)}$ with a quadratic form rescaled by $-4^{\epsilon }$. The orthogonal completion of 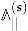 in 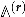, denoted by 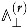, is a direct sum of n+1 lattices

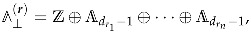
(166)
where $A_{N}$ is the root lattice of the corresponding Lie algebra. The different factors in (166) are generated by the vectors

(167) $$\begin{array}{cc} {\left(e_{0}\right)}_{k}= & \;0,\;{\left(e_{0}\right)}_{k,\beta }=1,\\ {\left(e_{i,\alpha }\right)}_{k}= & \;0,\;{\left(e_{i,\alpha }\right)}_{k,\beta }=\delta_{ik}\left(\delta_{\alpha +1,\beta }-\delta_{\alpha \beta }\right). \end{array}$$

Importantly, the full lattice 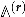 is *not* a direct sum of 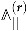 and 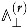, but requires the introduction of glue vectors (see Appendix C). Namely, one has

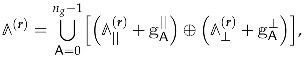
(168)
where the glue vectors are labeled by the set $A=\{a_{0},a_{1},\ldots ,a_{n}\}$ with the indices taking values in the following ranges: $a_{0}=0,\ldots ,r/r_{0}-1$ and $a_{i}=0,\ldots ,d_{r_{i}}-1$. Explicitly, they can be chosen as

(169) $$g_{A}=a_{0}g_{0}+\sum_{i=1}^{n}g_{i,a_{i}},$$

where

(170) $$\begin{array}{cc} {\left(g_{i,a}\right)}_{k}= & \;0,\;{\left(g_{i,a}\right)}_{k,\alpha }=\delta_{ik}\sum_{\beta =1}^{a}\delta_{\alpha \beta },\;g_{0}=\sum_{i=1}^{n}\rho_{i}g_{i,d_{r_{i}}}, \end{array}$$

and $\rho_{i}$ are fixed integers satisfying $\sum_{i=1}^{n}\rho_{i}r_{i}=r_{0}\equiv \gcd\left(r_{1},\ldots ,r_{n}\right)$.

The factorization (168) plays an important role in solving the anomaly equations for the anomalous coefficients because it allows to disentangle the refinement parameters *z* and z: they appear only in the theta series defined by 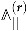 and 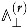, respectively. This in turn leads to two simplifications. First, the theta series based on 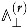 decouples from the problem of ensuring the existence of zero of order n−1 at z=0 needed for the unrefined limit. Second, due to the additional factorization property (166), the refinement parameter $z_{i}$ appears only in the theta series defined by the corresponding $A_{N}$ lattice. As a result, applying (156) to recover $g_{\mu ,\mu }^{(r)\mathrm{ref}}\left(\tau ,z\right)$, each differential operator acts on one theta series only.

Next, we note that there are two ways to represent the elements of the discriminant group $D^{\left(r\right)}={\left(\Lambda^{\left(r\right)}\right)}^{*}/\Lambda^{\left(r\right)}$. On one hand, they can be parametrized by the (n+1)-tuple (μ,μ) taking values in $Z_{\kappa r}⊗\prod_{i=1}^{n}Z_{\kappa r_{i}}$, satisfying the condition $\Delta \mu \in \kappa r_{0}Z$ and subject to the identification $\left(\mu ,\mu \right)≃\left(\mu +r/r_{0},\mu +r/r_{0}\right)$. This is the same tuple that labels the components of the anomalous coefficients. Moreover, the condition on Δμ already appeared in our equations. For example, in (147) it is imposed by the Kronecker delta and, in general, it is a direct consequence of (145). On the other hand, the elements of $D^{\left(r\right)}$ can be represented by a rational *n*-dimensional vector $\hat{\mu }$ with components satisfying $\sum_{i}r_{i}{\hat{\mu }}_{i}=0$. In terms of the previous parametrization, they are given by

(171) $${\hat{\mu }}_{i}=\frac{\mu_{i}}{\kappa r_{i}}-\frac{\mu }{\kappa r}+\frac{\rho_{i}\Delta \mu }{\kappa r_{0}}\;.$$

where $\rho_{i}$ are as in (170). The discriminant group of ${\tilde{\Lambda }}^{\left(r\right)}$ has exactly the same description with κ replaced by $4^{\epsilon }\kappa$. We will denote $\;\hat{\tilde{\;\mu }}$ the vector (171) defined by $\left(\tilde{\mu },\tilde{\mu }\right)$ after this replacement. It is clear that the residue classes of 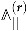 can then be seen either as tuples $\left(\mu ,\mu ;\tilde{\mu },\tilde{\mu }\right)$ or as bi-vectors $\left(\hat{\mu },\;\hat{\tilde{\;\mu }}\right)$. In particular, the residue class given by the projection of the glue vector (169) on 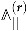 is represented by the tuple with

(172) $$\mu =\mu =0,\;\tilde{\mu }\left(A\right)=4^{\epsilon }\kappa r_{0}a_{0}+\sum_{i=1}^{n}a_{i},\;{\tilde{\mu }}_{i}\left(A\right)=a_{i}.$$

#### 7.4.3. The Result

Let us introduce the following objects:

- A vector valued Jacobi-like form (for n=1, it is taken to be a trivial scalar function $\phi^{\left(r\right)}=1$)
  (173) $$\phi_{\left(\hat{\mu },\;\hat{\tilde{\;\mu }}\right)}^{\left(r\right)}\left(\tau ,z\right)=\frac{\;\mathrm{Sym}\;\{c_{r}\}}{z^{n-1}}\;e^{-\frac{\pi^{2}}{3}\;m_{r}E_{2}\left(\tau \right)z^{2}}\prod_{i=1}^{n}\delta_{{\hat{\mu }}_{i}-2^{\epsilon }\;{\hat{\tilde{\;\mu }}}_{i}}^{\left(1\right)}\;,$$
  where $\left(\hat{\mu },\;\hat{\tilde{\;\mu }}\right)\in D_{\left|\right|}^{\left(r\right)}$ is a residue class of 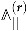 in the bi-vector representation, $m_{r}$ is the index (153), and
  (174) $$c_{r}=\frac{r_{0}}{{\left(2^{\epsilon }\pi i\kappa \right)}^{n-1}r}\prod_{k=1}^{n-1}{\left(\sum_{i=1}^{k}r_{i}\sum_{j=n-k+1}^{n}r_{j}\right)}^{-1}\;;$$
- A function $F^{\left(r\right)}$ playing the role of a kernel of indefinite theta series on 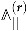


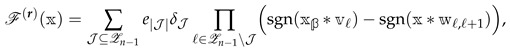
(175)
where $x_{=}x+\sqrt{2\tau_{2}}\;$, $Z_{n}=\left\{1,\ldots ,n\right\}$,

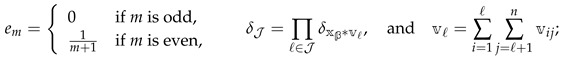
(176)
- A function associated with the lattice 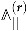  (177) $$\vartheta_{\left(\hat{\mu },\;\hat{\tilde{\;\mu }}\right)}^{\left(r)|\right|}\left(\tau ,z\right)=\sum_{m=1}^{n}\sum_{\sum_{k=1}^{m}n_{k}=n}\sum_{\nu ,\tilde{\nu }}\vartheta_{\mu ,\nu ;\tilde{\mu },\tilde{\nu }}\left(\tau ,z_{\left|\right|}^{\left(s\right)}{;}_{\left|\right|}^{\left(s\right)},F^{\left(s\right)},0\right)\prod_{k=1}^{m}\phi_{\nu_{k},m_{k};{\tilde{\nu }}_{k},{\tilde{m}}_{k}}^{\left(r_{k}\right)}\left(\tau ,z\right),$$
  where s, $r_{k}$ and $m_{k}$ are as in (142) restricted to the one-modulus case, ${\tilde{m}}_{k}$ is defined from $\tilde{\mu }$ similarly to $m_{k}$, $z_{\left|\right|}^{\left(s\right)}=\left(\theta^{\left(s\right)},0\right)$ denotes the projection of z (163) to 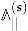, which can be seen as a sublattice of 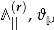 is the generic theta series (42), and on the r.h.s. we used the representation of $D_{\left|\right|}^{\left(r\right)}$ in terms of (2n+2)-tuples;
- The theta series associated with the lattices appearing in the decomposition (166)
  (178) $$\begin{array}{ccc} \vartheta_{\nu }^{\left(d\right)}\left(\tau \right) & = & \sum_{\ell \in Z+\frac{\nu }{d}+\frac{1}{2}}{\left(-1\right)}^{d\ell }\;q^{\frac{d}{2}\;\ell^{2}}, \end{array}$$
  (179) $$\begin{array}{ccc} \Theta_{a}^{\left(N\right)}\left(\tau ,z;t\right) & = & \left(\prod_{\alpha =1}^{N-1}\sum_{\ell_{\alpha }\in Z+\frac{\alpha a}{N}}\right)q^{\sum_{\alpha =1}^{N-1}\left(\ell_{\alpha }^{2}-\ell_{\alpha }\ell_{\alpha +1}\right)}y^{\sum_{\alpha =1}^{N-1}\left(t_{\alpha +1}-t_{\alpha }\right)\ell_{\alpha }}, \end{array}$$
  where we used the convention $\ell_{N}=0$, and the result of the action on $\Theta_{a}^{\left(N\right)}$ of the differential operator in (156)
  (180) $$D\Theta_{a}^{\left(N\right)}\left(\tau ;t\right)=\frac{D_{t^{2}/2}^{\left(N\right)}\Theta_{a}^{\left(N\right)}\left(\tau ,z;t\right){|}_{z_{i}=0}}{N!\left(\prod_{\alpha =1}^{N}t_{\alpha }\right){\left(-2\pi \eta^{3}\left(\tau \right)\right)}^{N}}\;.$$

In terms of these quantities, a solution for the refined anomalous coefficients was found to be

(181) $$g_{\mu ,\mu }^{(r)\mathrm{ref}}\left(\tau ,z\right)=\frac{\delta_{\Delta \mu }^{\left(\kappa r_{0}\right)}}{2^{n-1}}\;\sum_{A}\;\mathrm{Sym}\;\left\{\vartheta_{\left(\hat{\mu },\;\hat{\tilde{\;\mu }}\left(A\right)\right)}^{\left(r)|\right|}\left(\tau ,z\right)\right\}\vartheta_{\tilde{\mu }\left(A\right)}^{\left(d_{r}\right)}\left(\tau \right)\prod_{i=1}^{n}D\Theta_{a_{i}}^{\left(d_{r_{i}}\right)}\left(\tau ;t^{\left(r_{i}\right)}\right),$$

where $\;\hat{\tilde{\;\mu }}\left(A\right)$ denotes the residue class corresponding to the tuple $\left(\tilde{\mu }\left(A\right),\tilde{\mu }\left(A\right)\right)$ defined in (172).

**Figure 6:**
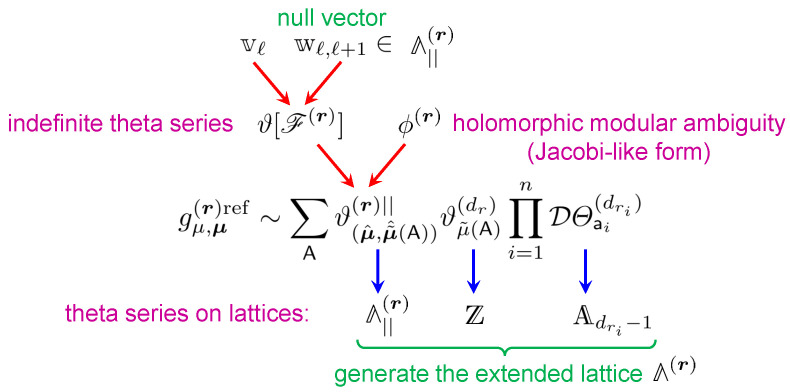
A schematic representation of the solution (181) for the refined anomalous coefficients and its ingredients.

The most non-trivial ingredient of this construction is the function $\vartheta_{\left(\hat{\mu },\;\hat{\tilde{\;\mu }}\right)}^{\left(r)|\right|}$. It recombines indefinite theta series defined by the kernels $F^{\left(r\right)}\left(x\right)$ and the Jacobi-like forms $\phi_{\left(\hat{\mu },\;\hat{\tilde{\;\mu }}\right)}^{\left(r\right)}$ in such a way that it has a zero of order n−1 at z=0. (Although this fact was not proven in all generality, it has been extensively checked both analytically and numerically). As a result, $g_{\mu ,\mu }^{(r)\mathrm{ref}}$ have a well-defined unrefined limit that should be computed using (164) to extract the anomalous coefficients $g_{\mu ,\mu }^{\left(r\right)}$ we are interested in. Unfortunately, this turns out to be the most non-trivial step and so far it has been accomplished analytically only for n=2 and 3. Explicit expressions for $g_{\mu ,\mu_{1},\mu_{2}}^{\left(r_{1},r_{2}\right)}$ and $g_{\mu ,\mu_{1},\mu_{2},\mu_{3}}^{\left(r_{1},r_{2},r_{3}\right)}$ for generic charges as well as their Fourier series for a few small charges and some choice of the vectors $t^{\left(r\right)}$ can be found in [40]. In Figure 6, we showed schematically the main ingredients of the solution (181).

## 8. Applications

In this section, we present three very different applications of the results described in the previous sections. The first is an explicit evaluation of various topological invariants of compact CY threefolds, the second is a solution of Vafa–Witten theory on rational surfaces, and the third is a surprising extension of the above formalism to string compactifications preserving more than eight supercharges, which allows not only to reproduce many known results but also to obtain something new.

### 8.1. DT, PT, GV and Topological Strings

#### 8.1.1. Polar Terms from Wall-Crossing

The results explained in Section 7 reduce the problem of finding the generating series of rank 0 DT invariants to the problem of computing their polar terms. First attempts to do this have been undertaken already quite some time ago for several one-modulus CYs and unit D4-brane charge, in which case the anomaly is absent and the two-step procedure advocated in the beginning of the previous section reduces to the second step. In [7,34], the polar terms were calculated by a direct count of D-branes in the geometry and in [35,36] through a representation via the attractor flow trees [23,100] in terms of bound states of D6 and $\bar{D6}$-branes. However, at this point it is not clear how to generalize these results to other cases by applying the same methods. Therefore, to make progress in the computation of polar terms, one should look for alternative approaches.

An interesting possibility, which often works in the non-compact case, is to find a chamber in the moduli space where the BPS spectrum is simple enough to be computed exactly and then perform wall-crossing to the large volume chamber. Unfortunately, for compact CY threefolds there is no such chamber. Nevertheless, an interesting phenomenon happens if one goes off the physical slice in the space of stability conditions defining the generalized DT invariants, as explained in Section 4.2.

**Figure 7:**
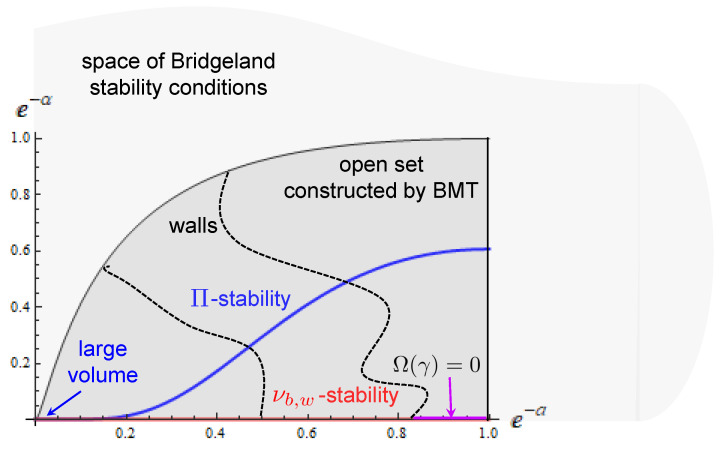
Section of the space of Bridgeland stability conditions by the plane β=0, b=const, drawn in coordinates $\left(e^{-a},e^{-\alpha }\right)$ and the set constructed in [86,131]. The red line is the slice of weak $\nu_{b,w}$-stability conditions with the central charge (182) and the blue line represents the physical slice of Π-stability conditions parametrized by the complexified Kähler moduli of Y. The large volume limit corresponds to the region near the origin where the two slices approach each other.

Let us again restrict to CY threefolds with one Kähler modulus. In this case the space of stability conditions, modulo the action of a symmetry group, can be parametrized by four real variables (a,b,α,β) and an open set of such stability conditions has been rigorously constructed in [86,131]. The subset corresponding to the physical Π-stability is a real-codimension two slice of this open set parametrized by the complexified Kähler modulus z=b+it. Its explicit parametrization is determined by the prepotential F(X) and can be found in ([37], Eq.(2.51)). On the other hand, the boundary of the open set corresponding to α=∞ and β=0 defines another set of (weak) stability conditions playing a crucial role in our story (see Figure 7). It is called $\nu_{b,w}$-stability (here *w* refers the parameter $w=\frac{1}{2}\left(a^{2}+b^{2}\right)$, which simplifies the wall-crossing analysis) and defined by the central charge

(182) $$Z_{b,a}\left(\gamma \right)=-a{\mathrm{ch}}_{2}^{b}+\frac{1}{2}\;a^{3}{\mathrm{ch}}_{0}^{b}+i\;a^{2}{\mathrm{ch}}_{1}^{b}\;,$$

which we wrote in terms of shifted Chern classes that can be compactly written as

(183) $${\mathrm{ch}}_{k}^{b}\left(E\right)=\int_{Y}e^{-b\omega }\omega^{3-k}\mathrm{ch}\left(E\right),$$

where ω is the generator of $H^{2}\left(Y,Z\right)$. The central charge (182) can be obtained from the physical central charge by dropping all quantum corrections to the classical prepotential (51), omitting the contributions proportional to the D0-brane charge and, after substituting (48), to the second Chern class $c_{2}\left(TY\right)$, and finally setting $t=a\sqrt{3}$. Importantly, in the large volume region, the two notions of stability coincide, which means that the DT invariants defined by them are also the same.

The advantage of the $\nu_{b,w}$-stability conditions is twofold. First, all stable objects with respect to the $\nu_{b,w}$-stability were conjectured to satisfy the following *BMT inequality* [86,131]

(184) $${\mathrm{ch}}_{3}^{b}\leq \frac{a^{2}}{6}\;{\mathrm{ch}}_{1}^{b}.$$

It implies that for a given charge there can be a region in the moduli space at small *a* where the corresponding DT invariant must vanish. This suggests that the same DT invariant evaluated in the large volume chamber can be calculated by starting from the chamber where it vanishes and then performing wall-crossing towards the large volume. The second advantage of the $\nu_{b,w}$-stability is its independence of the D0-brane charge, which significantly simplifies the wall-crossing analysis and makes the proposed strategy doable. This idea has been pursued in a recent series of works [132,133,134,135] and led to explicit formulas [136] relating rank 0 DT invariants, counting D4-D2-D0 bound states, to rank ±1 invariants, counting D6-D2-D0 bound states with one unit of D6 or $\bar{D6}$-brane charge. These formulas have been further improved in [37,41] and made possible to formulate a systematic procedure for computing the polar and other terms of the generating series $h_{p,\mu }$.

There are actually two types of formulas that can be used to compute rank 0 DT invariants. The first one is in the spirit of [35,36] and expresses rank 0 DT invariants as a sum of products of rank 1 and rank −1 DT invariants, given by the standard DT and PT invariants. Physically, this precisely corresponds to a sum over D6-$\bar{D6}$ bounds states. The formula reads

(185) $${\bar{\Omega }}_{r,\mu }\left({\hat{q}}_{0}\right)=d_{\mathrm{tors}}^{2}\sum_{r_{i},Q_{i},n_{i}}{\left(-1\right)}^{\gamma_{12}}\;\gamma_{12}\;\mathrm{PT}\left(Q_{1},n_{1}\right)\;\mathrm{DT}\left(Q_{2},n_{2}\right)\;,$$

where DT(Q,n) and PT(Q,n) denote the standard invariants, which depend just on two charges due to the spectral flow invariance, $\gamma_{12}=r\left(Q_{1}+Q_{2}\right)+n_{1}+n_{2}-\chi \left(O_{D_{r}}\right)$ with $\chi (O_{D_{r}})$ defined in (91), and $d_{\mathrm{tors}}$ is the order of the torsion part of the second cohomology group, which in general has the form $H^{2}\left(Y,Z\right)=Z^{b_{2}}⊕Z_{d_{\mathrm{tors}}}$. Up to the torsion factor, each term in the sum can be recognized as a contribution of the primitive wall-crossing Formula (52). The factor $d_{\mathrm{tors}}$ appears because the rank ±1 generalized DT invariants, corresponding to the BPS indices on the r.h.s. of (52), coincide with DT(Q,n) and PT(Q,n) only up to this factor [132]. Its presence has recently been confirmed in [38]. To fully specify the formula, one also needs to provide the range of summation for all six variables in (185). We refer to ([37], §4.1) for these details. Finally, the derivation of the formula implies that it is expected to be valid only in the range

(186) $$0\leq \frac{\chi (D_{r})}{24}-{\hat{q}}_{0}<\frac{\kappa r}{12}\min\left(\frac{r^{2}}{2}-\frac{1}{8}\;,\;r-\frac{1}{2}\right),$$

where $\chi (D_{r})$ is the topological Euler characteristic of the divisor $D_{r}$

(187) $$\chi \left(D_{r}\right)=\kappa r^{3}+c_{2}r.$$

The first inequality in (186) is just the Bogomolov–Gieseker bound (60), while the second is the condition of the existence of the chamber violating the BMT inequality (184), which we can call *empty chamber* due to the absence there of stable objects of the given charge.

Unfortunately, the condition (186) is so restrictive that for r=1 the Formula (185) can only apply, at best, to the most polar term in each component of the modular vector $h_{1,\mu }$. In particular, for μ=0 it is valid only for ${\hat{q}}_{0}=\frac{\chi (D_{r})}{24}$. In this case only the term with $Q_{i}=n_{i}=0$ contributes to (185), which gives

(188) $${\bar{\Omega }}_{r,0}\left(\frac{\chi (D_{r})}{24}\right)=d_{\mathrm{tors}}^{2}{\left(-1\right)}^{1+\chi (O_{D_{r}})}\chi \left(O_{D_{r}}\right)\;.$$

In practice, however, it was observed that the Formula (185) predicts the correct polar terms in many examples with r=1, provided one restricts the sum only to $Q_{1}=n_{1}=0$. Using PT(0,0)=1, one arrives at the naive ansatz for polar coefficients suggested in ([39], Eq.(5.20)):

(189) $${\bar{\Omega }}_{r,\mu }\left({\hat{q}}_{0}\right)=d_{\mathrm{tors}}^{2}{\left(-1\right)}^{r\mu +n+\chi (O_{D_{r}})}\left(r\mu +n-\chi \left(O_{D_{r}}\right)\right)\;\mathrm{DT}\left(\mu ,n\right)\;,$$

where

(190) $$n=\frac{\chi (D_{r})}{24}-\frac{\mu^{2}}{2\kappa r}-\frac{r\mu }{2}-{\hat{q}}_{0}\in Z\;.$$

The fact that it holds in many cases indicates that it should be possible to extend the range of validity of the Formula (185). On the other hand, it remains still unclear what determines when it works and when it fails.

The second formula, derived assuming the triviality of the torsion, i.e., $d_{\mathrm{tors}}=1$, instead expresses a PT invariant PT(Q,m) in terms of invariants $\mathrm{PT}(Q^{\prime },m^{\prime })$ with $Q^{\prime }<Q$ and rank 0 DT invariants $\Omega_{r,\mu }\left({\hat{q}}_{0}\right)$. In its simplest version it reads

(191) $$\mathrm{PT}\left(Q,m\right)=\sum_{Q^{\prime },m^{\prime }}{\left(-1\right)}^{\gamma_{12}}\gamma_{12}\;\mathrm{PT}\left(Q^{\prime },m^{\prime }\right)\;\Omega_{1,Q-Q^{\prime }}\left({\hat{q}}_{0}^{\prime }\right)\;,$$

where $\gamma_{12}=Q+Q^{\prime }+m-m^{\prime }-\chi \left(O_{D_{1}}\right)$,

(192) $${\hat{q}}_{0}^{\prime }=m^{\prime }-m-\frac{1}{2\kappa }{\left(Q^{\prime }-Q\right)}^{2}-\frac{1}{2}\;\left(Q+Q^{\prime }\right)+\frac{\chi (D)}{24}\;,$$

and we refer to ([37], §4.2) for the exact range of summation. This formula holds provided $f_{1}\left(x\right)<\alpha$ where

(193) $$x=\frac{Q}{\kappa }\;,\;\alpha =-\frac{3m}{2Q}\;,$$

and $f_{1}\left(x\right)$ is a piece-wise linear function given by $\frac{1}{2}x+1$ for x≥3. Similarly to (186), this condition ensures the existence of the empty chamber, but in addition it also guarantees that on the way from this chamber to the large volume region only the walls corresponding to bound states with a single D4-brane are encountered.

An important property of (191) is that the term with $\left(Q^{\prime },m^{\prime }\right)=\left(0,0\right)$ for which PT(0,0)=1 always contributes to the sum. This fact allows to invert the relation and express $\Omega_{1,Q}\left({\hat{q}}_{0}\right)$ with ${\hat{q}}_{0}$ given by (192) through other invariants. In practice, however, this does not allow yet to get the rank 0 DT invariants for the charges of interest. Such charges typically spoil the condition $f_{1}\left(x\right)<\alpha$. Fortunately, one can always use the spectral flow invariance (56) to make D2-brane charge large enough so that the condition becomes satisfied. As a result, *any* rank 0 DT invariant with r=1 can be expressed through PT invariants and other rank 0 DT invariants with smaller charges ([37], Eq.(4.19)). Importantly, however, the smaller the D0-brane charge $q_{0}$ we want to consider, the larger the degree *Q* for which PT invariants have to be calculated.

If one relaxes the condition $f_{1}\left(x\right)<\alpha$ in a way that still ensures the existence of the empty chamber, the Formula (191) gets modified by acquiring terms on the r.h.s. that involve bound states with the D4-brane charge r>1. One can show that *r* is the maximal appearing D4-brane charge if $f_{r}\left(x\right)<\alpha$ where $f_{r}\left(x\right)=f_{r-1}\left(x\right)$ for $x\leq {\left(r+1\right)}^{2}$ and $f_{r}\left(x\right)=\frac{x}{r+1}+\frac{r+1}{2}$ in the range $x>{\left(r+1\right)}^{2}$. The modified version of the formula has been computed so far only for r=2 [41]. For x>4 and $\alpha \leq \frac{3}{8}x+\frac{3}{2}$, it has the same feature as (191) that the r.h.s. contains a term proportional to ${\bar{\Omega }}_{2,Q}\left({\hat{q}}_{0}\right)$. Thus, using again the spectral flow invariance, it can be used to express the rank 0 DT invariants with r=2 through PT invariants and other rank 0 DT invariants with smaller charges.

#### 8.1.2. The Role of Topological Strings

Above, we showed how rank 0 DT invariants can be expressed through rank 1 and −1 invariants. How does this help find them? The point is that, for any CY threefold, there is a systematic procedure to compute rank ±1 invariants (which, however, has its own limitations to be discussed below). This is done using the so-called MNOP relation [43,44,137], which allows to express DT(Q,m) and PT(Q,m) in terms of the Gopakumar–Vafa (GV) invariants ${\mathrm{GV}}_{Q^{\prime }}^{\left(g\right)}$ with $Q^{\prime }\leq Q$ and $g\leq g_{\max}\left(Q\right)$. The latter are integer valued and count embedded curves C of genus *g* and degree *Q*, while from the physical viewpoint they count BPS particles in the 5d theory obtained by compactifying M-theory on Y [138,139]. A crucial fact is that the GV invariants of genus *g* determine the A-model topological string free energy $F^{\left(g\right)}$ of the same genus and hence can be deduced from a solution of the topological string. For genus g=0, the free energy $F^{\left(0\right)}$ can be found by mirror symmetry techniques, while for g≥1 the free energies are obtained by integrating the holomorphic anomaly equations that they satisfy [45] following the procedure known as “direct integration” [46,47].

Thus, one proceeds through the following steps:

1. First, one solves the A-model topological string by the direct integration method and obtains its partition function
  (194) $$\Psi_{\mathrm{top}}\left(z,\lambda \right)=\exp\left(\sum_{g\geq 0}\lambda^{2g-2}F^{\left(g\right)}\left(z\right)\right).$$
2. It is used to extract the GV invariants by applying the formula [138,139]
  (195) $$\log\Psi_{\mathrm{top}}\left(z,\lambda \right)=\sum_{g=0}^{\infty }\sum_{k=1}^{\infty }\sum_{Q=1}^{\infty }\frac{{\mathrm{GV}}_{Q}^{\left(g\right)}}{k}{\left(2\sin\frac{k\lambda }{2}\right)}^{2g-2}e^{2\pi ikQz}\;.$$
3. In principle, the previous step could be skipped because the MNOP formula directly relates the topological string partition function with the generating functions of DT and PT invariants
  (196) $$\begin{array}{cc} Z_{DT}\left(y,q\right):= & \;\sum_{Q=0}^{\infty }\sum_{m=m_{\min}\left(Q\right)}^{\infty }\mathrm{DT}\left(Q,m\right)\;y^{Q}\;q^{m},\\ Z_{PT}\left(y,q\right):= & \;\sum_{Q=0}^{\infty }\sum_{m=m_{\min}\left(Q\right)}^{\infty }\mathrm{PT}\left(Q,m\right)\;y^{Q}\;q^{m}, \end{array}$$
  which are well defined because the D0-brane charge *m* is restricted from below by the Castelnuovo bound [37,140]
  (197) $$m\geq m_{\min}\left(Q\right)=-\left\lfloor \frac{Q^{2}}{2\kappa }+\frac{Q}{2}\right\rfloor .$$
  The formula reads
  (198) $$\Psi_{\mathrm{top}}\left(z,\lambda \right)=M{\left(-e^{i\lambda }\right)}^{\frac{1}{2}\;\chi_{Y}}Z_{PT}\left(e^{2\pi iz/\lambda },e^{i\lambda }\right),$$
  where $M\left(q\right)=\prod_{k>0}{\left(1-q^{k}\right)}^{-k}$ is the Mac–Mahon function and $\chi_{Y}$ is the Euler characteristic of Y. A similar formula for the generating function of DT invariants follows from the DT/PT relation conjectured in [77] and proven in [141,142], which has the following simple form
  (199) $$Z_{DT}\left(y,q\right)=M{\left(-q\right)}^{\chi_{Y}}\;Z_{PT}\left(y,q\right).$$
  In practice, however, one computes the PT and DT invariants always by passing through the GV invariants and evaluating the generating series degree by degree. Then, it is more convenient to use the plethystic form of the MNOP relation [137]
  (200) $$Z_{PT}\left(y,q\right)=\mathrm{PE}\left[\sum_{Q>0}\sum_{g=0}^{g_{\max}\left(Q\right)}{\left(-1\right)}^{g+1}{\mathrm{GV}}_{Q}^{\left(g\right)}{\left(1-x\right)}^{2g-2}x^{\left(1-g\right)}y^{Q}\right]\left(-q,y\right),$$
  where PE denotes the plethystic exponential
  (201) $$\mathrm{PE}\left[f\right]\left(x,y\right)=\exp\left(\sum_{k=1}^{\infty }\frac{1}{k}\;f\left(x^{k},y^{k}\right)\right).$$
  Note that the Castelnuovo bound (197) combined with the MNOP formula implies a similar bound on the genus of GV invariants
  (202) $$g\leq g_{\max}\left(Q\right)=\left\lfloor \frac{Q^{2}}{2\kappa }+\frac{Q}{2}\right\rfloor +1.$$
4. At the final step, one applies the formulas from Section 8.1.1 to compute the rank 0 DT invariants appearing in the generating series $h_{r,\mu }$. Note that, a priori, this approach is *not* restricted to the polar terms and can be applied to compute any rank 0 DT invariant.

Unfortunately, the described procedure has a fundamental limitation. The problem is that, to determine the topological string free energies by the direct integration method, one should supplement the holomorphic anomaly equations with some boundary conditions fixing the holomorphic ambiguity. Currently, the known conditions include the Castelnuovo bound (202), the conifold gap constraints, and the value of GV invariants for $\left(Q,g\right)=(n\kappa ,1+\frac{1}{2}n\left(n+1\right)\kappa )$ with n∈N, which saturate the Castelnuovo bound. However, the number of these conditions grows slower with genus than the number of parameters to be fixed in the holomorphic ambiguity. As a result, without further input, the direct integration method works only up to a certain genus $g_{\mathrm{integ}}$, and hence, the PT and DT invariants can be computed only up to a certain finite degree $Q_{\mathrm{integ}}$. In turn, this imposes limitations on the number of terms in the generating series that can be computed by this method. This is why the computation of polar terms remains a challenging problem for most compact CY threefolds.

#### 8.1.3. Results

There are two classes of compact CY threefolds that have been analyzed so far by the method explained in Section 8.1.2. In both cases the generating functions of rank 0 DT invariants have been expressed through an over-complete basis of vector-valued weakly holomorphic modular forms constructed from unary theta series with quadratic form κr, Dedekind eta function η(τ), Serre derivative *D* acting as $q\partial_{q}-\frac{w}{12}E_{2}$ on modular forms of weight *w*, and Eisenstein series $E_{4}\left(\tau \right)$ and $E_{6}\left(\tau \right)$. The theta series and the Dedekind eta function allow to obtain the required multiplier system (90) and a number of polar terms, while the Serre derivative and the Eisenstein series are needed to get the weight −3/2.

##### Hypergeometric Threefolds

The first set of CYs is given by one-parameter smooth complete intersections in weighted projective space, which are known as the so-called hypergeometric CY threefolds and include the famous quintic manifold. There are 13 of such CYs, but for 2 of them the current knowledge of GV invariants is insufficient to find the polar terms by the above method even for r=1. For the remaining 11 CYs listed in Table 1, for r=1, all polar terms and hence the generating series $h_{1,\mu }$ have been found in [37]. Furthermore, for 2 CYs, $X_{8}$ and $X_{10}$, in [41] the same has been done for r=2 where the generating series become mock modular forms and one should use one of the solutions of the modular anomaly equation presented in Section 7.

It should be emphasized that, for most of the analyzed CYs, together with the polar terms, many non-polar ones have been computed and perfectly matched the coefficients obtained by the Fourier expansion of the modular forms uniquely fixed by the polar terms. This provided a striking test of (mock) modularity as well as of various mathematical conjectures, such as the BMT inequality (184), which underlie the analysis. Actually, even the fact that the resulting generating series (for r=2, after applying the inverse of (59)) produce integer valued invariants is a highly non-trivial check of their correctness.

It is interesting that for most of the polar coefficients, and even for some non-polar ones, the correct value turns out to be given by the naive ansatz (189), which, a priori, has no reason to hold. It fails only for 8 out of the 72 calculated polar terms. This fact suggests that there should be a way to correct the ansatz, which would open a possibility to compute the polar terms for higher D4-brane charges *r* or other CY threefolds because it requires much less data than the approach based on PT invariants and wall-crossing relations of type (191) used in these calculations. However, it remains unclear which bound state contributions could account for the discrepancy between the ansatz and the correct values even in the eight mentioned cases.

##### Quotients

The second set of CYs consists of various quotients and has been studied in [38]. It includes four one-parameter threefolds ($X_{5}/Z_{5}$, $X_{3,3}/Z_{3}$, $\left(\mathrm{Pfaffian}P^{6}\right)/Z_{7}$ and a smooth double cover of determinantal quintic in $P^{4}$ quotient by $Z_{5}$) and five two-parameter models given in the CICY notation [143] by

(203) $$\begin{array}{cc} \begin{array}{c} P^{2}\\ P^{2} \end{array}\left[\begin{array}{c} 3\\ 3 \end{array}\right]/Z_{3}, & \;\begin{array}{c} P^{2}\\ P^{5} \end{array}\left[\begin{array}{cccc} 1 & 1 & 1 & 0\\ 1 & 1 & 1 & 3 \end{array}\right]/Z_{3},\;\begin{array}{c} P^{4}\\ P^{4} \end{array}\left[\begin{array}{ccccc} 1 & 1 & 1 & 1 & 1\\ 1 & 1 & 1 & 1 & 1 \end{array}\right]/Z_{5},\\ & \begin{array}{c} P^{2}\\ P^{2}\\ P^{2} \end{array}\left[\begin{array}{ccc} 1 & 1 & 1\\ 1 & 1 & 1\\ 1 & 1 & 1 \end{array}\right]/Z_{3},\;\begin{array}{c} P^{2}\\ P^{2}\\ P^{5} \end{array}\left[\begin{array}{cccccc} 1 & 1 & 1 & 0 & 0 & 0\\ 0 & 0 & 0 & 1 & 1 & 1\\ 1 & 1 & 1 & 1 & 1 & 1 \end{array}\right]/Z_{3}. \end{array}$$

We refer to ([38], Table 1) for their topological data. Because of the quotients, the intersection numbers and the second Chern classes of these manifolds are sufficiently small so that, for r=1, the generating series of rank 0 DT invariants have only a single polar term, except $X_{3,3}/Z_{3}$, which has two such terms. Therefore, the whole generating series is determined just by one coefficient, which can be found by using (188) and requires the knowledge of GV invariants only at small genera. The case of $X_{3,3}/Z_{3}$ is a bit more complicated, but can be treated using a combination of (188) and (191).

Note that all the quotients are non-simply connected manifolds and have a non-trivial torsion in the second cohomology group. Thus, the results of [38] have allowed for the first time to test the torsion factor in (188).

#### 8.1.4. Implications for Topological Strings

The knowledge of the generating functions $h_{r,\mu }$ gives access to infinitely many rank 0 DT invariants. This data can be used in the r.h.s. of the wall-crossing relations (191) to compute PT invariants that were unknown before. They, in turn, can feed the MNOP relation to get new GV invariants. In other words, if a generating series $h_{r,\mu }$ has been successfully found, one can invert the procedure of Section 8.1.2 and calculate new sets of topological invariants.

**Figure 8:**
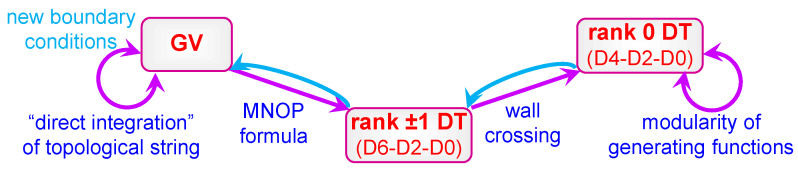
The procedure to obtain the rank 0 DT invariants through the direct integration of topological strings, MNOP formula and wall-crossing relations. The inverse arrows show that, once the generating functions of the rank 0 DT invariants are found, the procedure can be inverted to get new boundary conditions for fixing the holomorphic ambiguity in the direct integration method. One can run this loop multiple times.

Importantly, the GV invariants obtained in this way can serve as new boundary conditions to be used in the direct integration method for fixing the holomorphic ambiguity of the topological string. Thus, one can overcome the limitation of this method explained at the end of Section 8.1.2 and go beyond the genus $g_{\mathrm{integ}}$. We summarize the whole procedure in Figure 8.

Unfortunately, if one knows only a finite number of the generating series $h_{r,\mu }$, one cannot go infinitely far, but only up to a certain new bound $g_{\mathrm{mod}}^{\left(r\right)}$ where *r* is the maximal D4-brane charge for which $h_{r,\mu }$ is known. In Table 1, we provide $g_{\mathrm{integ}}$, $g_{\mathrm{mod}}^{\left(1\right)}$ (and $g_{\mathrm{mod}}^{\left(2\right)}$ for $X_{8}$ and $X_{10}$) as well as the genus up to which the GV invariants have been currently calculated for the 11 hypergeometric CY threefolds analyzed in [37,41]. We also give there the bound $Q_{\mathrm{integ}}$ on the degree of PT invariants and the degree $Q_{\mathrm{avail}}$ that has already been achieved. The full tables of known GV, PT and DT invariants are available at the website [144].

Of course, once one obtains new boundary conditions for the direct integration, one can repeat the procedure shown in Figure 8, and one may hope that this should allow us to compute more polar terms and find more generating functions. In principle, one may think that, if one leaves aside computational problems related to computer speed and memory, it might be possible to overcome all limitations and compute the GV invariants up to arbitrary genus. Unfortunately, this is not the case as the number of polar terms and hence the required genus grow with *r* faster than the number of genera one can gain due to the new boundary conditions [41]. Thus, if one wants to push the idea of the combined use of the holomorphic anomaly of topological string theory and the modular anomaly of the generating series of rank 0 DT invariants, one has to find more powerful wall-crossing relations that would not be so demanding for the GV invariants.

### 8.2. Vafa–Witten Theory

The Vafa–Witten theory is a topological field theory defined on any four-manifold *S*, obtained as one of the three possible topological twists of N=4 SYM theory [54]. We restrict to the case of the gauge group U(r) and *S* a complex Fano or weak Fano surface, which is equipped with a polarization $J\in H^{2}\left(S,R\right)$ such that $J\cdot c_{1}\left(S\right)>0$. Due to this condition and certain vanishing theorems, the functional integral localizes on solutions of hermitian Yang–Mills equations and the partition function is completely determined by the topological invariants, called VW invariants, given by the Euler numbers or, in the refined case, Poincaré polynomials similar to (116) of the moduli spaces of instantons on the surface *S*. (In general, the partition function also receives contributions from the so-called monopole branch; see [145,146,147,148,149,150] for a progress in this direction). Since the refined invariants contain more information and, as we saw in Section 6.3, their description is actually simpler, in the following we will mostly concentrate on the refined case but omit the index “ref” to avoid cluttering.

S-duality of N=4 SYM implies that the partition function of VW theory should be a modular form. Naively, it appears to be a Jacobi form with a theta expansion of the form (28) where the role of $h_{\mu }$ is played by the generating series of VW invariants, which we will denote $h_{r,\mu }^{S}$. This would imply that these generating series are modular forms. However, when $b_{2}^{+}\left(S\right)=1$, the case of our interest, this expectation turns out to be naive because the generating series have a modular anomaly. In fact, they turn out to be examples of mock modular forms of depth r−1 or mock Jacobi forms in the presence of refinement. For instance, already in [54], on the basis of the previous mathematical results [151,152], it was shown that the generating series of (unrefined) SU(2) VW invariants on $P^{2}$ is given by the generating series of the Hurwitz class numbers (Example 7), which was one of the earliest examples of mock modularity in physics. The modularity of the partition function is then supposed to be restored by taking into account non-holomorphic contributions from reducible connections [54]. For the simplest case of SU(2) theory on $P^{2}$, this restoration of modularity was demonstrated in [153], where the required non-holomorphic contributions have been shown to be generated by Q-exact terms due to boundaries of the moduli space, similarly to the holomorphic anomaly in topological string theory [45]. Mathematically, this is nothing but the construction of the completion ${\hat{h}}_{r,\mu }^{S}$ of the mock modular form $h_{r,\mu }^{S}$. Thus, finding the modular completion is an important physical problem in the context of VW theory.

Until a few years ago, only very limited results existed about the modular completions of the generating series of VW invariants, not going beyond r=2 [54,154,155] and r=3 for $P^{2}$ [156]. A breakthrough came from the introduction of the generalized error functions and the results presented in Section 5 and Section 6. The point is that when Y is the non-compact CY given by the total space $\mathrm{Tot}(K_{S})$ of the canonical bundle over a projective surface *S* with $b_{1}\left(S\right)=0$ and $b_{2}^{+}\left(S\right)=1$, as in Example 9, the BPS indices of *r* D4-branes supported on *S* are expected to be equal to the VW invariants of *S* for gauge group U(r) [101,119,120,157], both at the unrefined and refined levels. Physically, this expectation follows from the fact that the topologically twisted N=4U(r) SYM describes the world-volume dynamics of *r* M5-branes wrapped on *S* and dimensionally reduced along $S^{1}$ times the Euclidean time circle. The large volume attractor chamber of Y then corresponds to the so-called canonical chamber in the moduli space parametrized by *J*. It is defined as the chamber containing the point $J=c_{1}\left(S\right)$. This is important to take into account since the VW invariants for $b_{2}^{+}\left(S\right)=1$ and $b_{2}\left(S\right)>1$ depend on the choice of polarization. Therefore, one can identify the generating series of (refined) VW invariants evaluated in the canonical chamber with the generating series of (refined) D4-D2-D0 BPS indices or, more precisely, with their redefined versions ${\tilde{h}}_{r,\mu }$ (143) (or ${\tilde{h}}_{r,\mu }^{\mathrm{ref}}$ in the refined case). As a result, the expressions for the completions (72) and (119) can be directly translated to the context of VW theory. In [28], it was checked that this does reproduce the known completions in the case of $S=P^{2}$.

**Remark** **4.** *One can wonder why the redefined version of the generating functions introduced in the one-modulus case can be applied here. The reason is that the only thing that is important for its definition is that the allowed D4-brane charges are all collinear, whereas the lattice of D2-brane charges can have any dimension. This does hold in our case, where all D4-charges are multiplies of $p_{0}^{a}$ (113) corresponding to the divisor S. To account for multiple dimensions of the electric charge lattice in (143), one should replace μ by $\mu_{a}p_{0}^{a}$ in the sign factor and multiply the shift of μ in the index of the generating function by $p_{0}$.*

However, one can do better and use the modular anomaly equations to actually find the generating series of VW invariants, similarly to how this problem was addressed in the compact case in Section 7 and Section 8.1. Before presenting the results in this direction, let us briefly review what was known before. For r=1, the generating function has been known for any *S* for a long time [158], and when $b_{1}\left(S\right)=0$, it is given in terms of the Jacobi theta and Dedekind eta functions

(204) $$h_{1,0}^{S}\left(\tau ,z\right)=\frac{i}{\theta_{1}\left(\tau ,2z\right)\;\eta {\left(\tau \right)}^{b_{2}\left(S\right)-1}}.$$

For r>1, many explicit expressions already existed in the literature [106,152,155,159,160,161]. Furthermore, for $P^{2}$ a closed formula has been found in [162] in terms of generalized Appell–Lerch sums and, in principle, for any other Fano surface, the generating functions $h_{r,\mu }^{S}$ could be determined by applying a sequence of blow-ups and wall-crossing transitions to this result (see ([114], Figure 1) for a scheme of the blow-up relations between various Fano surfaces). However, this procedure is complicated by the fact that one should pass through the so-called stack invariants, which are polynomial combinations of rational VW invariants having simpler transformation properties under wall-crossing. Another general method to compute the VW invariants based on a relation to quivers and the tree index introduced in [85] was proposed in [114], where many generating functions were given explicitly. However, an explicit formula for the generating functions of arbitrary rank *r* for *S* other than $P^{2}$ remained unknown.

#### 8.2.1. Hirzebruch and del Pezzo

This problem has been addressed in [29] for the Hirzebruch surfaces $F_{m}$ with 0≤m≤2 and the del Pezzo surfaces $B_{m}$ with 1≤m≤8 by solving the modular anomaly equations for the generating functions of refined VW invariants following from (119). The solution is very similar to the one described in Section 7.4 but significantly simpler due to several reasons:

- Since we work with refined invariants from the very beginning, there is no need to introduce the refinement artificially and take the unrefined limit in the end. Furthermore, the generating functions must be mock Jacobi forms and not just Jacobi-like as in Section 7.4. This is because here we are computing the generating functions of Poincaré polynomials depending on the refinement parameter *z* only through $y=e^{2\pi iz}$, whereas there the refinement was just a trick to compute some auxiliary functions.
- There is no need to do a lattice extension because $\Lambda_{S}=H^{2}\left(S,Z\right)$ is a unimodular lattice of signature $\left(1,b_{2}\left(S\right)-1\right)$ and for the Hirzebruch and del Pezzo surfaces $b_{2}\left(S\right)>1$. In all relevant cases, $\Lambda_{S}$ has several null vectors (at least two), but only one of them appears in the construction of indefinite theta series.
- Finally, although one can solve (119), as any anomaly equation, only up to a holomorphic modular ambiguity, the ambiguity is severely constrained by the requirements to be a Jacobi form with given modular properties and to ensure the existence of the unrefined limit. As a result, after comparing with known results in the literature, it is possible to suggest a universal ansatz for this ambiguity so that there is no need to fix it through the computation of any polar terms.

To present the resulting generating functions, let us introduce several ingredients:

- From Example 9, we know that the relevant magnetic charges are all collinear, $p^{a}=rp_{0}^{a}$, with $p_{0}^{a}$ determined by the first Chern class of the surface (113). The relevant lattice of electric charges is $\Lambda_{r}=r\Lambda_{S}$ with the quadratic form $rC_{\alpha \beta }$ where $C_{\alpha \beta }=D_{\alpha }\cap D_{\beta }$ is the intersection matrix on *S* (see (115)), specified for Hirzebruch and del Pezzo surfaces in Appendix E. This motivates the introduction of the reduced charge vector $\hat{\gamma }=\left(r,q_{\alpha }\right)$ where the electric charge can be decomposed as (cf. (144))
  (205) $$q_{\alpha }=r\;C_{\alpha \beta }\epsilon^{\beta }+\mu_{\alpha }-\frac{r}{2}\;C_{\alpha \beta }c_{1}^{\beta },\;\epsilon^{\alpha }\in Z.$$
  One can also show that the quadratic form (74) takes the form
  (206) $$Q_{n}\left(\left\{{\hat{\gamma }}_{i}\right\}\right)=\frac{1}{r}\;q^{2}-\sum_{i=1}^{n}\frac{1}{r_{i}}q_{i}^{2}=-\sum_{i<j}\frac{{\left(r_{i}q_{j}-r_{j}q_{i}\right)}^{2}}{rr_{i}r_{j}}\;,$$
  where $q^{2}=C^{\alpha \beta }q_{\alpha }q_{\beta }$ and $C^{\alpha \beta }$ is the inverse of $C_{\alpha \beta }$, and for the charges satisfying $\sum_{i}q_{i}=q$ with *q* fixed, its signature is $\left(\left(n-1\right)\left(b_{2}\left(S\right)-1\right),n-1\right)$.
- We define the anti-symmetrized Dirac product of charges depending on a vector $v\in \Lambda_{S}$:
  (207) $$\gamma_{ij}\left(v\right)=v^{\alpha }\left(r_{i}q_{j,\alpha }-r_{j}q_{i,\alpha }\right).$$
  Note that $\gamma_{ij}\left(c_{1}\right)$ coincides with the usual Dirac product of the reduced charge vectors $\left(r_{i}p_{0}^{a},q_{i,a}\right)$ relevant for the non-compact CY underlying this construction.
- For each surface *S*, we pick a specific null vector $v_{0}\in \Lambda_{S}$. For $F_{m}$ and $B_{m}$, in the basis described in Appendix E, it is given by
  (208) $$v_{0}\left(F_{m}\right)=\left[f\right],\;v_{0}\left(B_{m}\right)=D_{1}-D_{2}.$$
- We define the theta series
  (209) Θℓ(r)(τ,z)=∑∑i=1rki=0ki∈Z+ℓ/rq−∑i<jkikjy∑i<j(kj−ki),
  which transforms as a vector valued Jacobi form of weight $\frac{1}{2}\left(r-1\right)$ and index $\frac{1}{6}\left(r^{3}-r\right)$. One can show that it is a specification of the $A_{r-1}$ theta series $\Theta_{\ell }^{\left(r\right)}\left(\tau ,z;t\right)$ (179) for $t_{\alpha }=r+1-2\alpha$. Combined with a power of the Dedekind eta function, it produces the so-called blow-up functions
  (210) $$B_{r,\ell }\left(\tau ,z\right)=\frac{\Theta_{\ell }^{\left(r\right)}\left(\tau ,z\right)}{\eta {\left(\tau \right)}^{r}}\;,$$
  which relate the generating functions of stack invariants on manifolds related by the blow-up of an exceptional divisor [163,164,165]. In turn, the generating functions of stack invariants evaluated at $J=v_{0}$ (208) are given by [161,166]
  (211) $$H_{r,\mu }^{S}=\delta_{v_{0}\cdot \mu }^{\left(r\right)}\;H_{r}\prod_{\alpha =3}^{b_{2}\left(S\right)}B_{r,\mu^{\alpha }},\;H_{r}=\frac{i{\left(-1\right)}^{r-1}\eta {\left(\tau \right)}^{2r-3}}{\theta_{1}\left(\tau ,2rz\right)\;\prod_{m=1}^{r-1}\theta_{1}{\left(\tau ,2mz\right)}^{2}}\;,$$
  where in the last factor, which is relevant only for del Pezzo surfaces, $\mu^{\alpha }$ are the components of the residue class μ, i.e., $\mu =\mu^{\alpha }D_{\alpha }$, in the basis defined in Appendix E. The functions $H_{r,\mu }^{S}$ are vector-valued Jacobi forms of weight $-\frac{1}{2}b_{2}\left(S\right)$ and index $-(\frac{1}{6}\left(r^{2}-1\right)c_{1}^{2}+2)r$. Note that these are the same weight and index that $h_{r,\mu }^{S}$ are expected to have ([28], Eq.(4.16)) and they agree with the values given in (131) provided one takes $\lambda_{a}p^{a}=r$, which can be achieved, for example, by choosing $\lambda_{a}$ to be the normalized null eigenvector of the quadratic form corresponding to the divisor $D_{e}$ in the notations of Example 9.

Let us now combine all the above ingredients into the functions very similar to (177):

(212) $$\Theta_{r,\mu }^{S}\left(\tau ,z;\left\{F_{n}\right\}\right)=\sum_{n=1}^{r}\frac{1}{2^{n-1}}\sum_{\sum_{i=1}^{n}{\hat{\gamma }}_{i}=\hat{\gamma }}F_{n}\left(\left\{{\hat{\gamma }}_{i}\right\}\right)\;q^{\frac{1}{2}Q_{n}\left(\left\{{\hat{\gamma }}_{i}\right\}\right)}\;y^{\sum_{i<j}\gamma_{ij}\left(c_{1}\left(S\right)\right)}\prod_{i=1}^{n}H_{r_{i},\mu_{i}}^{S}\left(\tau ,z\right),$$

where the sum goes over all decompositions of the reduced charge $\hat{\gamma }=\left(r,\mu -\frac{r}{2}c_{1}\right)$, i.e., with the spectral flow parameter set to zero, the charges $q_{i}$ are quantized as in (205) with *r* replaced by $r_{i}$, and $F_{n}$ is a set of functions playing the role of kernels of indefinite theta series on $\left({⊕}_{i=1}^{n}\Lambda_{r_{i}}\right)/\Lambda_{r}$. In terms of the functions (212), the generating functions of the refined VW invariants evaluated in the canonical chamber can be written simply as

(213) $$h_{r,\mu }^{S}=\Theta_{r,\mu }^{S}\left(\tau ,z;\{F_{n}\left(c_{1}\left(S\right)\right)\}\right).$$

The kernels $F_{n}$ defining these functions depend on a lattice vector and are given by

(214) $$F_{n}\left(\left\{{\hat{\gamma }}_{i}\right\};v\right)=\sum_{J\subseteq Z_{n-1}}e_{\left|J\right|}\;\delta_{J}\left(v\right)\prod_{k\in Z_{n-1}\setminus J}\left(\mathrm{sgn}\left(\Gamma_{k}\left(v\right)\right)-\mathrm{sgn}\left(B_{k}\right)\right),$$

where most of the notations are the same as in (175) except (cf. (130))

(215) $$\begin{array}{cc} \delta_{J}\left(v\right)= & \;\prod_{k\in J}\delta_{\Gamma_{k}\left(v\right)},\;\Gamma_{k}\left(v\right)=\sum_{i=1}^{k}\sum_{j=k+1}^{n}\gamma_{ij}\left(v\right),\\ B_{k}= & \;\gamma_{k,k+1}\left(v_{0}\right)+\beta r_{k}r_{k+1}\left(r_{k}+r_{k+1}\right)\;v_{0}\cdot c_{1}\left(S\right), \end{array}$$

and, as usual, β encodes the imaginary part of the refinement parameter through z=α−τβ. Note that the kernels $F_{n}$ have exactly the same structure as the kernels (175). It should also be clear that the Jacobi forms $H_{r,\mu }^{S}$ play the same role as the Jacobi-like forms $\phi_{\left(\hat{\mu },\;\hat{\tilde{\;\mu }}\right)}^{\left(r\right)}$ in (177). In particular, they ensure the existence of the unrefined limit, canceling all poles of the indefinite theta series, except the first-order pole inherent to the generating functions of refined invariants. They can be seen as holomorphic modular ambiguities of the solution of the modular anomaly equations, which are fixed by consistency and matching the known results.

Furthermore, in [30], it was shown that the result (213) has a very simple generalization to an arbitrary chamber provided it lies in the projection of the Kähler cone on the two-dimensional plane in the moduli space spanned by the first Chern class and the null vector (208), i.e., $J\in \mathrm{Span}{\left(c_{1}\left(S\right),v_{0}\left(S\right)\right)}^{+}$. In this case, it is enough to replace the first Chern class in the argument of $F_{n}$ by polarization *J*. Thus, if we denote $h_{r,\mu ,J}^{S}$ to be the generating functions of the refined VW invariants evaluated at *J*, one obtains

(216) $$h_{r,\mu ,J}^{S}=\Theta_{r,\mu }^{S}\left(\tau ,z;\{F_{n}\left(J\right)\}\right).$$

Once the generating functions are explicitly known and expressed through indefinite theta series, one can immediately find explicit expressions for their modular completions. Applying the recipe (41), one obtains [30]

(217) $${\hat{h}}_{r,\mu ,J}^{\;S}\left(\tau ,z\right)=\Theta_{r,\mu }^{S}\left(\tau ,z;\{{\hat{F}}_{n}\left(J\right)\}\right),$$

where the kernels are expressed through the generalized error functions $\Phi_{n}^{E}$ (A2) as

(218) $${\hat{F}}_{n}\left(\left\{{\hat{\gamma }}_{i}\right\};J\right)=\sum_{J\subseteq Z_{n-1}}\Phi_{\left|J\right|}^{E}\left({\left\{v_{\ell }\left(J\right)\right\}}_{\ell \in J};\sqrt{2\tau_{2}}\;\left(q+\beta \theta \right)\right)\prod_{k\in Z_{n-1}\setminus J}\left(-\mathrm{sgn}(B_{k})\right).$$

Here the vectors q, θ and $v_{\ell }\left(J\right)$ are exactly the same as appears in (121) after specialization to the case under consideration, except that in the definition of $v_{\ell }$, one should replace the magnetic charge by the vector *J*. Explicit expressions for these vectors can be either read off from (215) or found in ([30], §D).

Note that one of the crucial ingredients of the presented construction is the choice of the null vector $v_{0}\left(S\right)$ (208). With mild modifications, a similar construction can be carried out for other choices as well. For some of them, the resulting generating functions turn out to be the same as (216). This coincidence has been interpreted in [29] as a manifestation of the fiber-base duality [118,167], with the prototypical example given by $F_{0}=P^{1}\times P^{1}$ where the second null vector is $v_{0}^{\prime }\left(F_{0}\right)=\left[s\right]$, which is geometrically indistinguishable from $v_{0}\left(F_{0}\right)=\left[f\right]$. In more complicated examples involving del Pezzo surfaces, it can be traced back to the Weyl reflection symmetry of the lattice $\Lambda_{B_{m}}$ [168]. In all cases, it can be used to generate non-trivial identities between the generalized Appell–Lerch functions, which provide an alternative way to express the generating functions.

However, in most cases, the other choices of the null vector lead to different functions, but satisfying the same modular anomaly equations. Of course, the Fourier coefficients of the new functions are *not* VW invariants, but the fact that they possess the same modular properties begs for an explanation. In particular, one can ask whether they can be interpreted as a new kind of topological invariants of *S*. One should keep in mind, however, that the coefficients of the generating series are rational numbers, and whereas in the case of VW invariants they *must* produce integer numbers by inverting the Formula (59), this is not the case for the new numbers, which might be a serious obstacle in attempt to find their mathematical interpretation.

#### 8.2.2. $P^{2}$

It is natural to ask whether the construction presented above can also be applied to the simplest surface $S=P^{2}$. In this case $b_{2}\left(S\right)=1$ and therefore we encounter exactly the same problem that we had in Section 7.4: a one-dimensional lattice does not have null vectors. But we also know a solution to this problem—lattice extension. In the case of VW theory, such a lattice extension can be done in a way that has a geometric interpretation as the blow-up of a point into an exceptional divisor. It increases $b_{2}\left(S\right)$ by 1, thereby increasing the dimension of the lattice $\Lambda_{S}$. In particular, the blow-up of $P^{2}$ gives $F_{1}=B_{1}$. After the blow-up, the lattice has an indefinite signature and has null vectors so that it is amenable to the above construction.

This procedure has been carried out in [30], where it was shown that it leads to a version of the blow-up formula [163,164,165], which relates the generating functions on two surfaces, *S* and $\overset{ˇ}{S}$, where the second is the blow-up of the first. Denoting the exceptional divisor appearing due to the blow-up by $D_{e}$ and the obvious lattice embedding by $\iota :\Lambda_{S}↪\Lambda_{\overset{ˇ}{S}}$, one finds the following relation

(219) $$h_{r,\mu ,J}^{S}\left(\tau ,z\right)=\frac{h_{r,\iota \left(\mu \right)+\ell D_{e},\iota \left(J\right)}^{\overset{ˇ}{S}}\left(\tau ,z\right)}{B_{r,\ell }\left(\tau ,z\right)}\;,$$

where $B_{r,\ell }$ are the blow-up functions (210). An analogous relation can be written for the modular completions as well. Applying the Formula (219) to the case $S=P^{2}$, one obtains

(220) $$h_{r,\mu }^{P^{2}}\left(\tau ,z\right)=\frac{\Theta_{r,\mu D_{1}+\ell D_{2}}^{F_{1}}\left(\tau ,z;\{F_{n}\left(D_{1}\right)\}\right)}{B_{r,\ell }\left(\tau ,z\right)}\;,$$

where on the r.h.s. we used the basis (A29).

Note that the index *ℓ* on the r.h.s. of the above equations is arbitrary. Therefore, the functions $h_{r,\overset{ˇ}{\mu },\iota \left(J\right)}^{\overset{ˇ}{S}}$ must satisfy integrability conditions ensuring the independence of the ratios (219) on this index, which can be viewed as consistency conditions of the construction. (In Section 7.4, such integrability conditions were avoided by ensuring that the discriminant group of the extended lattice 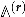 is equal to the discriminant group of the original lattice $\Lambda^{\left(r\right)}$ for any set of charges r. This is why 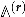 had to be much bigger than $\Lambda^{\left(r\right)}$. In our case, this holds only for r=1, whereas for generic *r* the discriminant group after the blow-up gets an additional factor $Z_{r}$). For $S=P^{2}$, they are known to follow at r=2 from the periodicity property of the classical Appell function and at r=3 from its generalization proven in [169]. For higher ranks, they remain unexplored.

In fact, it is possible to rewrite (219) in a “covariant” form that makes the modular properties of the resulting generating functions obvious. We will write it for their modular completions, but a similar formula holds for the generating functions themselves. It reads

(221) $${\hat{h}}_{r,\mu ,J}^{\;S}\left(\tau ,z\right)=\frac{\eta {\left(\tau \right)}^{r}}{\prod_{j=1}^{r}\theta_{1}\left(\tau ,\left(2j-1\right)z\right)}\sum_{\ell =0}^{r-1}\theta_{r,\ell }\left(\tau ,rz\right)\;{\hat{h}}_{r,\iota \left(\mu \right)+\ell D_{e},\iota \left(J\right)}^{\;\overset{ˇ}{S}}\left(\tau ,z\right),$$

where

(222) $$\theta_{r,\ell }\left(\tau ,z\right)=\sum_{k\in rZ+\ell +\frac{1}{2}r}q^{\frac{1}{2r}\;k^{2}}\;{\left(-y\right)}^{k}.$$

Its specification for $S=P^{2}$ can be obtained as in (220).

We finish this discussion by noticing that the use of the blow-up relations to obtain the generating functions of VW invariants is certainly not new. This is precisely how a representation in terms of the generalized Appell–Lerch sums was derived in [162]. What is new here is that these relations are applied directly to the generating functions. As was mentioned below (204), usually, one should pass through stack invariants instead. The main reason for this detour is that the blow-up relations have to be applied on walls of marginal stability, where the VW invariants, in contrast to the stack invariants, are not defined. For example, in (220), the polarization $J=D_{1}$ for $F_{1}$ is a wall of marginal stability for (some of) the VW invariants with $\overset{ˇ}{\mu }={\overset{ˇ}{\mu }}^{2}D_{2}$. A miraculous property of the representations (216) and (217) is that they continue to be well-defined on the walls! This happens because the modular completions are actually smooth across the walls and provide an unambiguous definition of the holomorphic generating functions everywhere in the moduli space, including the walls, which is realized by a prescription defining the kernels (214) even when some of the Dirac products $\Gamma_{k}\left(v\right)$ vanish. Of course, this does not mean that the VW invariants are defined on the walls. For example, one can check that the rational invariants extracted from $h_{3,0,D_{1}}^{F_{1}}$ do *not* lead to *integer* invariants after application of the inverse of the Formula (59). Nevertheless, they correctly reproduce the VW invariants on $P^{2}$ via (220) [30]. Thus, the representation in terms of indefinite theta series obtained by solving the modular anomaly equations provides an unexpected new insight into the blow-up relations.

### 8.3. Higher Supersymmetry

Up to this point, our analysis was restricted to $\frac{1}{2}$-BPS states in theories with eight supercharges like N=2 supergravity in four dimensions. However, one may ask whether some of the presented results can be generalized to string compactifications with more preserved supercharges. Remarkably, this is indeed possible, and one can write a generalization of the anomaly equations considered above that captures the modular behavior of various BPS indices [33]. Of course, in most cases, this behavior is already well-known, but it is nice to see that there is a single universal framework that describes the modularity of BPS states in all string compactifications.

#### 8.3.1. Helicity Supertraces

The starting point of the construction is the helicity generating function [170]

(223) $$B\left(R,y\right)={Tr}_{R}{\left(-y\right)}^{2J_{3}},$$

where R is a representation of the supersymmetry algebra and $y=e^{2\pi iz}$ is a formal expansion parameter similar to refinement. The coefficients of the Taylor expansion in *z* of B(R,y) are identified with the so-called *helicity supertraces*, which count with sign the short and intermediate multiplets in supersymmetric theories [170]. More precisely, they are given by

(224) $$B_{2K}\left(R\right)={\left(\frac{1}{2}\;y\partial_{y}\right)}^{2K}{B\left(R,y\right)|}_{y=1}={Tr}_{R}\left[{\left(-1\right)}^{2J_{3}}J_{3}^{2K}\right].$$

The insertion of each power of $J_{3}$ in the trace soaks up 2 fermionic zero modes. Since each broken supercharge generates a fermionic zero mode and all of them should be soaked up to get a non-vanishing result, the first helicity supertrace to which a multiplet of $\frac{1}{r}$-BPS states in a 4d theory with N extended supersymmetry can contribute non-trivially is $B_{2K}$ with

(225) $$K=N\left(1-\frac{1}{r}\right).$$

To extract the index $\Omega^{\left(N|r\right)}\left(\gamma \right)$ counting such BPS states of charge γ from $B_{2K}\left(R\right)$, one should substitute $R=H_{\gamma ,j}^{N}$, the Hilbert space of states of charge γ and spin *j*, and factor out the center of mass contribution described by the supersymmetry multiplet $R_{j,2K}$ constructed by acting on a spin *j* ground state with 2K oscillators, i.e.,

(226) $$\Omega^{\left(N|r\right)}\left(\gamma \right)=\frac{B_{2K}\left(H_{\gamma ,j}^{N}\right)}{B_{2K}\left(R_{j,2K}\right)}\;,$$

where *K* is determined by N and *r* through (225).

A crucial observation is that, on one hand, a similar factorization applied to the full helicity generating function in the N=2 case gives rise to the refined BPS indices discussed in Section 6.3, while on the other hand, the ratio defining them has a perfect sense for arbitrary N. In other words, we introduce the refined BPS index in a theory with any number of supersymmetries by the ratio

(227) $$\Omega \left(\gamma ,y\right)=\frac{B(H_{\gamma ,j},y)}{B(R_{j,2},y)}\;.$$

The virtue of such an index is that it encodes all BPS indices $\Omega^{\left(N|r\right)}\left(\gamma \right)$. Indeed, taking into account that $B\left(R_{j,2K},y\right)=O\left(z^{2K}\right)$, one can show that [33]

(228) $$\Omega^{\left(N|r\right)}\left(\gamma \right)=\frac{{\left(-1\right)}^{K-1}}{(2K-2)!}\;{\left(y\partial_{y}\right)}^{2K-2}\Omega \left(\gamma ,y\right){|}_{y=1}.$$

#### 8.3.2. Conjecture

Once one has a universal definition of the refined BPS index which works for any number of supersymmetries, it is natural to use it to define the refined generating functions as in (118) and expect that these functions have modular properties described by anomaly equations similar to (119). However, to make these ideas precise, first, one needs to understand how to incorporate several new features absent in the N=2 case.

Let us recall that compactifications with N=4 and N=8 supersymmetries can be obtained by taking $Y=K3\times T^{2}$ and $Y=T^{6}$, respectively. An important difference of these manifolds compared to CY threefolds with SU(3) holonomy is that $b_{1}\left(Y\right)>0$, which leads to additional scalar and gauge fields in the effective action. In particular, the electromagnetic charge vector can now be represented as $\gamma =(p^{0},p^{A},q_{A},q_{0})$ where *A* runs over $b_{2}+2b_{1}$ values. Hence, the relevant charge lattice is now $\left(b_{2}+2b_{1}\right)$-dimensional. But what is the associated quadratic form? It turns out that it can be read off from the prepotential governing the couplings of vector multiplets in the effective action at the two-derivative level. It has a cubic form

(229) $$F^{\mathrm{cl}}\left(X\right)=-\frac{\kappa_{ABC}X^{A}X^{B}X^{C}}{6X^{0}}\;,$$

with the tensor $\kappa_{ABC}$ extending the tensor of intersection numbers [171]. Then the natural quadratic form is defined, as usual, as $\kappa_{AB}=\kappa_{ABC}p^{C}$.

It turns out that in most cases with N>2 the quadratic form $\kappa_{AB}$ is degenerate with some number of null eigenvalues. But how to deal with such cases has already been explained in Section 6.1: the lattice of electric charges should be restricted to the sublattice orthogonal to the null eigenvectors, while the weight and index of the refined generating functions change to (131).

Finally, a genuinely new feature of compactifications with higher supersymmetry is that the quadratic form $\kappa_{AB}$ can have signature $\left(n_{+},n_{-}\right)$ with both $n_{\pm }>1$. This fact may drastically affect the modular anomaly because a naive extension of the existing construction would lead to divergent theta series. In the following, we simply assume that there is a modification of functions $E_{n}^{\left(\mathrm{ref}\right)}$ that takes care about this problem. This will be sufficient for our purposes since no explicit expressions in such problematic cases will be required.

Thus, given the refined BPS indices (227), we define the generating functions $h_{p,\mu }^{\mathrm{ref}}\left(\tau ,z\right)$ (118) in terms of their rational counterparts (117). The difference with the previous definitions is that now it is the quadratic form $\kappa_{AB}$ that defines the invariant charge ${\hat{q}}_{0}=q_{0}-\frac{1}{2}\;\kappa^{AB}q_{A}q_{B}$. Then we claim

**Conjecture** **2.** *The refined generating functions $h_{p,\mu }^{\mathrm{ref}}\left(\tau ,z\right)$ are higher depth mock Jacobi forms of weight and index (131), where $\Lambda_{p}$ is the lattice with the quadratic form $\kappa_{AB}$, and with modular completions satisfying (119) and the refined version of (88) where the functions $R_{\mu ,\mu }^{(r)\mathrm{ref}}$ and $J_{n}^{\left(\mathrm{ref}\right)}$ are constructed using the same lattice and have a zero of order n−1 at z=0.*

In fact, we are not really interested in the refined generating functions. They are just a useful bookkeeping device for the generating functions of the unrefined indices. Indeed, using the relation (228) and the fact that the contribution to the refined index of $\frac{1}{r}$-BPS states in a theory with N extended supersymmetry behaves as $O(z^{2K-2})$ where *K* is given by (225), one can obtain a relation between the generating functions of refined and unrefined indices, generalizing (126) valid in the (N|r)=(2|2) case,

(230) $$h_{p,\mu }^{\left(N|r\right)}\left(\tau \right)=\sum_{{\hat{q}}_{0}\leq {\hat{q}}_{0}^{\max}}{\bar{\Omega }}_{p,\mu }^{\left(N|r\right)}\left({\hat{q}}_{0}\right)\;q^{-{\hat{q}}_{0}}=\frac{2i{\left(2\pi \right)}^{3-2K}}{(2K-2)!}\;\partial_{z}^{2K-2}\left(zh_{p,\mu }^{\mathrm{ref}}\left(\tau ,z\right)\right){|}_{z=0}.$$

Here we introduced

(231) $${\bar{\Omega }}^{\left(N|r\right)}\left(\gamma \right)=\sum_{d|\gamma }d^{2K-4}\;\Omega^{\left(N|r\right)}\left(\gamma /d\right),$$

which is a generalization of (59). Interestingly, for N>2, one has K≥2 so that the indices (231) are *not* rational, although still different from $\Omega^{\left(N|r\right)}\left(\gamma \right)$ for non-primitive charges.

The relation (230) applied to the corresponding completions implies that ${\hat{h}}_{p,\mu }^{\left(N|r\right)}$ are modular forms of weight

(232) $$w\left(p\right)=2N\left(1-\frac{1}{r}\right)-\frac{1}{2}\;\mathrm{rank}\left(\Lambda_{p}\right)-3.$$

Their holomorphic anomaly equation can be derived by applying the differential operator (230) to the anomaly equation of ${\hat{h}}_{p,\mu }^{\mathrm{ref}}$ and taking the limit z→0. Taking again into account that $\Omega \left(\gamma ,y\right)=O\left(z^{2K-2}\right)$, one finds that the contributions of most BPS states to the anomaly simply disappear in the limit and one arrives at the following conclusions:

- The holomorphic anomaly equation can be non-trivial, and hence the generating functions can be mock modular, only for $\frac{1}{N}$-BPS states.
- Only $\frac{1}{2}$-BPS states can contribute to the r.h.s. of the holomorphic anomaly equation.
- For N>2 only the contribution of $\frac{1}{2}$-BPS states with n=2 survives the unrefined limit.

If one associates the existence of contributions to the anomaly equation to the existence of bound states, these conclusions would translate to the well-known fact that in theories with N>2 the only existing bound states are $\frac{1}{N}$-BPS states consisting of two $\frac{1}{2}$-BPS states.

To summarize, the conjecture 2 that the refined generating functions $h_{p,\mu }^{\mathrm{ref}}\left(\tau ,z\right)$ satisfy the same anomaly equations in theories with any number of extended supersymmetries implies that the generating functions $h_{p,\mu }^{\left(N|r\right)}\left(\tau \right)$ with r<N are vector valued modular forms of weight (232), whereas for r=N their modular completions satisfy

(233) $$\partial_{\bar{\tau }}{\hat{h}}_{p,\mu }^{\left(N|N\right)}\left(\tau ,\bar{\tau }\right)=q^{\frac{1}{2}\;\kappa^{AB}q_{A}q_{B}}\sum_{q_{0}}\sum_{\gamma_{1}+\gamma_{2}=\gamma }\;\;J_{2}\left(\left\{{\hat{\gamma }}_{1},{\hat{\gamma }}_{2}\right\},\tau_{2}\right)\prod_{i=1}^{2}\left({\bar{\Omega }}^{\left(N|2\right)}\left(\gamma_{i}\right)\;q^{-q_{i,0}}\right),$$

where $\gamma =(0,p^{A},q_{A},q_{0})$ and $q_{A}=\mu_{A}+\frac{1}{2}\kappa_{AB}p^{B}$. Note that we expressed the generating functions on the r.h.s. through the sum over D0-brane charges because BPS states differing only by this charge may preserve different number of supersymmetries.

#### 8.3.3. N=4

Let us apply the above results to string compactifications with N=4 supersymmetry. They can be realized either as type II string theory on $Y=K3\times T^{2}$ or as heterotic string theory on $T^{6}$.

The manifold Y is characterized by the following data

(234) $$b_{1}=2,\;b_{2}=23,\;c_{2,a}p^{a}=24p^{♭},$$

where the index ♭ corresponds to the divisor $D_{♭}=\left[K3\right]$. Thus, the indices A,B,… run over $b_{2}+2b_{1}=27$ values A∈{♭,α}={♭,1,…,26}, and the non-vanishing components of the symmetric tensor $\kappa_{ABC}$ are given by

(235) $$\kappa_{♭\alpha \beta }=\eta_{\alpha \beta }=\left(\begin{array}{cc} I_{1,1}^{⊕5} & 0\\ 0 & -C_{16} \end{array}\right),\;I_{1,1}=\left(\begin{array}{cc} 0 & 1\\ 1 & 0 \end{array}\right),$$

where $C_{16}$ is the Cartan matrix of $E_{8}\times E_{8}$.

The symmetries and BPS states are more easily characterized in the heterotic frame. The full U-duality group is a product of S and T-duality factors, SL(2,Z)×O(6,22;Z), and the electromagnetic charge vector is an SL(2,Z) doublet of two vectors under the T-duality group

(236) $$\gamma =\left(\begin{array}{c} Q_{I}\\ P_{I} \end{array}\right)=\left(\begin{array}{ccc} q_{0}, & -p^{♭}, & q_{\alpha }\\ q_{♭}, & p^{0}, & \eta_{\alpha \beta }p^{\beta } \end{array}\right),$$

where in the second representation we expressed the charge components in terms of the usual type IIA notations. There are two types of BPS states in this theory.

##### $\frac{1}{2}$-BPS States

$\frac{1}{2}$-BPS states are characterized by charges such that Q‖P and hence for each of them there is a duality frame where *P* can be set to zero. Since BPS indices should be invariant under the action of T-duality on the charges, the index $\Omega^{\left(4|2\right)}\left(\gamma \right)$ depends just on a single quantum number

(237) $$n=\frac{1}{2}\;Q^{2}=\frac{1}{2}\;Q_{I}\eta^{IJ}Q_{J},\;\eta_{IJ}=\left(\begin{array}{cc} I_{1,1}^{⊕6} & 0\\ 0 & -C_{16} \end{array}\right).$$

If we restrict to the D4-D2-D0 $\frac{1}{2}$-BPS states, there are two distinct cases to be considered. First, if $p^{♭}>0$, then all charges in the second line of (236) must vanish. Restricting for simplicity to $p^{♭}=1$, one obtains the following set of charges and the associated quadratic form

(238) $$\gamma_{1}=\left(\begin{array}{ccc} q_{0}, & -1, & q_{\alpha }\\ 0, & 0, & 0 \end{array}\right),\;\kappa_{AB}=\left(\begin{array}{cc} 0 & 0\\ 0 & \eta_{\alpha \beta } \end{array}\right).$$

Taking into account that for this set ${\hat{q}}_{0}=-n$, ${\hat{q}}_{0}^{\max}=1$, while $\mathrm{rank}(\kappa_{AB})=26$ and $\eta_{\alpha \beta }$ is unimodular, one finds that the generating function of $\frac{1}{2}$-BPS indices corresponding to the magnetic charge $p^{A}=\left(1,0,\ldots ,0\right)$ should read

(239) $$h_{p}^{\left(4|2\right)}\left(\tau \right)=\sum_{n=-1}^{\infty }\Omega^{\left(4|2\right)}\left(n\right)\;q^{n}$$

and be a modular form of weight −12 and trivial multiplier system. This immediately implies that it should be proportional to the inverse discriminant function (see Example 2), $h_{p}^{\left(4|2\right)}\left(\tau \right)∼\Delta^{-1}\left(\tau \right)$. This nicely agrees with the well-known fact that the two functions are actually equal [172].

Although the generating function (239) encodes all $\frac{1}{2}$-BPS indices, it is instructive to see how other $\frac{1}{2}$-BPS charges fit our formalism. The second possibility to get a D4-D2-D0 $\frac{1}{2}$-BPS state is to take $p^{♭}=0$ and other components in the two lines proportional to each other, i.e.,

(240) $$\gamma_{2}=\left(\begin{array}{ccc} \frac{\epsilon }{d_{Q}}\;q_{♭}, & 0, & \frac{\epsilon }{d_{Q}}\;\eta_{\alpha \beta }p^{\beta }\\ q_{♭}, & 0, & \eta_{\alpha \beta }p^{\beta } \end{array}\right),\;\kappa_{AB}=\left(\begin{array}{cc} 0 & \eta_{\alpha \beta }p^{\beta }\\ \eta_{\alpha \beta }p^{\beta } & 0 \end{array}\right),$$

where ϵ∈Z and $d_{Q}=\gcd\left(q_{♭},\left\{p^{\alpha }\right\}\right)$. In this case the charge $q_{0}$ is not independent, being fixed by other charges. This fact makes the generating series trivial since, instead of a sum over ${\hat{q}}_{0}$, there is only one term. One can show that it has ${\hat{q}}_{0}=0$ and hence the corresponding function is a constant. Since $\mathrm{rank}(\kappa_{AB})=2$, this agrees with the vanishing of the expected modular weight (232).

##### $\frac{1}{4}$-BPS States

$\frac{1}{4}$-BPS states are characterized by charges (236) with *Q* and *P* non-parallel, and their BPS indices depend on three T-duality invariants $\left(n,m,\ell \right)=\left(\frac{1}{2}Q^{2},\frac{1}{2}P^{2},Q\cdot P\right)$ and a U-duality invariant, known as torsion [173]

(241) $$I\left(\gamma \right)=\gcd\left\{Q_{I}P_{J}-Q_{J}P_{I}\right\},$$

so that one can write $\Omega^{\left(4|4\right)}\left(\gamma \right)=\Omega_{I}^{\left(4|4\right)}\left(n,m,\ell \right)$. The famous result of [4] is that the generating function of these indices for I=1 is a Seigel modular form with respect to Sp(2,Z) given by the inverse of the so-called Igusa cusp form $\Phi_{10}\left(\tau ,z,\sigma \right)$. For generic torsion, the indices $\Omega_{I}^{\left(4|4\right)}\left(n,m,\ell \right)$ have been found in [174] and can be expressed through those with I=1:

(242) $$\Omega_{I}^{\left(4|4\right)}\left(n,m,\ell \right)=\sum_{d|I}d\;\Omega_{1}^{\left(4|4\right)}\left(n,\frac{m}{d^{2}},\frac{\ell }{d}\right).$$

The coefficients $\psi_{m}\left(\tau ,z\right)$ of the expansion of $\Phi_{10}^{-1}$ in σ, the variable conjugate to the quantum number *m*, are Jacobi forms of weight −10 and index *m* with respect to SL(2,Z). A remarkable fact discovered in [16] is that they admit a canonical decomposition

(243) $$\psi_{m}=\psi_{m}^{P}+\psi_{m}^{F},$$

where $\psi_{m}^{P}$ contains the “polar" part of the original function and describes contributions of bound states only, whereas all contributions of single centered black holes (immortal dyons) are encoded in $\psi_{m}^{F}$, which does not have any poles in *z*. Furthermore, both these functions are mock modular and the modular completion of the generating function of immortal dyons satisfies

(244) $$\tau_{2}^{3/2}\partial_{\bar{\tau }}{\hat{\psi }}_{m}^{F}\left(\tau ,\bar{\tau },z\right)=\frac{\sqrt{m}}{8\pi i}\;\frac{\Omega^{\left(4|2\right)}\left(m\right)}{\Delta (\tau )}\sum_{\ell =0}^{2m-1}\bar{\theta_{\ell }^{\left(m\right)}\left(\tau ,0\right)}\;\theta_{\ell }^{\left(m\right)}\left(\tau ,z\right)\equiv A_{m}\left(\tau ,\bar{\tau },z\right),$$

where $\theta_{\ell }^{\left(m\right)}\left(\tau ,z\right)$ is the theta series (29).

Let us again restrict to D4-D2-D0 states by setting $p^{0}=0$ in (236). In addition, for simplicity, we assume $p^{♭}=1$ and $p^{2}>0$. Then the T and U-duality invariants are found to be

(245) $$n=\frac{1}{2}\;q^{2}-q_{0},\;m=\frac{1}{2}\;p^{2},\;\ell =p^{\alpha }q_{\alpha }-q_{♭},\;I\left(\gamma \right)=\gcd\left(\ell ,\left\{p^{\alpha }\right\}\right),$$

where $q^{2}=\eta^{\alpha \beta }q_{\alpha }q_{\beta }$ and $p^{2}=\eta_{\alpha \beta }p^{\alpha }p^{\beta }$, while the quadratic form is non-degenerate and is given by

(246) $$\kappa_{AB}=\left(\begin{array}{cc} 0 & \eta_{\alpha \beta }p^{\beta }\\ \eta_{\alpha \beta }p^{\beta } & \eta_{\alpha \beta } \end{array}\right),$$

with $|\;\det\;\kappa_{AB}|=2m$. Since the indices defining the generating function $h_{p,\mu }^{\left(4|4\right)}$ are evaluated at the attractor point, they count only single-centered black holes. (More precisely, this statement holds for terms with negative ${\hat{q}}_{0}$, which count BPS black holes with non-vanishing area. In principle, at the attractor point, also the scaling solutions [23,80] might contribute but, being composed of at least three constituents, they do not exist in N=4 theory [175]). Therefore, after multiplication by ${\left(-1\right)}^{\ell }\theta_{\ell }^{\left(m\right)}\left(\tau ,z\right)$ (the sign factor is needed to cancel the multiplier system of $h_{p,\mu }^{\left(4|4\right)}$ and nicely agrees with the presence of the same factor in the definition of the generating function given by the Igusa cusp form, which was advocated in [176]) and summing over ℓ=m−μ, it should coincide with a generalization $\psi_{p}$ of the generating function of immortal dyons $\psi_{m}^{F}$ to an arbitrary torsion invariant. According to (232), its weight is expected to be $w\left(p\right)+\frac{1}{2}=-10$, while its index is equal to *m*, in agreement with the weight and index of $\psi_{m}^{F}$.

The most interesting is to compare their anomaly equations. According to (233), the non-vanishing contributions to the holomorphic anomaly equation for ${\hat{\psi }}_{p}$ arise only from splits of the charge $\gamma =\gamma_{1}+\gamma_{2}$ where $\gamma_{1}$ and $\gamma_{2}$ are both $\frac{1}{2}$-BPS charges. It is easy to see that this is possible only if one of them belongs to the class (238) and the other to (240) with $d_{Q}=I\left(\gamma \right)$. Moreover, all charges of the constituents are fixed in terms of the full charge and a single integer parameter, so that the lattice one sums over on the r.h.s. of (233) is one-dimensional. Evaluating all the ingredients explicitly and taking into account that $h_{p}^{\left(4|2\right)}=\Delta^{-1}$, one can show [33] that the anomaly Equation (233) is equivalent to

(247) $$\tau_{2}^{3/2}\partial_{\bar{\tau }}\;{\hat{\psi }}_{p}\left(\tau ,\bar{\tau },z\right)=\sum_{d|d_{p}}d\;A_{m/d^{2}}\left(\tau ,\bar{\tau },dz\right),$$

where $d_{p}\equiv \gcd\left\{p^{\alpha }\right\}$. If $d_{p}=1$, which implies the trivial torsion I(γ)=1, this equation reproduces (244). For $d_{p}>1$, it provides a generalization of the anomaly equation found in [16] to the case of a non-trivial torsion. One can also check that it is consistent with the relation (242).

It is worth emphasizing that the function $\psi_{p}$ for $d_{p}>1$ comprises contributions of charges with *different* values of the torsion invariant. In fact, it includes states with all *I* dividing $d_{p}$. Therefore, it is different from the generating function of $\frac{1}{4}$-BPS indices with fixed I>1, which is known to transform properly only under the congruence subgroup $\Gamma^{0}\left(I\right)$ of SL(2,Z) [174]. Instead, the presented construction automatically produces functions that transform as (mock) modular or Jacobi forms under the full SL(2,Z). In other words, it tells us how charges with different torsion should be combined together in order to form objects with nice modular properties—if one follows the general prescription, the result is guaranteed.

#### 8.3.4. N=8

String compactifications with N=8 supersymmetry are obtained by taking type II string theory on $T^{6}$. In this case $b_{1}=6$ and $b_{2}=15$, so that the indices A,B,… run again over $b_{2}+2b_{1}=27$ values. We will denote the charge components as $p^{A}=\left(p^{ij},p_{i},{\tilde{p}}_{i}\right)$ and $q_{A}=\left(q_{ij},q^{i},{\tilde{q}}^{i}\right)$, where i=1,…,6 and $q_{ij}$ and $p^{ij}$ are antisymmetric. The antisymmetric components correspond to the gauge fields coming from the RR sector, while the charges with one index correspond to the gauge fields arising from the reduction of the metric and the *B*-field on one-cycles.

The U-duality group is $E_{7}$ and the charge vector transforms in the irrep 56 of this group. The U-duality orbits are characterized by a single quartic invariant $I_{4}\left(\gamma \right)$, which implies that the BPS indices depend on a single quantum number. The invariant $I_{4}\left(\gamma \right)$ can be written in terms of a cubic invariant of $E_{6}$ appearing in the reduction $E_{7}\to E_{6}\times O\left(1,1\right)$ [177]

(248) $$I_{4}\left(\gamma \right)=4p^{0}I_{3}\left(q\right)-4q_{0}I_{3}\left(p\right)+4\partial^{A}I_{3}\left(q\right)\;\partial_{A}I_{3}\left(p\right)-{\left(p^{0}q_{0}+p^{A}q_{A}\right)}^{2},$$

which itself is given by

(249) $$I_{3}\left(p\right)=Pf\left(p^{ij}\right)+p^{ij}p_{i}{\tilde{p}}_{j},\;Pf\left(p^{ij}\right)=\frac{1}{48}\;\epsilon_{ijklmn}p^{ij}p^{kl}p^{mn}.$$

In particular, it is the cubic invariant that defines the quadratic form relevant for D4-D2-D0 BPS states,

(250) $$\kappa_{AB}=\partial_{A}\partial_{B}I_{3}\left(p\right).$$

For vanishing NS-charges, it is easy to compute it explicitly

(251) $$\kappa_{AB}=\left(\begin{array}{ccc} \frac{1}{2}\;\epsilon_{ijklmn}p^{mn} & 0 & 0\\ 0 & 0 & p^{rs}\\ 0 & p^{rs} & 0 \end{array}\right),$$

which gives, in particular,

(252) $$|\;\det\;\kappa_{AB}|=2{\left(\mathrm{Pf}\left(p^{ij}\right)\right)}^{9},\;{\hat{q}}_{0}=-\frac{I_{4}\left(\gamma \right)}{4I_{3}\left(p\right)}\;.$$

The theory has three types of BPS states and there are many charge configurations corresponding to D4-D2-D0 bound states realizing them. In the following, we will consider only a few representative examples to demonstrate how the formalism of Section 8.3.2 reproduces the well-known modular properties of the BPS indices.

##### $\frac{1}{2}$-BPS States

The BPS conditions on charge vectors have been found in [178] and can be written in terms of the quartic invariant and the derivatives $\partial_{I}$ with respect to charges where I labels all components of the charge vector γ. The $\frac{1}{2}$-BPS condition is the strongest one and reads as

(253) $$\partial_{I}\partial_{J}I_{4}{\left(\gamma \right)|}_{\mathrm{Adj}(E_{7})}=0,$$

where $\mathrm{Adj}(E_{7})$ denotes the representation 133 appearing in the decomposition $56^{2}=133+1463$. Note that it implies the vanishing of $I_{4}\left(\gamma \right)$. It has been elaborated in full generality in terms of charge components in [179]. Instead, let us take the only non-vanishing magnetic charges to be $p^{12},p^{34},p^{56}$. One can show that the condition (253) implies that there can be at most one non-vanishing magnetic charge, so we take $p^{12}\neq 0$, $p^{34}=p^{56}=0$. Then the $\frac{1}{2}$-BPS condition leaves only 10 unrestricted electric charges, consistently with the fact that $\mathrm{rank}(\kappa_{AB})=10$. Hence, the expected modular weight (232) of $h_{p}^{\left(8|2\right)}$ is equal to 0, i.e., the generating function of $\frac{1}{2}$-BPS D4-D2-D0 states must be a constant. And indeed the condition (253) fixes $q_{0}$ so that ${\hat{q}}_{0}=0$. All this nicely agrees with the fact (see, e.g., [180,181]) that there is a single $\frac{1}{2}$-BPS index equal to 1.

##### $\frac{1}{4}$-BPS States

The case of $\frac{1}{4}$-BPS states is very interesting because it illustrates several non-trivial phenomena. First, this is the case where BPS indices have a completely different behavior compared to the degeneracies counted without the sign insertion. The latter are counted by

(254) $$h_{\deg}^{\left(8|4\right)}\left(\tau \right)=\frac{1}{16}\prod_{n=1}^{\infty }{\left(\frac{1+q^{n}}{1-q^{n}}\right)}^{8}=\frac{1}{16}{\left(\frac{\eta (2\tau )}{\eta^{2}\left(\tau \right)}\right)}^{8}.$$

Note that the first term in the Fourier expansion is given by rational number $\frac{1}{16}$ because it corresponds to $\frac{1}{2}$-BPS states and is equal to the ratio of the dimensions of the ultrashort $\frac{1}{2}$-BPS multiplet (${\left(16\right)}^{2}$ states) and the short $\frac{1}{4}$-BPS multiplet (${\left(16\right)}^{3}$ states). The resulting degeneracies grow exponentially with *n*. On the other hand, the BPS indices are organized into the generating function ([181], Eq.(2.13))

(255) $$h^{\left(8|4\right)}\left(\tau \right)=\frac{E_{4}\left(\tau \right)}{240}+\frac{7}{144}$$

and grow only polynomially. Let us see how this result can be recovered from the approach used above.

The $\frac{1}{4}$-BPS condition is given by

(256) $$\partial_{I}I_{4}\left(\gamma \right)=0,\;\partial_{I}\partial_{J}I_{4}{\left(\gamma \right)|}_{\mathrm{Adj}E_{7}}\neq 0,$$

which also implies the vanishing of $I_{4}\left(\gamma \right)$. Let us again restrict to the case where the only non-vanishing magnetic charges are $p^{12},p^{34},p^{56}$. Then the condition (256) requires that at least one of these charges must vanish. If one considers the most natural possibility $p^{12},p^{34}\neq 0$, $p^{56}=0$, one finds that it is similar to the case of $\frac{1}{2}$-BPS states discussed above because the $\frac{1}{4}$-BPS condition fixes the D0-brane charge so that ${\hat{q}}_{0}=0$ and the generating function reduces to a constant. This is consistent with the fact that $\mathrm{rank}(\kappa_{AB})=18$, which implies the vanishing of the modular weight (232).

A non-trivial generating function is obtained in a more degenerate case of $p^{12}\neq 0$, $p^{34}=p^{56}=0$ because $q_{0}$ is then left unrestricted. Since now $\mathrm{rank}(\kappa_{AB})=10$, the modular weight of $h_{p}^{\left(8|4\right)}$ should be equal to 4. One can also verify the triviality of the multiplier system and that ${\hat{q}}_{0}^{\max}=0$, which singles out a unique modular form $E_{4}\left(\tau \right)$ satisfying all these requirements, consistently with (255). The deviation from the Eisenstein series is due to the fact, already noticed below (254), that the constant term corresponding to ${\hat{q}}_{0}=0$ counts $\frac{1}{2}$-BPS states and not $\frac{1}{4}$-BPS. Importantly, it does *not* generate a holomorphic anomaly for the modular completion ${\hat{h}}_{p}^{\left(8|4\right)}$ because the completing term is holomorphic being just a constant. Its precise value can be obtained as

(257) $$\frac{B_{12}\left(R_{0,8}\right)}{B_{12}\left(R_{0,12}\right)}=\frac{1}{12!}{\left(y\partial_{y}\right)}^{12}{\left[{\left(1-y\right)}^{4}{\left(1-y^{-1}\right)}^{4}\right]}_{y=1}=\frac{19}{360}$$

and agrees with (255) due to $\frac{1}{240}+\frac{7}{144}=\frac{19}{360}$.

##### $\frac{1}{8}$-BPS States

The last case of $\frac{1}{8}$-BPS states has been studied in many works (see, e.g., [177,182,183,184]), and it was found that the $\frac{1}{8}$-BPS indices are given by

(258) $$\Omega^{\left(8|8\right)}\left(\gamma \right)=\sum_{s\;:\;\nabla_{X}F_{0}\in Z}s\;N\left(s\right)\;\hat{c}\left(\frac{I_{3}\left(Q\right)}{s^{2}}\;,\;\frac{J_{L}}{s}\right)=\sum_{2s|\chi (\gamma )}s\;\hat{c}\left(I_{4}\left(\gamma \right)/s^{2}\right),$$

where in the first representation N(s) is the number of common divisors of $X^{I}$ and $\partial_{I}F_{0}$ with

(259) $$F_{0}\left(X\right)=\frac{I_{3}\left(X\right)}{X^{0}}\;,\;X^{I}=\left(s,q_{A}\right),$$

and

(260) $$Q_{A}=p^{0}q_{A}+\partial_{A}I_{3}\left(p\right),\;2J_{L}={\left(p^{0}\right)}^{2}q_{0}+p^{0}p^{A}q_{A}+2I_{3}\left(p\right).$$

The second representation is manifestly U-duality invariant, and we refer to [184] for the precise definition of the function χ(γ). The most important ingredient in this formula is provided by $\hat{c}\left(n,\ell \right)$, the Fourier coefficients of the Jacobi form of weight −2 and index 1

(261) $$\phi \left(\tau ,z\right)=-\frac{\theta_{1}{\left(\tau ,z\right)}^{2}}{\eta {\left(\tau \right)}^{6}}=\sum_{n=0}^{\infty }\sum_{\ell \in Z}\hat{c}\left(n,\ell \right)\;q^{n}\;y^{\ell }.$$

The coefficients actually depend on a single variable

(262) $$\hat{c}\left(n,\ell \right)=\hat{c}\left(4n-\ell^{2}\right),$$

which is used in the second representation in (258). It turns out that in this form, they coincide with the Fourier coefficients of the following function

(263) $$\Phi \left(\tau \right)=\frac{\theta_{4}\left(2\tau \right)}{\eta {\left(4\tau \right)}^{6}}=\sum_{n=-1}^{\infty }\hat{c}\left(n\right)\;q^{n}\;.$$

However, Φ(τ) is not the function we are looking for because it is *not* modular with respect to the full SL(2,Z). The reason is that the Jacobi form (261) has index 1 and hence implies that its coefficients can be combined into a *vector valued* modular form with two components by means of the theta expansion as in (28):

(264) $$\phi \left(\tau ,z\right)=\sum_{\ell =0,1}{\left(-1\right)}^{\ell }h_{\ell }\left(\tau \right)\;\theta_{\ell }^{\left(1\right)}\left(\tau ,z\right),$$

where $\theta_{0}^{\left(1\right)}\left(\tau ,z\right)=\theta_{3}\left(2\tau ,2z\right)$, $\theta_{1}^{\left(1\right)}\left(\tau ,z\right)=\theta_{2}\left(2\tau ,2z\right)$ and for convenience we included a sign factor that affects only the multiplier system of $h_{\ell }\left(\tau \right)$. The two components correspond to odd and even values of the quartic invariant $d=4n-\ell^{2}$ and are given by

(265) $$h_{\ell }\left(\tau \right)={\left(-1\right)}^{\ell }\sum_{d\in 4Z-\ell^{2}}\hat{c}\left(d\right)\;q^{d/4}=\left(\frac{\theta_{3}\left(2\tau \right)}{\eta {\left(\tau \right)}^{6}}\;,\;\frac{\theta_{2}\left(2\tau \right)}{\eta {\left(\tau \right)}^{6}}\right),$$

which is most easily obtained by decomposing (263):

(266) $$\Phi \left(\tau /4\right)=\frac{\theta_{4}\left(\tau /2\right)}{\eta {\left(\tau \right)}^{6}}=\frac{\theta_{3}\left(2\tau \right)}{\eta {\left(\tau \right)}^{6}}-\frac{\theta_{2}\left(2\tau \right)}{\eta {\left(\tau \right)}^{6}}.$$

The vector (265) does transform as a modular form under the full SL(2,Z) as follows from Example 4.

On the other hand, the general construction of Section 8.3.2 implies that we should consider

(267) $$h_{p,\mu }\left(\tau \right)=\sum_{I_{4}\in 4I_{3}\left(p\right)Z+I_{4}\left(\mu \right)}{\bar{\Omega }}^{\left(8|8\right)}\left(I_{4}\right)\;q^{I_{4}/4I_{3}\left(p\right)},$$

where we took into account the relation (252) and that the BPS indices depend only on the quartic invariant. Let us again restrict to the case where all magnetic charges vanish except $p^{12},p^{34},p^{56}$ and denote $m=I_{3}\left(p\right)=p^{12}p^{34}p^{56}$. Since the $\frac{1}{2}$-BPS condition requires vanishing of at least two of the charges $p^{12},p^{34},p^{56}$, it is impossible to decompose the magnetic charge $p^{A}$ into two charges giving rise to $\frac{1}{2}$-BPS states. Therefore, the r.h.s. of the holomorphic anomaly Equation (233) vanishes and the generating function (267) must be a vector-valued modular form. Its weight follows from (232). Since the quadratic form is non-degenerate with $\mathrm{rank}(\kappa_{AB})=27$, it is equal to −5/2. Furthermore, given that $c_{2,a}=0$ and $\left(p^{3}\right)=6I_{3}\left(p\right)=6m$, the most singular term has the power $-{\hat{q}}_{0}^{\max}=-\frac{m}{4}$, while due to (252), $\mu_{A}$ run over $2m^{9}$ values. For m=1, these properties reproduce those of the two-dimensional vector (265). One can also show that the multiplier systems also agree, and since all charges are primitive, ${\bar{\Omega }}^{\left(8|8\right)}\left(I_{4}\right)=\Omega^{\left(8|8\right)}\left(I_{4}\right)$. Thus, up to an overall scale, the generating function (267) must coincide with $h_{\ell }\left(\tau \right)$. Of course, for m>1, the generating function will be different, but it is constructed from the same set of BPS indices $\Omega^{\left(8|8\right)}\left(I_{4}\right)$ and hence carries the same information.

## 9. Conclusions

Mock modularity is a beautiful mathematical structure that represents now a rapidly developing and expanding subject of mathematical research with numerous and deep relations to theoretical physics. Its manifestations range from non-compact CFTs [185], sigma models [186] and black hole state counting in string compactifications [16,25,26] to Vafa–Witten theory [54,153], Donaldson–Witten theory [187,188], moonshine phenomenon [189], quantum invariants of three-dimensional manifolds [190,191] and many other setups. In this review we concentrated mainly on one of these manifestations—mock modularity of the generating functions of BPS indices counting states of supersymmetric black holes in Calabi–Yau compactifications and realized in mathematics as rank 0 generalized DT invariants. We showed that it governs a universal structure represented by an iterated system of anomaly equations. It is universal because it turns out to describe many phenomena beyond the original setup. It remains valid for various degenerations, in the non-compact limit, after inclusion of a refinement, and even for compactifications with higher supersymmetry, where it allows to reproduce most of the known results. This universality suggests that the same or a similar structure may also govern the other manifestations of mock modularity mentioned above, as has been shown, for instance, for the Vafa–Witten theory [28].

Despite the original argument for modularity of the generating functions of D4-D2-D0 BPS indices came from the analysis of a CFT living on the brane world-volume [3], it is the target space perspective that turned out to be more productive. In this physical picture, the origin of modularity can be traced back to S-duality of type IIB string theory, while the mock modularity appears to arise due to wall-crossing, i.e., the existence of bound states whose stability depends on values of the moduli. On the other hand, a pure mathematical understanding of both these phenomena for the generating functions of rank 0 DT invariants for generic CY threefolds is still absent, and only recently have first steps been undertaken in this direction in the simplest case of the quintic threefold and unit D4-brane charge [42].

The main application of the mock modular properties of the generating functions expressed by the system of anomaly equations is the actual computation of these generating series. Thus far, the work in this direction has concentrated on non-compact cases (VW theory) and one-parameter CY threefolds. It demonstrated that there is a nice interplay between mock modularity of rank 0 DT invariants and the holomorphic anomaly of topological strings, which compute GV invariants of the same CY threefold. Computing one set of invariants helps computing the other and vice versa. Proceeding in this way allows us to overcome the limitations of the direct integration approach to solving topological string theory on compact CYs.

However, to further pursue this idea and apply it to higher D4-brane charges and to more general CY threefolds with more moduli, we need new wall-crossing relations between various topological invariants, which would allow us to compute them more efficiently than the currently known relations. This might be seen as the key open problem of this research program. The most promising avenue seems to be the study of D6-$\bar{D6}$ wall crossing. However, the existing results in this direction are insufficient for applications. An intriguing workable prescription was proposed in [36], but it seems to be at odds with the standard mathematical definition of DT invariants and the standard wall-crossing formulas.

Although higher rank DT invariants are not expected to possess modular properties, one can still ask whether the presented results can help in computing them. In principle, according to the recent results [132,133,134,135,136], they all should be expressible through rank 0 invariants. Unfortunately, these results are not constructive yet, and there are no explicit formulas that would allow us to do so.

One could ask whether some of the results can be extended to theories with less supersymmetry, describing more realistic compactifications. Unfortunately, it is hard to expect such an extension since not only do BPS states not exist in such theories but also the fate of S-duality remains unclear [192]. Nevertheless, recently some inflation models have been proposed where modular symmetry plays a crucial role [193,194]. Mock modularity, however, did not show up yet in these investigations.

Finally, an almost unexplored subject is the interplay between mock modularity and non-commutativity suggested by the emergence of a non-commutative star-product structure on the moduli space (and its twistor space) after the inclusion of a refinement. What does this non-commutativity mean physically? What does it imply for the low-energy effective action? Why should it be compatible with S-duality? These are just a few questions that may be asked about this new, exciting playground for mock modularity.

## Figures and Tables

**Table 1:** Relevant data for the 13 hypergeometric CY threefolds. In the first column, we use the notation $X_{d_{1},\ldots ,d_{k}}\left(w_{1}^{m_{1}},\ldots ,w_{p}^{m_{p}}\right)$ to denote a complete intersection of multidegree $\left(d_{1},\ldots ,d_{k}\right)$ in weighted projective space $P^{k+3}\left(w_{1},\ldots ,w_{p}\right)$ where $m_{i}$ is the number of repetitions of the weight $w_{i}$. The second to fourth columns indicate the Euler number of Y, the self-intersection number κ, and the second Chern class $c_{2}$. The column $g_{\mathrm{integ}}$ gives the maximal genus for which GV invariants ${\mathrm{GV}}_{Q}^{\left(g\right)}$ can be determined by the direct integration method using only the usual boundary conditions. The column $g_{\mathrm{mod}}^{\left(r\right)}$ shows how this bound changes after adding information about the GV invariants predicted by the knowledge of $h_{r,\mu }$ for r=1 and in brackets, for the two manifolds where these generating series are available, for r=2. The column $g_{\mathrm{avail}}$ indicates the genus up to which complete tables of GV invariants are currently known. Finally, the columns $Q_{\mathrm{integ}}$ and $Q_{\mathrm{avail}}$ provide the maximal degrees for DT and PT invariants attainable through the direct integration and available now due to the additional information about the rank 0 DT invariants.

Y	$\chi_{Y}$	κ	$c_{2}$	$g_{\mathrm{integ}}$	$g_{\mathrm{mod}}^{\left(r\right)}$	$g_{\mathrm{avail}}$	$Q_{\mathrm{integ}}$	$Q_{\mathrm{avail}}$
$X_{5}\left(1^{5}\right)$	−200	5	50	53	69	64	22	26
$X_{6}\left(1^{4},2\right)$	−204	3	42	48	66	48	15	17
$X_{8}\left(1^{4},4\right)$	−296	2	44	60	84(112)	64	15	17
$X_{10}\left(1^{3},2,5\right)$	−288	1	34	50	70(95)	71	11	13
$X_{3,3}\left(1^{6}\right)$	−144	9	54	29	33	33	20	21
$X_{4,2}\left(1^{6}\right)$	−176	8	56	50	64	64	28	31
$X_{4,3}\left(1^{5},2\right)$	−156	6	48	20	24	24	14	15
$X_{6,2}\left(1^{5},3\right)$	−256	4	52	63	78	49	17	20
$X_{4,4}\left(1^{4},2^{2}\right)$	−144	4	40	26	34	34	14	16
$X_{6,4}\left(1^{3},2^{2},3\right)$	−156	2	32	14	17	17	7	8
$X_{6,6}\left(1^{2},2^{2},3^{2}\right)$	−120	1	22	18	21	24	6	7

## Data Availability

No new data were created or analyzed in this study.

## Appendix B. Generalized Error Functions

The generalized error functions have been introduced in [59,64] (see also [61,195]). They are defined by

(A1) $$\begin{array}{ccc} E_{n}\left(M;u\right) & = & \int_{R^{n}}du^{\prime }\;e^{-\pi \sum_{i=1}^{n}{\left(u_{i}-u_{i}^{\prime }\right)}^{2}}\prod_{i=1}^{n}\mathrm{sgn}{\left(M^{\mathrm{tr}}u^{\prime }\right)}_{i}\;, \end{array}$$

where $u=(u_{1},\ldots ,u_{n})$ is an *n*-dimensional vector and M is an n×n matrix of parameters. The information carried by M is in fact highly redundant. For example, for n=1, the dependence on M drops out, whereas $E_{2}$ and $E_{3}$ depend only on 1 and 3 parameters, respectively. The functions $E_{n}$ generalize the ordinary error function because at n=1, one has $E_{1}\left(u\right)=\mathrm{Erf}\left(\sqrt{\pi }\;u\right)$. Their main role is to provide modular completions of theta series with quadratic forms of indefinite signature.

To get kernels of indefinite theta series, we need, however, functions depending on a *d*-dimensional vector rather than an *n*-dimensional one. To define such functions, let $\left\{v_{i}\right\}$ be a set of *n* vectors of dimension *d*, and it is assumed that these vectors span a positive definite subspace in $R^{d}$ endowed with a bilinear form ∗, i.e., $v_{i}*v_{j}$ is a positive definite matrix. (This bilinear form is *opposite* to the bilinear form ★ used in the beginning of Section 3 up to Equation (42), see also Remark 1. In fact, the sign of the bilinear form affects just the overall sign of $\Phi_{n}^{E}$). We also introduce an orthonormal basis $B=\{e_{i}\}$ for this subspace. Then, we set

(A2) $$\Phi_{n}^{E}\left(\left\{v_{i}\right\};x\right)=E_{n}\left(M;B*x\right),\;M_{ij}=e_{i}*v_{j}.$$

The detailed properties of these functions can be found in [59]. The most important of them are the following:

- The functions $\Phi_{n}^{E}\left(\left\{v_{i}\right\};x\right)$ do not depend on the choice of the basis B and are smooth functions of x.
- They solve the Vignéras Equation (43) with λ=0.
- At large x, they reduce to $\prod_{i=1}^{n}\mathrm{sgn}\left(v_{i}*\;x\right)$.
- Their derivative is given by
  (A3) $$\partial_{x}\Phi_{n}^{E}\left(\left\{v_{i}\right\};x\right)=2\sum_{k=1}^{n}\frac{v_{k}}{\left|\right|v_{k}\left|\right|}\;e^{-\pi \;\frac{{\left(v_{k}*x\right)}^{2}}{v_{k}^{2}}}\;\Phi_{n-1}^{E}\left({\left\{v_{i}\right\}}_{i\neq k};x\right).$$
- If one of the vectors is null, it reduces the rank of the generalized error function. Namely, for $v_{\ell }^{2}=0$, one has
  (A4) $$\Phi_{n}^{E}\left(\left\{v_{i}\right\};x\right)=\mathrm{sgn}\left(v_{\ell }\;*x\right)\;\Phi_{n-1}^{E}\left({\left\{v_{i}\right\}}_{i\neq \ell };x\right).$$
  In other words, for such vectors, the completion is not required.

We also need a second set of functions that can be seen as generalizations of the complementary error function. They were also defined in [59,64], but we will use a slightly generalized version introduced in [28] that depends on an additional vector of parameters:

(A5) $${\hat{M}}_{n}\left(M;u,b\right)={\left(\frac{i}{\pi }\right)}^{n}{\left|\;\det\;M\right|}^{-1}\int_{R^{n}-i\left(u-b\right)}du^{\prime }\;\frac{e^{-\pi u^{\prime \mathrm{tr}}u^{\prime }-2\pi iu^{\prime \mathrm{tr}}u}}{\prod_{i=1}^{n}{\left(M^{-1}u^{\prime }\right)}_{i}}\;.$$

For n=1, one recovers the ordinary complementary error function, ${\hat{M}}_{1}\left(u,0\right)=-\mathrm{sgn}\left(u\right)\mathrm{Erfc}(\sqrt{\pi }\left|u|\right)$, and more generally, ${\hat{M}}_{n}$ are exponentially suppressed for large u as ${\hat{M}}_{n}∼\frac{{\left(-1\right)}^{n}}{\pi^{n}}\;{\left|\;\det\;M\right|}^{-1}\frac{e^{-\pi u^{\mathrm{tr}}u}}{\prod_{i=1}^{n}{\left(M^{-1}u\right)}_{i}}$. In turn, these functions are used to define

(A6) $${\hat{\Phi }}_{n}^{M}\left(\left\{v_{i}\right\};x,b\right)={\hat{M}}_{n}\left(M;B\cdot x,B*b\right),$$

where the matrices M and B are the same as in (A2), which can be used as kernels of indefinite theta series. More precisely, ${\hat{\Phi }}_{n}^{M}$ can be seen as iterated Eichler integrals providing modular completions of such theta series. Note that, in contrast to $\Phi_{n}^{E}$, they are smooth only away from real codimension-1 loci in $R^{d}$.

An important fact is that the functions $\Phi_{n}^{E}$ and ${\hat{\Phi }}_{n}^{M}$ can be expressed through each other and sign functions. More precisely, they satisfy the following identity ([28], Prop. 1):

(A7) $$\Phi_{n}^{E}\left(\left\{v_{i}\right\};x\right)=\sum_{I\subseteq Z_{n}}{\hat{\Phi }}_{\left|I\right|}^{M}\left({\left\{v_{i}\right\}}_{i\in I};x,b\right)\prod_{j\in Z_{n}\setminus I}\mathrm{sgn}\left(v_{j⊥I}*\left(x-b\right)\right),$$

where the sum goes over all possible subsets (including the empty set) of the set $Z_{n}=\left\{1,\ldots ,n\right\}$, |I| is the cardinality of I, and $v_{j⊥I}$ denotes the projection of $v_{j}$ orthogonal to the subspace spanned by ${\left\{v_{i}\right\}}_{i\in I}$. This identity should also make clear the role of the vector b: if  as in (42), choosing , one obtains that the sign functions in (A7) are independent of. This is important for the refined construction in Section 6.3 where the constant contributions $E_{n}^{(0)\mathrm{ref}}$ (123) are defined by setting β=0.

## Appendix C. Lattice Factorization and Theta Series

In this appendix, we recall some basic facts about the decomposition of a lattice into sublattices and the corresponding factorization of theta series (see [196] for more details).

Let  be a lattice of dimension *d* and , a=1,…,n, are orthogonal sublattices of dimensions $d_{a}$ such that $\sum_{a=1}^{n}d_{a}=d$. The problem that we want to address here is that, in general,  is only a sublattice of . While all elements of  can be decomposed as linear combinations of elements of , the decompositions may involve rational coefficients. In such a situation, to get the full lattice from the sublattices, one has to introduce the so-called *glue vectors*. They are given by the sum of representatives of the discriminant groups , which at the same time belongs to the original lattice, i.e.,  where $g_{A}^{\left(a\right)}\in D^{\left(a\right)}$. The number of the glue vectors is equal to

(A8)

where  is the order of the discriminant group and is equal to the determinant of the matrix of scalar products of the basis elements. The decomposition formula of the lattice  then reads

(A9)

The main application of the lattice decomposition (A9) is a factorization of theta series. Let us consider a general indefinite theta series as in (42) with a kernel having a factorized form $\Phi \left(x\right)=\prod_{a=1}^{n}\Phi_{a}\left(x^{\left(a\right)}\right)$ where the upper index ^(*a*)^ on a vector denotes its projection to . Then the lattice factorization Formula (A9) implies that one can split the sum in the definition of the theta series into *n* sums coupled by the additional sum over the glue vectors, so that one arrives at the following identity for theta series

(A10)

## Appendix D. Degenerate Case with Non-Degenerate Quadratic Form

In this appendix, we compute the holomorphic anomaly of the modular completion (92) in the degenerate case where $(p_{0}^{3})=0$.

Before we specialize (92) to the degenerate case, let us rewrite it in a more explicit form by solving the constraint on the D2-brane charges

(A11) $$q_{1,a}+q_{2,a}=\mu_{a}+\frac{1}{2}\;r^{2}\kappa_{0,ab}p_{0}^{b}$$

where $\kappa_{0,ab}=\kappa_{abc}p_{0}^{c}$ and $q_{i,a}$ are decomposed as in (57) with $p_{i}^{a}=r_{i}p_{0}^{a}$. In terms of the spectral flow parameters $\epsilon_{i}^{a}$, the constraint (A11) takes the form

(A12) $$\kappa_{0,ab}\left(r_{1}\epsilon_{1}^{b}+r_{2}\epsilon_{2}^{b}\right)=\Delta \mu_{a}+r_{1}r_{2}\kappa_{0,ab}p_{0}^{b},$$

where

(A13) $$\Delta \mu_{a}=\mu_{a}-\mu_{1,a}-\mu_{2,a}.$$

An immediate consequence of this relation is that $\Delta \mu \in r_{0}\Lambda_{0}$, where $r_{0}=\gcd\left(r_{1},r_{2}\right)$ and the lattice $\Lambda_{0}$ is endowed with the quadratic form $\kappa_{0,ab}$. Furthermore, let $\rho_{1}^{a}$ and $\rho_{2}^{a}$ be any two integer-valued vectors satisfying

(A14) $$\kappa_{0,ab}\left(r_{1}\rho_{1}^{b}+r_{2}\rho_{2}^{b}\right)=\Delta \mu_{a}+r_{1}r_{2}\kappa_{0,ab}p_{0}^{b}.$$

Then, the general solution to (A12) is given by

(A15) $$\epsilon_{1}^{a}=\rho_{1}^{a}+\frac{r_{2}}{r_{0}}\;\epsilon^{a},\;\epsilon_{2}^{a}=\rho_{2}^{a}-\frac{r_{1}}{r_{0}}\;\epsilon^{a},\;\epsilon^{a}\in Z.$$

Substituting this result into the spectral flow decomposition of $q_{i,a}$, it is easy to compute

(A16) $$\gamma_{12}=r_{0}p_{0}^{a}k_{a},\;Q_{2}\left({\hat{\gamma }}_{i},{\hat{\gamma }}_{2}\right)=-\frac{k^{2}}{r_{12}}\;,$$

where we denoted $r_{12}=rr_{1}r_{2}/r_{0}^{2}$ and

(A17) $$\begin{array}{cc} k_{a}\left(\epsilon \right)= & \;r_{12}\kappa_{0,ab}\epsilon^{b}+\mu_{12,a},\;k^{2}=\kappa_{0}^{ab}k_{a}k_{b},\\ \mu_{12,a}= & \;\frac{1}{r_{0}}\left(r_{2}\mu_{1,a}-r_{1}\mu_{2,a}+r_{1}r_{2}\kappa_{0,ab}\left(\rho_{1}^{b}-\rho_{2}^{b}+\frac{1}{2}\;\left(r_{1}-r_{2}\right)p_{0}^{b}\right)\right). \end{array}$$

As a result, the holomorphic anomaly takes the form

(A18) $$\partial_{\bar{\tau }}{\hat{h}}_{rp_{0},\mu }=\frac{1}{8\pi i{\left(2\tau_{2}\right)}^{3/2}}\sum_{r_{1}+r_{2}=r}r_{0}\sqrt{r_{12}}\sum_{\mu_{1},\mu_{2}}\delta_{\Delta \mu \in r_{0}\Lambda_{0}}\;\vartheta_{p_{0},\mu_{12}}^{\left(r_{1},r_{2}\right)}\;{\hat{h}}_{r_{1}p_{0},\mu_{1}}{\hat{h}}_{r_{2}p_{0},\mu_{2}},$$

where $\vartheta_{v,\mu }^{\left(r_{1},r_{2}\right)}$ is a theta series similar to (the complex conjugate of) the Siegel theta series (94):

(A19) $$\vartheta_{v,\mu }^{\left(r_{1},r_{2}\right)}\left(\tau ,\bar{\tau }\right)={\left(-1\right)}^{r_{0}p_{0}^{a}\mu_{a}}\sqrt{v^{2}}\sum_{k\in r_{12}\Lambda_{0}+\mu }e^{-2\pi \tau_{2}\;\frac{k_{v}^{2}}{r_{12}}-\pi i\tau \;\frac{k^{2}}{r_{12}}}$$

and we also used the notation $k_{v}=v^{a}k_{a}/\sqrt{v^{2}}$.

Now we are ready to study what happens if $p_{0}^{2}=\left(p_{0}^{3}\right)=0$. It is clear that it is sufficient to analyze only the theta series (A19). Since at $v=p_{0}$ it is ambiguous, we define it at this point through the following limit

(A20) $$\vartheta_{p_{0},\mu }^{\left(r_{1},r_{2}\right)}:=\lim_{\varepsilon \to 0}\vartheta_{p_{\varepsilon },\mu }^{\left(r_{1},r_{2}\right)}$$

where $p_{\varepsilon }=p_{0}+\varepsilon v_{1}$ and $v_{1}$ is any vector with a positive scalar product $p_{0}\cdot v_{1}\equiv \xi >0$. Our goal will be to evaluate this limit explicitly.

To this end, it is convenient first to factorize the theta series (A19) into a product of two theta series: one defined by the two-dimensional lattice $\Lambda_{0}^{\|}=\;\mathrm{Span}\;\left\{p_{0},v_{1}\right\}$ and the second defined by its orthogonal complement in $\Lambda_{0}$, which we denote $\Lambda_{0}^{⊥}$. Since $\Lambda_{0}$ has signature $\left(1,b_{2}-1\right)$ and $p_{0}$ is a null vector, it is clear that $\Lambda_{0}^{\|}$ has signature (1,1), while $\Lambda_{0}^{⊥}$ has signature $\left(0,b_{2}-2\right)$. The idea behind this factorization is that only the first theta series will carry a non-holomorphic dependence so that we reduce our problem to a two-dimensional one. (Of course, if $\Lambda_{0}$ is two-dimensional, $\Lambda_{0}^{⊥}$ does not arise).

The problem, however, is that generically $\Lambda_{0}^{\|}⊕\Lambda_{0}^{⊥}$ does *not* coincide with $\Lambda_{0}$: not all elements of $\Lambda_{0}$ can be obtained by linear combinations with integer coefficients of the elements of $\Lambda_{0}^{\|}$ and $\Lambda_{0}^{⊥}$. A way to deal with this problem is explained in Appendix C: one should introduce the so-called glue vectors $g_{A}$, $A=0,\ldots ,n_{g}-1$, where the number of the glue vectors is determined by the cardinalities of the discriminant groups (A8). Due to this, since $\;\det\;\Lambda_{0}^{\|}=\xi^{2}$, from practical reasons it is convenient to choose $v_{1}$ that minimizes the scalar product ξ.

Applying the factorization Formula (A10) to our case, one finds

(A21) $$\vartheta_{v,\mu }^{\left(r_{1},r_{2}\right)}\left(\tau ,\bar{\tau }\right)=\sum_{A=0}^{n_{g}-1}\vartheta_{v,\mu^{\|}+r_{12}g_{A}^{\left|\right|}}^{\|}\left(\tau ,\bar{\tau }\right)\;\vartheta_{\mu^{⊥}+r_{12}g_{A}^{⊥}}^{⊥}\left(\tau \right),$$

where the superscripts ^‖^ and ^⊥^ denote the projections on $\Lambda^{\|}$ and $\Lambda^{⊥}$, respectively, and

(A22) $$\begin{array}{ccc} \vartheta_{v,\mu }^{\|}\left(\tau ,\bar{\tau }\right) & = & {\left(-1\right)}^{r_{0}p_{0}^{a}\mu_{a}}\sqrt{v^{2}}\sum_{k\in r_{12}\Lambda_{0}^{\|}+\mu }e^{-2\pi \tau_{2}\;\frac{k_{v}^{2}}{r_{12}}-\pi i\tau \;\frac{k^{2}}{r_{12}}}, \end{array}$$

(A23) $$\begin{array}{ccc} \vartheta_{\mu }^{⊥}\left(\tau \right) & = & \sum_{k\in r_{12}\Lambda_{0}^{⊥}+\mu }e^{-\pi i\tau \;\frac{k^{2}}{r_{12}}}. \end{array}$$

Representing $\Lambda_{0}^{\|}=\left\{\ell_{0}p_{0}+\ell_{1}v_{1},\;\ell_{0},\ell_{1}\in Z\right\}$ and expanding $\mu =\mu_{0}p_{0}+\mu_{1}v_{1}$, one obtains

(A24) $$k_{p_{\varepsilon }}^{2}=\frac{{\left(\left(r_{12}\ell_{1}+\mu_{1}\right)\left(\xi +\varepsilon v_{1}^{2}\right)+\varepsilon \xi \left(r_{12}\ell_{0}+\mu_{0}\right)\right)}^{2}}{2\varepsilon \xi +\varepsilon^{2}v_{1}^{2}}\;.$$

Therefore, for $v=p_{\varepsilon }$, the summand in (A22) is exponentially vanishing in the small ε limit unless $\ell_{1}=-\mu_{1}/r_{12}$. In particular, this implies that $\mu_{1}$ must belong to $r_{12}Z$. Taking into account that under this restriction on $\ell_{1}$ one has $k^{2}=0$, in the leading order, we remain with

(A25) $$\vartheta_{p_{\varepsilon },\mu }^{\|}\approx \delta_{p_{0}\cdot \mu }^{\left(\xi r_{12}\right)}\;\sqrt{2\xi \varepsilon }\sum_{\ell_{0}\in Z}e^{-\frac{\pi \tau_{2}\xi \varepsilon }{r_{12}}{\left(r_{12}\ell_{0}+\mu_{0}\right)}^{2}},$$

where $\delta_{x}^{\left(n\right)}$ is defined in (103). Now one can replace the integer variable $\ell_{0}$ by $\sqrt{\varepsilon }\ell_{0}$ so that in the limit ε→0, the summation becomes equivalent to an integral, and we arrive at the following result

(A26) $$\vartheta_{p_{0},\mu }^{\|}=\delta_{p_{0}\cdot \mu }^{\left(\xi r_{12}\right)}\;\sqrt{2\xi }\int_{R}dx\;e^{-\pi \tau_{2}\xi r_{12}x^{2}}=\delta_{p_{0}\cdot \mu }^{\left(\xi r_{12}\right)}\;\sqrt{\frac{2}{r_{12}\tau_{2}}}\;.$$

Combining it with (A18) and (A21), one reproduces the Formula (101) given in the main text.

## Appendix E. Hirzebruch and del Pezzo surfaces

The del Pezzo surface $B_{m}$ is the blow-up of $P^{2}$ over *m* generic points. It has $b_{2}\left(B_{m}\right)=m+1$, and a basis of $H_{2}\left(B_{m},Z\right)$ is given by the hyperplane class of $P^{2}$ and the exceptional divisors of the blow-up denoted, respectively, by $D_{1}$ and $D_{2},\ldots ,D_{m+1}$. In this basis, the intersection matrix and the first Chern class are given by

(A27) $$C_{\alpha \beta }=\mathrm{diag}\left(1,-1,\ldots ,-1\right),\;c_{1}\left(B_{m}\right)=3D_{1}-\sum_{\alpha =2}^{m+1}D_{\alpha }.$$

The Hirzebruch surface $F_{m}$ is a projectivization of the O(m)⊕O(0) bundle over $P^{1}$. It has $b_{2}\left(F_{m}\right)=2$ and in the basis given by the curves corresponding to the fiber [f] and the section of the bundle [s], the intersection matrix and the first Chern class are the following

(A28) $$C_{\alpha \beta }=\left(\begin{array}{cc} 0 & 1\\ 1 & -m \end{array}\right),\;c_{1}\left(F_{m}\right)=\left(m+2\right)\left[f\right]+2\left[s\right].$$

In fact, $F_{1}=B_{1}$, i.e., it is the blow-up of $P^{2}$ at one point, and by changing the basis to

(A29) $$D_{1}=\left[f\right]+\left[s\right],\;D_{2}=\left[s\right],$$

one brings $C_{\alpha \beta }$ and $c_{1}F_{1}$ to the form (A27) with m=1.

Finally, note that, for all above surfaces as well as for $P^{2}$, the signature of the intersection matrix is $\left(1,b_{2}\left(S\right)-1\right)$ and

(A30) $$c_{1}^{2}\left(S\right)=10-b_{2}\left(S\right).$$
